# RE-AIM evaluation of the national scale-up of community-based index case HIV testing in Guinea: Successes and challenges

**DOI:** 10.1371/journal.pgph.0005461

**Published:** 2026-11-10

**Authors:** Soriba Camara, Mory 1 Kourouma, Castro Gbêmêmali Hounmenou, Aly Badara Touré, Emile Faya Bongono, Sidikiba Sidibé, Tamba Mina Millimouno, Abdoulaye Sow, Alioune Camara, Alexandre Delamou

**Affiliations:** 1 Department of Public Health, Faculty of Health Sciences and Techniques, Gamal Abdel Nasser University of Conakry, Conakry, Guinea; 2 Guinea Infectious Disease Research and Training Center, Gamal Abdel Nasser University of Conakry, Conakry, Guinea; 3 Department of Computer Science, University of Labé, Labé, Guinea; 4 Africa Center of Excellence for Prevention and Control of Communicable Diseases, Gamal Abdel Nasser University of Conakry, Conakry, Guinea; 5 Department of Veterinary Medicine, Chair of Infectious and Parasitic Diseases, Higher Institute of Science and Veterinary Medicine of Dalaba, Dalaba, Guinea; Durban University of Technology, SOUTH AFRICA

## Abstract

In Guinea, an estimated 36% of people living with HIV remain unaware of their status, hindering progress toward the UNAIDS 95-95-95 targets (95% of people living with HIV to know their HIV status, 95% of those diagnosed with HIV to receive sustained antiretroviral therapy, and 95% of those receiving antiretroviral therapy to achieve viral suppression). This study evaluated the effectiveness, implementation fidelity, and sustainability of the community-based index case testing (ICT) strategy using the Reach, Effectiveness, Adoption, Implementation, and Maintenance (RE-AIM) framework. We conducted a retrospective mixed-methods evaluation (2021–2024) across 17 districts supported by 26 community-based organizations (CBOs). Quantitative analyses included mixed-effects models, district-stratified descriptive analyses, and Spearman correlations. In parallel, 28 semi-structured interviews with key stakeholders were thematically analyzed in NVivo, guided by the five RE-AIM dimensions. Among 50,609 individuals reached, 8,112 underwent HIV testing, yielding a positivity rate of 8.4% (95% CI: 7.8 to 9.0), with 84.9% (95% CI: 81.9 to 87.6) successfully linked to care. Linkage to care was substantially higher among women (93.6%) and children (90.2%) than among men (14.1%). Adoption expanded from 3 to 17 districts between 2021 and 2023, while cascade performance displayed notable inter-district heterogeneity. Qualitative findings identified strong PMTCT mentorship, community trust, and counseling continuity as key facilitators, contrasted by recurrent challenges including commodity stockouts, financial dependency, and intrahousehold disclosure barriers. The ICT strategy was associated with expanding testing uptake and high linkage to care levels under routine programmatic conditions. Maintenance findings suggest that strengthening institutional integration of CBOs, reinforcing supply chain stability, and improving financing continuity may enhance programme sustainability. These findings provide context-sensitive operational evidence that may inform the adaptation of differentiated community-based testing strategies in comparable implementation settings.

## Introduction

HIV testing remains a cornerstone of the global response, as it determines both the initiation of antiretroviral therapy and the interruption of transmission chains [1]. In 2022, an estimated 39 million people were living with HIV worldwide, with a substantial proportion still unaware of their serostatus, jeopardizing progress toward the UNAIDS 95-95-95 targets: 95% of people living with HIV knowing their status, 95% of those diagnosed receiving sustained antiretroviral therapy, and 95% of those receiving treatment achieving viral suppression [2–5]. Sub-Saharan Africa accounts for nearly two-thirds of this burden and continues to face structural and sociocultural barriers that constrain universal access to testing [2,6].

In West Africa, diagnostic coverage remains particularly low in rural areas and among young populations at risk [7–12]. To address this gap, community-based approaches have emerged as differentiated and complementary strategies to conventional health services. Among these, index case testing (ICT), involves systematic identification and testing of sexual and household contacts of persons diagnosed with HIV, aiming to improve case detection efficiency and early linkage to care [8,13–18]. Although ICT has demonstrated effectiveness in improving HIV case detection and linkage to care [19–21], evidence on its large-scale implementation and long-term integration within national health systems remains comparatively limited, particularly in low-resource settings [22–25]. Prior studies have documented important operational and contextual challenges, including stigma-related barriers, variability in programme performance, workforce constraints, and supply chain disruptions [26–28]. Understanding ICT performance under routine programme conditions is therefore essential [29,30].In Guinea, national HIV prevalence was estimated at 1.5% in 2018, yet approximately 36% of people living with HIV remain unaware of their status (approximately 43,200 individuals) [31]. Since 2019, the National HIV and Hepatitis Control Programme (PNLSH) has piloted an innovative ICT strategy anchored in prevention of mother-to-child transmission (PMTCT) sites and implemented through community-based organizations (CBOs). Despite its promise, ICT has remained marginal in practice, representing only a small fraction of tests performed outside PMTCT services [32].

ICT qualifies as a complex intervention because it involves multiple interdependent components delivered across facility and community levels, including index‑case identification, contact elicitation, community sensitization, HIV testing, referral, and linkage to care. In line with the Medical Research Council guidance on complex interventions, ICT outcomes are influenced not only by these components but also by contextual factors such as stigma, disclosure dynamics, supply‑chain reliability, and organizational capacity [5,33,34].The Reach, Effectiveness, Adoption, Implementation, Maintenance (RE-AIM) framework offers a structured approach to assess population coverage, behavioral and clinical outcomes, organizational uptake, fidelity of implementation, and sustainability [35]. This multidimensional lens is particularly relevant in resource-limited settings, where the success of an intervention depends not only on its efficacy but also on its feasibility, scalability, and institutional anchoring [22–25,33].

Against this background, the present study applied the RE-AIM framework to evaluate an index case HIV testing programme implemented in Guinea (Fig 1). The objective was to generate robust evidence on its effectiveness, adoption, and implementation processes, while identifying contextual factors influencing sustainability. Findings may provide insights relevant to comparable francophone West African settings.

**Fig 1:**
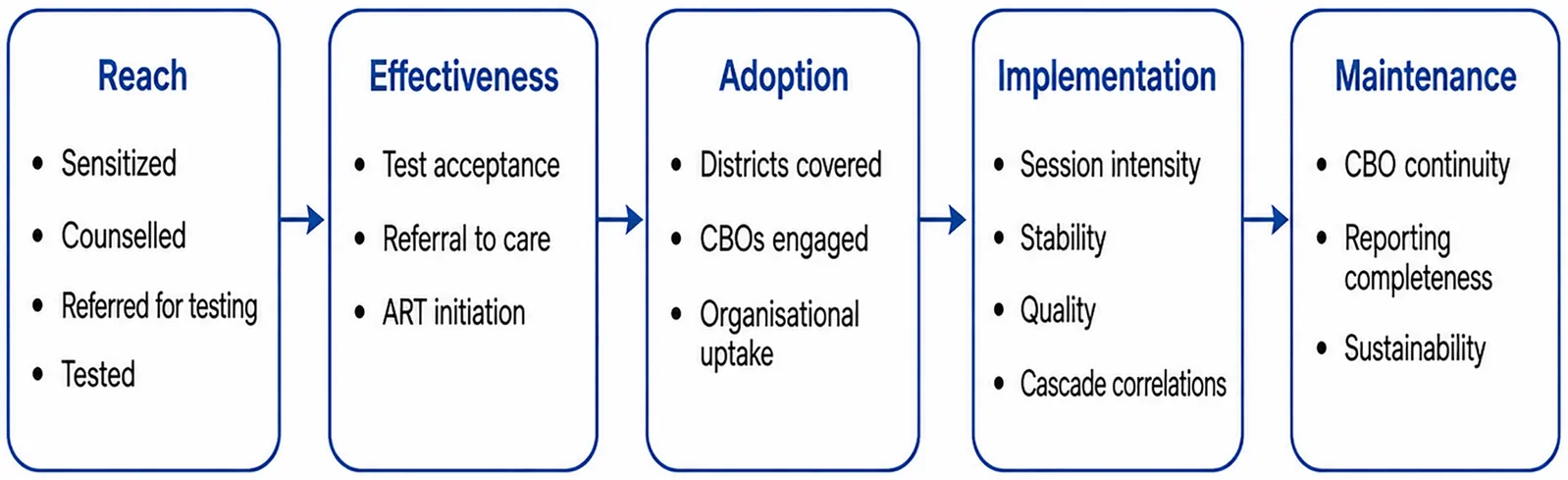
RE-AIM framework applied to the evaluation of community-based index case HIV testing in Guinea. The five dimensions: Reach, Effectiveness, Adoption, Implementation, and Maintenance, guided the identification of indicators and the interpretation of programme performance, implementation processes, and sustainability.

## Methods

### Ethics statement

The study protocol, including the secondary analysis of de-identified routine programme data, was approved by the National Ethics Committee for Health Research (CNERS, Ref. No. 060/CNERS/23). The study did not involve any clinical or interventional component but consisted of cross-sectional surveys and semi-structured interviews with participants who had experience with the community HIV testing approach. Written informed consent applied exclusively to participants recruited for the qualitative interviews. All interview participants were adults aged 18 years or older; no children or adolescents were recruited for interviews or any other research-specific data collection. All participants were informed about the study objectives, the voluntary nature of their participation, and the confidentiality measures in place. Children aged <15 years were included only through de-identified routine programme records used for secondary quantitative analysis. They were beneficiaries of standard HIV programme services and were not enrolled or contacted as research participants. Consequently, no additional research-specific parental permission or child assent was sought for the secondary analysis of these records, as covered by the CNERS-approved study protocol. Any consent procedures associated with routine HIV testing and care were governed by national programme procedures and were separate from consent for participation in this research.

For adult interview participants who were unable to read, the information sheet was read aloud in their preferred language and was documented in the presence of an independent witness. All datasets were de-identified before analysis, and no personal identifiers were retained.

### Study design

This study used a mixed-methods implementation evaluation design using routine programme monitoring data collected between 2021 and 2024. The quantitative component combined serial cross-sectional analyses of programme indicators, time-series approaches to assess inter-annual variability and trends, and longitudinal descriptive analyses to examine organizational continuity of CBOs during the implementation period. Quantitative data were analyzed from April 20 to May 18, 2025. Qualitative interviews and document reviews were conducted between December 16, 2023, and January 5, 2024. The evaluation focused on a community-based HIV ICT programme implemented in Guinea between 2021 and 2024, under the study protocol approved by the National Ethics Committee for Health Research (CNERS, Ref. No. 060/CNERS/23).

The implementation teams consisted of staff from the National HIV and Hepatitis Control Programme (PNLSH), Fraternité Médicale Guinée (FMG) as Global Fund sub-recipient, and community-based organizations (CBOs) and PMTCT sites delivering ICT activities. These actors were responsible for programme planning, service delivery, and routine reporting. In contrast, data collection and analysis were conducted by an independent research team based at the University Gamal Abdel Nasser of Conakry, who had no operational responsibility in programme implementation. These researchers designed the evaluation protocol, developed data collection tools, conducted interviews, and performed quantitative and qualitative analyses. Implementers did not participate in statistical modelling or interpretation of the findings.

The RE-AIM framework guided the evaluation, offering a multidimensional lens to assess both clinical effectiveness and implementation fidelity [35].

### Study population and sites

The study was conducted in the 17 districts in which the community-based index case testing (ICT) programme had been formally implemented during the study period. These districts were not purposively selected; rather, they reflected the progressive national rollout of the ICT strategy, which began with a pilot phase in three districts (Boké, Siguiri and N’Zérékoré) in 2021 and was subsequently expanded to 17 districts by 2023 under the coordination of the National HIV and Hepatitis Control Programme. District inclusion was therefore determined by programme implementation status, the presence of PMTCT sites involved in index-case identification, and the operation of designated CBOs within the Global Fund supported platform (S1 Fig).

The target population consisted of HIV-positive women followed in PMTCT services in these districts and their sexual and household contacts identified through the ICT. Eligible participants for qualitative interviews were adults aged 18 years or older who had direct experience with the community HIV testing strategy, either as beneficiaries or implementers. Children and adolescents were not recruited for the qualitative component of the study. The organizational units comprised of 26 community-based organizations (CBOs) operating in the participating districts. Participants were approached through CBO focal points and invited individually after being informed about the study’s objectives and confidentiality procedures. Interviews were conducted in French or local languages (Susu, Malinké, Fula, Kpelle, and Guerzé), depending on participant preference. Exclusion criteria included individuals residing outside intervention areas, those not exposed to the community-based strategy, and those who did not provide informed consent.

### Data sources and collection

Four complementary data sources were mobilized. Programmatic data consolidated from monthly reports served as the primary basis for quantitative analysis. Routine programmatic data were produced within the national HIV programme and reported by community-based organizations (CBOs) to the Global Fund sub-recipient Fraternité Médicale Guinée. These aggregated datasets included cascade indicators such as index cases identified in PMTCT sites, contacts elicited, numbers referred and tested for HIV, HIV-positive diagnoses, linkage to care, and supervision activities. These reports were completed by CBO focal persons and validated by district supervisors. In contrast, KoboCollect was used exclusively for research extraction of individual-level variables not available in routine reports, including age, sex, relationship to the index case, acceptance or refusal of HIV testing and reasons provided, place of testing, linkage outcomes, and reported operational barriers such as commodity stock-outs. KoboCollect forms were completed by trained research assistants independent from implementation teams. Programmatic documents (protocols, guidelines, supervision reports) were also reviewed to contextualize implementation processes and support data triangulation. Children aged <15 years were represented only in the routine programme records used for secondary quantitative analysis. They were not contacted, recruited, or interviewed by the research team, and no research-specific procedures were conducted with them. The quantitative dataset contained de-identified routine programme information, including age group, sex, HIV testing, and linkage indicators.

Semi-structured interviews were conducted with 28 purposively selected participants representing key actor groups involved in or affected by the intervention: (i) national-level programme coordinators (PNLSH and Fraternité Médicale Guinée), (ii) health-facility personnel from PMTCT sites, (iii) staff of CBOs (coordinators, supervisors and lay counsellors), and (iv) index cases and household members. These categories contributed respectively to policy-level perspectives, facility-community interface, operational delivery in communities, and user experience and acceptability. All individuals participating in interviews were recruited specifically for research purposes and provided written informed consent prior to data collection. Recruitment continued until thematic saturation was reached. Quantitative analyses relied on de-identified aggregated programmatic data routinely collected within the national HIV programme. Individuals who reached through community awareness, counselling, and testing activities were beneficiaries of routine service delivery and were not enrolled as research participants. Consequently, individual informed consent was not required for secondary analysis of anonymized routine data, consistent with the ethical approval obtained. All programmatic and interview data were anonymized prior to analysis, with no identifying information retained. Data were securely stored on password-protected devices accessible only to the research team. Signed consent forms are archived at the principal investigator’s institution. Interviews conducted in local languages were transcribed and translated into French before coding and analysis.

### Data quality and cleaning

Before analysis, routine records were checked for completeness, plausible ranges, and internal consistency across successive cascade numerators and denominators. Monthly reports had been completed by CBO focal persons and validated by district supervisors; the research team then In harmonized indicator labels, reporting periods, and organizational identifiers across sources. Records with impossible cascade relationships were flagged rather than automatically corrected. Four grouped observations in which the number linked to care exceeded the number diagnosed HIV-positive were excluded from the linkage model. The 2023 test-uptake estimate exceeding 1,000%, attributable to an inconsistent denominator, was classified as non-interpretable and excluded from summary interpretation. Missingness and reporting completeness were summarized at the organizational-period level; overall completeness for adoption reporting was 98.7%. The original values were retained in the audit trail, and exclusions or proxy definitions are reported alongside the relevant analyses. These procedures reduce obvious internal inconsistencies but cannot eliminate misclassification or under-reporting inherent in retrospective routine data.

### Description of the Intervention

The intervention was implemented in three sequential stages, reflecting the transition from pilot testing to operational roll-out and routine consolidation of community-based HIV index case testing in Guinea.

**Phase 1 (2021**) was the pilot phase, implemented by five CBOs across three districts (Boké, Siguiri, and N’Zérékoré). This phase focused on establishing operational protocols, building initial capacity, and testing feasibility. At this stage, PMTCT sites identified index cases from clinical registers and shared lists of partners and household contacts with CBOs. These contacts were approached for sensitization and HIV testing, conducted either at home or in community settings. Individuals who tested positive were referred to PMTCT sites or designated hospitals for confirmatory testing and treatment initiation.

**Phase 2 (2022)** was the operational adjustment and roll-out phase. During this phase, the intervention moved beyond the initial pilot platform of five CBOs in three districts and was organized around the planned scale-up platform of 26 CBOs across 17 districts. Thus, the substantial increase in programme coverage occurred between the pilot phase and the establishment of this full-scale implementation platform, rather than between Phase 2 and Phase 3. The emphasis in Phase 2 was on training, standardization of tools and referral procedures, supervision arrangements, and resource management. Fraternité Médicale Guinée (FMG), the national sub-recipient of Global Fund grants, coordinated capacity-building, supervision, and resource management. PMTCT sites ensured clinical quality assurance, while CBOs provided community-based continuity through psychosocial support, appointment reminders, and follow-up visits.

**Phase 3 (2023–2024)** was the routine implementation and consolidation phase at full scale. It did not represent a further geographic expansion beyond 17 districts or 26 CBOs; rather, it corresponded to implementation under routine programme conditions after the scale-up platform had been established. During this phase, referral, testing, follow-up, and psychosocial support mechanisms remained anchored within the established PMTCT-community interface (S2 Fig).

CBOs delivered a standardized psychosocial support package, including post-test counselling (emotional support and stigma coping), assistance with voluntary disclosure of HIV status when requested, counselling on ART initiation and adherence, accompaniment to PMTCT/ART services, and linkage to peer and community support groups. Appointment reminders were provided through a dual mechanism combining telephone calls/SMS where feasible and home visits when clients could not be contacted by phone. Reminders were triggered by missed appointments recorded in PMTCT registers according to national follow-up schedules (R1 = one month, R3 = three months, R6 = six months), with reminder actions initiated within seven days after a missed visit. Follow-up visits were conducted monthly as part of routine support and more frequently for clients at risk of loss to follow-up, those who had not disclosed their HIV status, or newly diagnosed household contacts. Visits occurred either at home, particularly when confidentiality, disclosure support, or household testing were needed, or in clinics during scheduled PMTCT/ART appointments. Priority subgroups included: (i) women lost or at risk of loss to follow-up, (ii) partners not yet tested, (iii) HIV-positive contacts not yet on ART, and (iv) women requesting assistance with disclosure.

### Operationalization of RE-AIM indicators and data analysis

The RE-AIM framework was operationalized by linking each dimension to predefined indicators, data sources, and analytical approaches (S1 Table).

***Reach*** was assessed descriptively using routine monitoring counts of individuals sensitized, counselled, referred for HIV testing, and tested. These indicators describe programme contact and cascade volume; because the size and characteristics of the total eligible contact population were unavailable, population representativeness and the proportion of all eligible contacts reached could not be estimated.

***Effectiveness*** was assessed through four distinct cascade outcomes. Testing referral was the number counselled who were referred for HIV testing divided by the number counselled. Test uptake was the number who completed HIV testing divided by the number referred for testing. Linkage to care was the number of newly diagnosed HIV-positive individuals documented as enrolled in HIV care divided by the number diagnosed HIV-positive. ART initiation was the number of diagnosed HIV-positive individuals documented as having initiated ART divided by the number diagnosed HIV-positive. Referral for testing therefore precedes diagnosis, whereas linkage to care and ART initiation are post-diagnosis outcomes and were not used interchangeably, except in the district-maturity supplementary analysis where ART initiation was explicitly used as a proxy because a separate linkage variable was unavailable. Effectiveness estimates reflect outcomes recorded in routine data and do not establish causal effects or capture unmeasured harms and longer-term retention.

***Adoption*** was assessed using the number and proportion of programme-designated districts and community-based organizations (CBOs) implementing the intervention over time. This operationalization captures organizational uptake among preselected eligible units, but not the representativeness of adopting settings, staff-level adoption, or reasons for non-adoption because no eligible unit declined participation.

***Implementation*** was assessed descriptively using implementation intensity, interannual variability of cascade volumes, and exploratory correlations between activity volumes. These measures characterize delivery patterns but do not constitute a complete fidelity assessment because standardized data on dose delivered as planned, protocol deviations, adaptation, implementation cost, and participant-level exposure were unavailable.

***Maintenance*** was assessed using CBO continuity, reporting completeness, indirect signals of supply-chain disruption, and documentary and qualitative evidence of institutional and financial sustainability. Because follow-up extended only through 2024 and commodity availability was not directly recorded at CBO level, maintenance was interpreted as short-term organizational and operational continuity rather than evidence of long-term institutionalization.

Quantitative analyses were conducted at three complementary levels of observation. Individual-level observations were used for regression models assessing determinants of cascade outcomes. CBO × year aggregates were used for organizational performance and longitudinal implementation analyses. Region × year aggregates were used for cascade correlation analyses. Descriptive analyses summarized cascade indicators across implementation phases and RE-AIM dimensions, with summary estimates presented in Table 1 and implementation-stability indicators presented in Table 2 [36–38].

**Table 1:** Effectiveness indicators of community-based HIV index testing across implementation phases in Guinea (2021-2024).

Phase		Mean testing referral rate	Mean test uptake	Mean ART Initiation
Launch (2021)		53.2%	90.2%	73.3%
Adjustment and roll-out (2022)		46.7%	83.7%	94.3%
Consolidation at scale	(2023)	3.6%	NA†	74.3%
	(2024)	75.4%	62.6%	76.4%

NA† 2023 test uptake value excluded due to a denominator reporting error producing a non-interpretable rate >1000%. Testing-referral rate was measured as the proportion of counselled individuals referred for testing. Test uptake was measured as the proportion referred individuals who completed HIV testing. These indicators are distinct from the post-diagnosis referral/linkage outcome presented in Fig 3.

**Table 2:** Interannual stability of HIV testing cascade indicators in the community-based programme, Guinea (2021-2024).

Indicator	N° years	Annual mean	Annual SD	CV (2021–2024)	Interpretation
Counseling	4	542.0	341.0	0.63	High variability
Linked to care	4	20.8	38.9	1.87	Very high variability
HIV positive	4	21.5	39.1	1.82	Very high variability
Sensitized	4	531.0	341.0	0.64	High variability
Tested	4	490.0	282.0	0.58	High variability

CV = coefficient of variation (standard deviation/mean). Values are reported as descriptive measures of relative dispersion of annual programme volumes; no universal cut-offs were applied, and higher CVs were not interpreted independently as evidence of poor implementation.

In total, six model-based quantitative analyses were conducted [36–38]. First, three individual-level mixed-effects binomial logistic regression models were fitted to assess determinants of HIV test uptake, testing referral, and ART initiation. These models accounted for the hierarchical structure of the data, with individuals nested within CBOs. Fixed effects included sex, age group, calendar year, and implementation intensity, while a random intercept at the CBO level accounted for clustering. Sex and age group were defined from routine monitoring categories. Calendar year was used to capture implementation period. Implementation intensity was operationalized as the median number of testing sessions conducted per CBO per reporting period, calculated from routine monitoring data (S3 Table).

Second, a separate grouped binomial mixed-effects logistic regression model was fitted for the subgroup analysis of linkage to care among HIV-positive individuals (Fig 3). The outcome was the number linked to care versus the number not linked among individuals diagnosed HIV-positive. Age-sex category was the explanatory variable of interest, and a random intercept at the CBO level accounted for clustering. Four observations in which the recorded number linked to care exceeded the number of HIV-positive individuals were excluded as internally inconsistent. Marginal predicted probabilities and 95% confidence intervals were derived from the fitted model using estimated marginal means.

Third, two CBO × year mixed-effects regression models were used for longitudinal organizational-level analyses of referral and linkage-to-care indicators. Repeated annual measurements were modelled with a CBO-level random intercept. Marginal predicted probabilities were derived from the fitted models as estimated marginal means, representing adjusted model-based probabilities rather than crude observed proportions. Ninety-five percent confidence intervals were calculated using model-based standard errors. Model diagnostics and technical outputs, including AIC, BIC, inter-CBO variance, and dispersion parameters, are presented in the Results section and Supporting Information (S1 text).

Exploratory associations between cascade activity volumes were summarized using Spearman rank correlations in a region × year panel for 2021–2024. Variables included aggregated counts and proportions for sensitization, counselling, testing, HIV positivity, and linkage to care. Spearman coefficients were chosen because distributions were skewed and non-normal. Given the small number of aggregated region-year observations, repeated measures within regions, multiple pairwise comparisons, and common dependence on programme scale, coefficients and nominal p values were treated as descriptive signals only; they were not interpreted as evidence of causal, temporal, or functional relationships between cascade steps (Table 3).

**Table 3:** Spearman correlation coefficients (ρ) between HIV testing cascade indicators (region × year panel, 2021–2024).

Cascade relationship (Variable 1 → Variable 2)	ρ (Spearman)	*p*-value	Strength	Expected programmatic linkage
Awareness → Counseling	0.929	<0.001	Very strong	Conversion from sensitization to counseling
Counseling → Testing	0.945	<0.001	Very strong	Expected stepwise progression
Awareness → Testing	0.917	<0.001	Very strong	Overall upstream coherence
HIV positive → Linked to care	0.946	<0.001	Very strong	Post-diagnosis referral mechanism
Awareness → Linked to care	0.592	0.020	Moderate	Indirect association
Counseling → Linked to care	0.561	0.029	Moderate	Indirect linkage via testing
Testing → Linked to care	0.542	0.037	Moderate	Reflects conversion to treatment
Awareness → HIV positive	0.492	0.063	Weak–moderate	Not significant
Counseling → HIV positive	0.466	0.080	Weak–moderate	Not significant
Testing → HIV positive	0.495	0.061	Weak–moderate	Not significant

Spearman rank correlation coefficient (ρ). Coefficients and nominal p values are reported for exploratory description only. No universal strength thresholds were applied, and no adjustment for multiple comparisons or modelling of within-region dependence was performed.

To examine whether changes in cascade performance over time reflected declining performance in districts already implementing the strategy or lower performance among newly implementing districts, we conducted an additional descriptive analysis stratified by district implementation maturity. Districts were classified as pilot districts if they had participated in the 2021 pilot phase and as scale-up districts if they entered during subsequent expansion. Cascade indicators were summarized by year and implementation-maturity group. This analysis was intended to distinguish within-site temporal changes from changes associated with the inclusion of new implementation districts (S4 Table). For the district-maturity supplementary analysis, ART initiation was used as an operational proxy for post-diagnosis linkage to care because the routine aggregated dataset used for this analysis did not contain a separate linkage-to-care variable.

Qualitative analysis followed a hybrid deductive-inductive thematic approach supported by NVivo [39]. The RE-AIM framework informed the initial coding structure, ensuring conceptual alignment with the five evaluation dimensions. Within each RE-AIM domain, inductive coding was used to identify context-specific subthemes and operational factors emerging from participant narratives [35]. Qualitative findings were used primarily to inform the Implementation and Maintenance dimensions, particularly for interpreting operational delivery, supervision, commodity availability, data-quality constraints, financing dependency, and institutional integration. They were also used to contextualize observed patterns in Reach, Effectiveness, and Adoption where relevant, but were not used to estimate quantitative cascade outcomes. The coding framework was iteratively refined through independent double coding and consensus meetings, enhancing analytic reliability (S2 Table) [40,41].

Quantitative and qualitative strands were integrated at the interpretation stage of this mixed-methods implementation evaluation. Because the qualitative interviews (December 2023–January 2024) preceded the final quantitative analyses (April–May 2025), the study was not treated as a formal explanatory sequential design. Instead, interview and documentary findings were used to contextualize programme patterns observed in the routine quantitative data, including reported supply constraints, stigma, organizational bottlenecks, supervision limitations, and data-quality challenges. All quantitative analyses were performed in R version 4.3.1.

## Results

### Reach

A total of 50,609 individuals were reached through community awareness and mobilization activities across the intervention districts. Among them, 26,025 (51.4%, 95% CI: 51.0 to 51.9) received individualized post-identification risk counseling. Of those counseled, 14,169 (54.4%, 95% CI: 53.8 to 55.0) were referred for HIV testing. Among individuals referred, 8,112 (57.3%, 95% CI: 56.3 to 58.3) completed an HIV test. The overall HIV positivity rate among those tested was 8.4% (95% CI: 7.8 to 9.0). Of individuals diagnosed with HIV, 577 (84.9%, 95% CI: 81.9 to 87.6) were successfully linked to HIV care and treatment services. Progression along the cascade is summarized in Fig 2.

**Fig 2:**
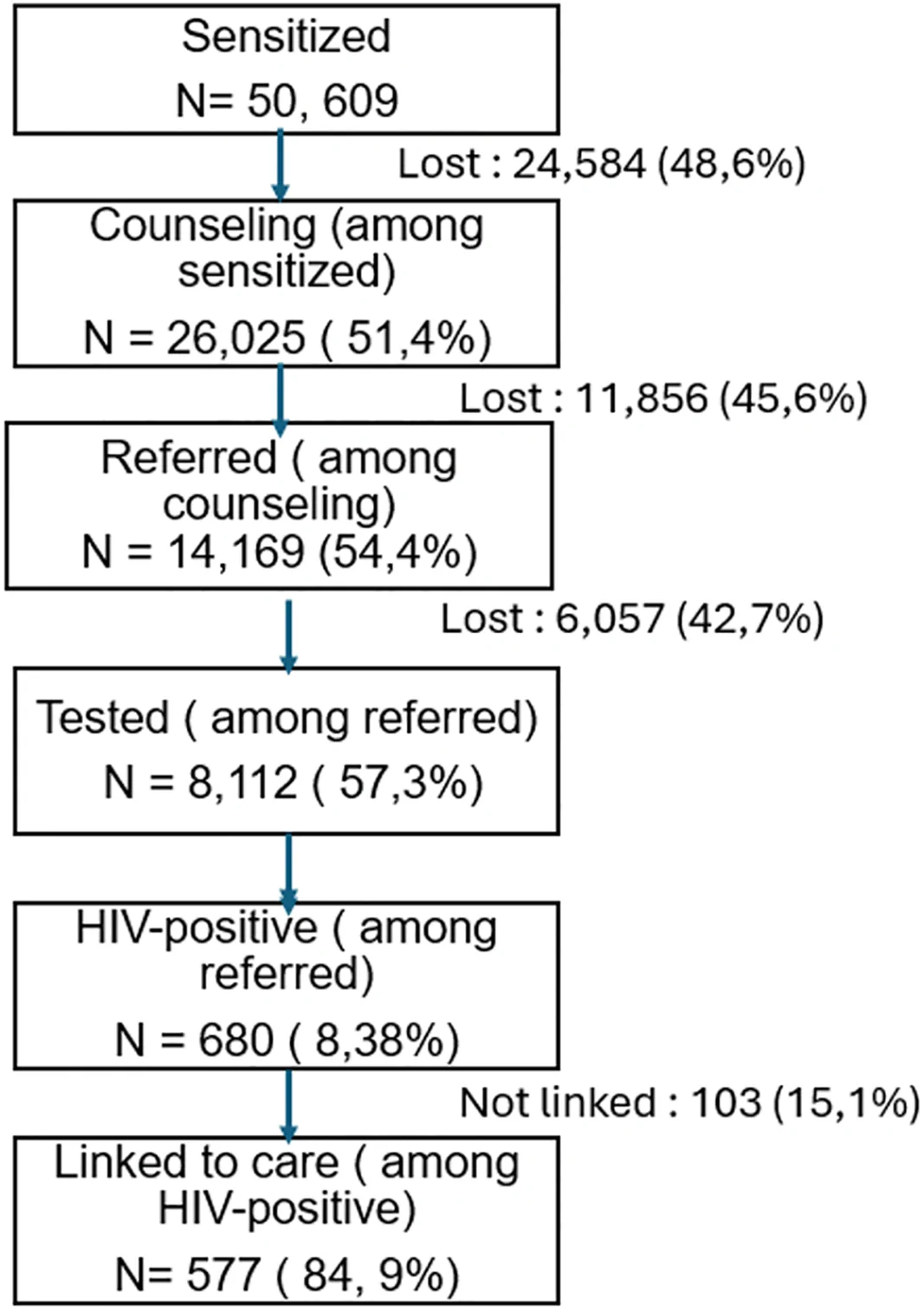
Community-based HIV testing cascade in Guinea, 2021–2024. Progression of individuals from community outreach to HIV testing and linkage to care, showing cumulative attrition across cascade stages and the proportion linked to care after HIV diagnosis.

### Effectiveness

Across phases, variation was observed for all three indicators. In 2021 (The launch phase), 53.2% of counseled individuals were referred for HIV testing, 90.2% of those referred completed testing (test uptake) and 73.3% of individuals referred to care initiated ART. In 2022 (adjustment phase), the proportion of counseled individuals referred for testing declined to 46.7%, and test uptake among referred individuals decreased to 83.7%. In contrast, ART initiation among those referred increased to 94.3%. The consolidation phase (2023–2024) showed heterogeneous patterns. The 2023 test-uptake value (>1,000%) was non-interpretable because the recorded number tested exceeded the reported referral denominator; it was therefore excluded from summary interpretation (Table 1). In 2023, the referral rate among counseled individuals dropped to 3.6%, coinciding with geographic expansion from 3 to 17 districts and from 5 to 26 CBOs. During the same year, routine monitoring data showed increased inter-annual variability in cascade indicators and a discrepancy between recorded referrals and tests performed in a subset of CBOs, as further described under the Maintenance dimension. In 2024, testing referral was 75.4% (referred/counselled), test uptake was 62.6% (tested/referred), and ART initiation was 76.4% (initiated ART/diagnosed HIV-positive). These percentages use distinct, sequential denominators and should not be read as proportions of the total population reached. In the additional descriptive analysis stratified by district implementation maturity, weighted testing-referral rates were low in 2023 among both pilot districts and scale-up districts, at 7.2% and 5.4%, respectively. In 2024, testing-referral rates increased sharply to 99.6% in pilot districts and 96.7% in scale-up districts. Test uptake estimates for 2023 were not interpreted because denominator inconsistencies produced values exceeding 100%. In 2024, test uptake was 53.3% in pilot districts and 51.9% in scale-up districts. The post-diagnosis linkage-to-care proxy, based on ART initiation among individuals diagnosed HIV-positive, was 99.1% in pilot districts and 82.5% in scale-up districts in 2023, and 92.9% and 87.4%, respectively, in 2024 (S4 Table).

A separate subgroup analysis examined linkage to care among individuals identified as HIV-positive. The adjusted probability of linkage was 93.6% among women (95% CI: 88.8 to 96.5; n = 389), 90.2% among children aged <15 years (95% CI: 80.2 to 95.5; n = 238), and 14.1% among men (95% CI: 7.1 to 26.1; n = 109). The mixed-effects binomial model accounting for clustering at the CBO level showed markedly lower linkage to care among men (Fig 3). These values represent model-based marginal predicted probabilities rather than crude proportions. The subgroup sample sizes correspond to the total numbers of HIV-positive individuals represented in the grouped binomial observations retained by the fitted model.

**Fig 3:**
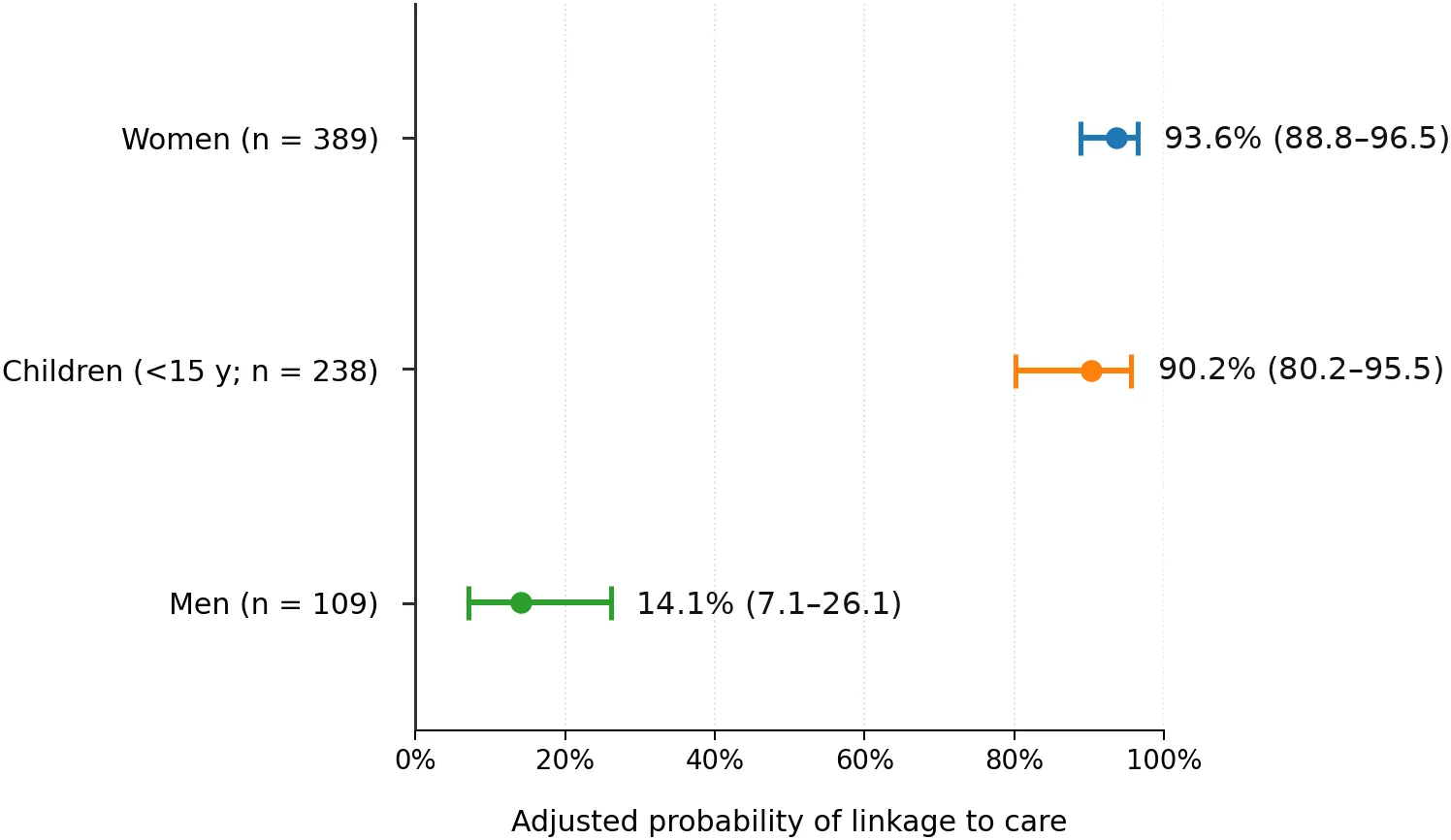
Marginal predicted probabilities of linkage to care among HIV-positive individuals by age group and sex. Adjusted probabilities were derived from a mixed-effects binomial logistic regression model with age-sex category as a fixed effect and a random intercept at the CBO level. Sample sizes represent the numbers of HIV-positive individuals contributing to the fitted model: women, n = 389; children aged <15 years, n = 238; and men, n = 109. Points represent marginal predicted probabilities, and horizontal bars represent 95% confidence intervals.

### Adoption

The intervention expanded from 3 to 17 participating districts and from 5 to 26 implementing CBOs between 2021 and 2023, corresponding to 5.7-fold and 5.2-fold increases, respectively.

At the planning stage in 2020, 26 CBOs across 17 districts had been formally designated as eligible implementers by the national programme. By 2023, all 26 eligible CBOs were implementing the intervention, corresponding to 100% organizational adoption among designated eligible sites. The fold changes describe expansion relative to the 2021 pilot footprint, whereas the 100% estimate uses the predesignated eligible CBOs as its denominator. No eligible CBO or district declined participation. Completeness of adoption reporting was 98.7% over the study period (Fig 4).

**Fig 4:**
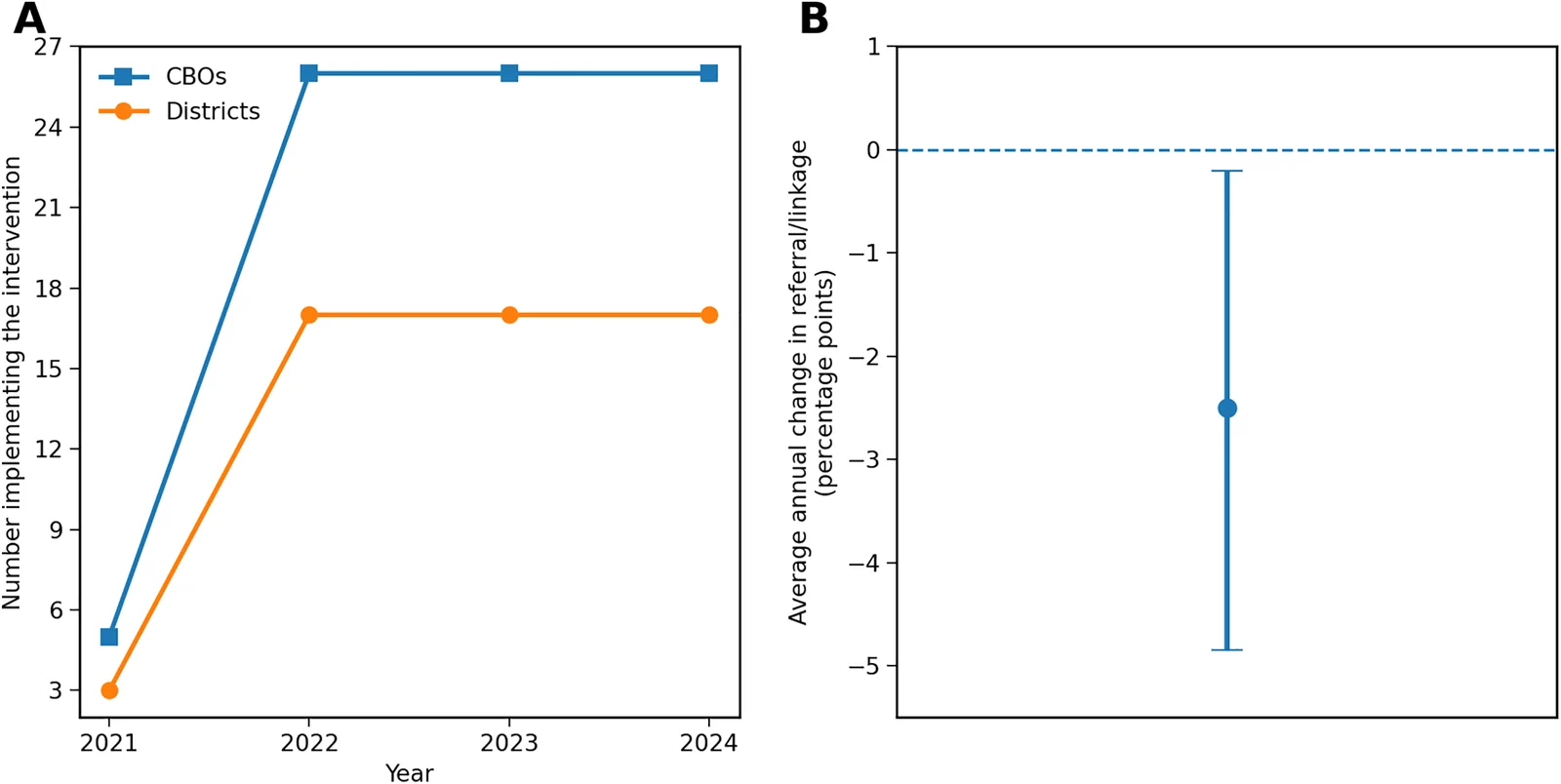
Adoption and longitudinal change in referral/linkage indicators. **(A)** Scale-up of community-based index testing, showing the number of districts and CBOs implementing the intervention (2021–2023). **(B)** Average annual change in referral/linkage estimated from mixed-effects regression with calendar year as time variable and a CBO-level random intercept; points show model-based estimates and bars 95% confidence intervals (percentage-point change).

Longitudinal changes in referral and linkage indicators were assessed using mixed-effects regression models, with calendar year entered as a time variable and a random intercept at the CBO level to account for repeated measurements within organizations. This analysis showed a statistically significant average annual decline in referral and linkage to care, estimated at −2.54% per year (95% CI: −4.86 to −0.21; p = 0.033).

### Implementation

The implementation unfolded in three operational phases (Table 4, S3 Table): an initial high-intensity phase in 2021, characterized by rapid mobilization and high activity volumes; an adjustment phase in 2022, marked by stabilization of procedures; and an expansion phase in 2023, corresponding to the geographic scale-up of community-based organizations (CBOs).

**Table 4:** Summary of quantitative and qualitative findings related to Maintenance and implementation conditions, Guinea, 2021-2024.

Element	Indicator / Quantitative evidence	Qualitative corroboration	Illustrative verbatims	Suggested actions
**Organizational stability**	26 active CBOs; median tenure = 3 years (2022–2024)	Implementers highlighted sustained CBO engagement and local legitimacy	*“The advantage we had was selecting NGOs that were already active in the field.”* (Coordinator 1)	Formalize CBO roles in district health plans; establish MOUs and routine supervision schedules
**Data coherence**	~19.2% of CBOs annually showed referral-to-test discrepancies; 7.7–11.5% presented atypical intra-annual tested/referred fluctuations	Staff reported mismatches between programme databases and field realities	*“At the beginning we did not have a well-established strategy; we had to adapt the health centre tools.”* (Coordinator 1)	Strengthen routine data validation, introduce triangulation checks, and train CBO data focal points
**Commodity security**	No official programmatic stockout records; implementers nevertheless reported intermittent supply interruptions	Several implementers cited irregular HIV test kit supply as affecting continuity	*“The biggest problem we had was with inputs, since some health centre managers were reluctant to supply CBOs.”*	Institutionalize supply chain reporting at CBO level; establish buffer stocks and rapid resupply procedures
**Financial sustainability**	Ongoing dependence on external funding reported	Concerns raised about continuity if external support declines	*“After CBO training in 2020, activities only began in 2021 because we had not received funding.”*	Develop phased domestic financing plans; diversify funding and formalize cost-sharing mechanisms
**Human resources and supervision**	Reports of variable training quality, high turnover, and limited supervision	Implementers highlighted gaps in refresher training and logistical support	*“There are many changes among community counselors; sometimes allowances are delayed, and newcomers need training.”*	Implement regular refresher training, standardized supervision tools, and retention incentives
**Confidentiality and stigma**	Identified as a constraint for linkage and test uptake	Fear of disclosure within households frequently reported	*“Only my children know I am sick, because if other family members knew, I would be rejected.”* (Index case 6)	Strengthen confidentiality protocols, counselor training, and community stigma-reduction activities
**Community ownership**	Continued involvement of local leaders and CBOs documented	Reports described local leaders organizing awareness sessions and enhancing legitimacy	*“When we enter a community, before starting sensitization, we approach the local leaders; they give us the green light and their support.”* (CBO 11)	Sustain and formalize community leadership roles; involve local leaders in performance reviews

Descriptive interannual variability was observed in upstream cascade volumes: sensitization (CV = 0.63), counselling (CV = 0.63), and testing (CV = 0.58). HIV-positive diagnoses (CV = 1.82) and linkage-to-care counts (CV = 1.87) varied more relative to their means (Table 2); these CVs describe dispersion of volumes and do not, by themselves, measure implementation fidelity or instability. In the aggregated region × year panel, Spearman coefficients were >0.90 for sensitization, counselling, and testing, and 0.946 for HIV-positive diagnoses versus linkage-to-care counts. Coefficients for counselling and testing versus linkage-to-care counts were 0.561 and 0.542, respectively (Table 3). Because these exploratory analyses used a small aggregated panel and indicators sharing programme-volume effects, no causal or mechanistic interpretation was assigned to coefficient magnitude or nominal p values.

### Maintenance

Between 2022 and 2024 the community-based network remained stable, with 26 active CBOs and a median operational tenure of three years. Programmatic records did not directly document the availability of testing commodities at the CBO level. Nevertheless, several indirect signals of operational instability were detected. Each year since 2022, approximately 19.2% of CBOs showed a discrepancy between the number of recorded referrals and the number of tests performed. Likewise, 7.7% to 11.5% of CBOs exhibited atypical intra-annual fluctuations in the tested-to-referred ratio. These quantitative patterns were further contextualized by qualitative findings, which also informed other RE-AIM dimensions (Reach, Effectiveness, and Implementation), but were particularly critical for interpreting sustainability-related instability signals. Multiple participants reported structural and organizational constraints affecting service continuity, notably irregular commodity supplies and dependence on external funding. Taken together, these findings indicate that “maintenance” was achieved in terms of organizational presence and programme footprint, but not uniformly in terms of routine operational performance. The divergence between standardized databases and implementers’ perceptions underscores the need for systematic triangulation of information sources, improved data quality systems, and stronger supply-chain monitoring to accurately assess the stability and sustainability of community-based interventions (Table 4).

### Mechanisms of impact of the intervention

The five RE‑AIM dimensions describe multidimensional programme dynamics. Expansion of coverage (Reach) was accompanied by household exposure to HIV testing activities, whereas Effectiveness indicators displayed heterogeneous patterns. Linkage to care was substantially higher among women and children than among men, revealing a marked sex-related gap in engagement with the care pathway following an HIV-positive result. Across the RE-AIM dimensions, periods of programme expansion, reflected by increased organizational adoption and geographic coverage, were temporally associated with higher activity volumes, while also coinciding with greater variability in effectiveness and maintenance indicators. Implementation patterns evolved through phases of intensity, adjustment, and expansion, with performance varying in relation to session intensity and inter‑CBO stability. Qualitative analyses suggested several operational factors perceived to support programme functioning, including community counseling, PMTCT mentorship, and longitudinal follow‑up, that enhanced trust and continuity of care. They also identified recurrent structural and operational constraints, notably, commodity stockouts, reliance on external funding, and difficulties with intra‑household disclosure. These factors were perceived by participants as influencing the conversion of sensitization into testing uptake and linkage to care. Integrated quantitative – qualitative triangulation helped identify patterns not fully captured by routine indicators. Taken together, these observations suggest that sustaining programme performance may depend on balancing coverage expansion with operational stability, including strengthening supply chain reliability and institutional support mechanisms (S3 Fig).

## Discussion

This study, guided by the RE‑AIM framework, evaluated the implementation and effectiveness of an index‑assisted community HIV testing strategy in Guinea from 2021 to 2024. The intervention reached more than 50,000 individuals and achieved broad organizational adoption, but performance varied across cascade steps and implementation phases. Referral and test uptake fluctuated during scale-up, whereas linkage to care and ART initiation remained generally high among individuals diagnosed HIV-positive, with markedly lower linkage observed among men than among women and children. Three principal findings frame the discussion. First, effectiveness was uneven, particularly for referral and test uptake during scale-up and for linkage among men. Second, the intervention followed a three-phase implementation trajectory, moving from high-intensity piloting to operational adjustment and then to routine implementation and consolidation at full scale. Third, despite broad organizational adoption, institutional sustainability remained fragile because of persistent operational, financing, supply-chain, and data-system constraints.

Subgroup analysis revealed a marked sex-related gap in post-diagnosis linkage to care. The substantially lower adjusted probability of linkage among men represents an important implementation gap. Comparable studies in African settings have documented lower engagement of men in HIV testing and care pathways than women, reflecting gender-related differences in healthcare use, stigma, disclosure concerns, and service accessibility [42,43]. In the present study, qualitative findings concerning stigma and difficulties with intra-household disclosure provide plausible contextual explanations. Because HIV-positive women were identified through PMTCT services, the intervention pathway may also have shaped male engagement. Male partners may have perceived a PMTCT-anchored intervention as primarily oriented toward women and children, reducing their sense of direct relevance or comfort with engaging in the care pathway. Broader social norms around masculinity, health-seeking behaviour, and HIV-related stigma in Guinea may also have contributed to delayed or incomplete engagement among men. These explanations should be interpreted cautiously, because the evaluation did not collect male-specific qualitative data on reasons for non-linkage. The observed difference should therefore not be interpreted as direct evidence that men refused to accept their HIV status, but rather as evidence of lower male engagement with the post-diagnosis care pathway, potentially reflecting individual, household, community, and service-level barriers.

Lower male engagement may reduce opportunities for partner support and complicate voluntary and safe disclosure within households. Nevertheless, this evaluation did not assess women’s retention according to their partners’ referral status or measure intimate-partner or gender-based violence outcomes; no causal relationship can therefore be inferred. Male-responsive strategies, including trained male peer navigators or trusted community champions, confidential counselling, flexible service-delivery modalities, and supported referral, should be prospectively evaluated. Such approaches should also incorporate voluntary disclosure options, confidential assessment of disclosure-related safety, and appropriate referral pathways for individuals at risk of violence [44–46].

The implementation findings presented in Tables 2 and 3 provide additional insight into why cascade performance varied during routine scale-up. Table 2 shows high interannual variability in upstream cascade activities, including sensitization, counselling, and testing, and very high variability in downstream indicators such as HIV positivity and linkage to care. This suggests that implementation intensity and cascade conversion were not stable over time. Table 3 further shows very strong correlations between sensitization, counselling, and testing, indicating that the upstream cascade was operationally coherent when community activities were delivered. However, the more moderate correlations between upstream activities and linkage to care suggest that the transition from community testing to post-diagnosis care was more vulnerable to operational and social barriers, including referral coordination, commodity availability, stigma, and disclosure constraints. This interpretation is consistent with studies from Burkina Faso and other West African settings, where irregular commodity availability, stigma-related barriers, and weak referral coordination have been shown to limit the conversion of community-based HIV testing into effective linkage to care [47–49].

The phase-level pattern refers to changes in cascade performance across the three implementation stages documented in the Results. As shown in Table 1, the intervention moved from a high-intensity pilot phase in 2021, to an organizational adjustment phase in 2022, and then to routine expansion in 2023–2024, with substantial fluctuations in testing referral and test uptake. The additional district-stratified analysis presented in S4 Table further showed that the 2023 reduction in testing referral occurred in both pilot and scale-up districts, followed by improvement in both groups in 2024. This finding suggests that the 2023 decline should not be interpreted solely as an effect of newly added districts or territorial expansion. Rather, it is more consistent with broader programme-level instability during scale-up, including counsellor turnover, commodity-supply interruptions, supervision constraints, and data-validation challenges. Expansion may nevertheless have contributed to greater variability in diagnostic yield and cascade conversion, but routine programme data do not allow the relative contribution of expansion-related effects and broader operational disruptions to be quantified. Similar challenges have been reported in community-based HIV testing programmes in West and Southern Africa, where rapid expansion without sustained supervision, reliable commodity supply, and adaptive targeting may reduce the consistency of cascade performance and diagnostic yield [50–52].

Sustainability of the community testing model in Guinea depends on two interdependent conditions: the long‑term viability of external financing amid declining international aid, and formal integration of the approach within national health systems. This dual dependence creates structural fragility that has undermined community interventions elsewhere in sub‑Saharan Africa when external funding ceased [53,54]. In Guinea, the lack of institutionalized supply mechanisms for CBOs exacerbated vulnerability: intermittent commodity shortages were reported by several implementers, despite no documented “stockout” events in routine programme databases. This discrepancy exposes limitations of standard monitoring indicators and underscores the value of systematic triangulation between quantitative records and qualitative inquiry to detect operational interruptions. The RE-AIM framework highlighted that programme expansion, reflected by increased organizational adoption and geographic coverage, coincided with higher reported activity volumes, while also revealing variability in effectiveness and maintenance indicators. These multidimensional tensions would likely have remained undetected in evaluations focused solely on biomedical outcomes.

This study has several methodological limitations. Its retrospective design and reliance on routine programmatic data may have introduced reporting and completeness biases. The study did not directly examine the reasons why HIV-positive men were not linked to care, assess women’s treatment retention according to their partners’ referral status, or measure adverse social outcomes such as unwanted disclosure, intimate-partner violence, or other forms of gender-based violence. The findings therefore identify a substantial male-engagement gap but do not establish its underlying mechanisms or its consequences for female partners’ treatment outcomes. The absence of a contemporaneous comparison group limits causal inference, and residual inter-site variability suggests that unmeasured contextual factors, such as local coordination capacity, supply consistency, and population mobility, may have influenced outcomes. Nevertheless, the study presents notable strengths, including broad geographic coverage across 17 districts, methodological triangulation combining quantitative and qualitative data, and the structured application of the RE-AIM framework to evaluate a national-scale community-based HIV testing strategy.

The RE-AIM operationalization also had dimension-specific limitations. Reach could not assess representativeness or the proportion of all eligible contacts reached; Adoption was restricted to predesignated organizations and did not assess staff-level uptake; Implementation relied on activity intensity and variability rather than comprehensive fidelity, adaptation, and cost measures; and Maintenance represented short-term continuity through 2024, without long-term follow-up or direct CBO-level commodity records. Accordingly, the findings provide a pragmatic assessment based on available routine indicators rather than a complete measurement of every RE-AIM construct.

Policy and programmatic implications follow directly. Findings from the Maintenance dimension indicate that long-term sustainability remains vulnerable due to reliance on external funding, incomplete integration into national budgeting mechanisms, and gaps in commodity supply reporting at the CBO level. Strengthening institutional integration within district health planning processes and national financing frameworks may enhance stability of community-based index testing. Structural integration of community-based organizations into the formal health system can enhance service reach among hard-to-reach and at-risk populations, while reducing attrition along the cascade. Programmes should consider a male-responsive post-diagnosis support package combining trained male peer navigators or trusted community champions, confidential counselling, flexible service hours and referral options, and active accompaniment to HIV care. Because partner engagement and disclosure may raise safety concerns, these approaches should remain voluntary and include confidential assessment of disclosure-related risks and functional referral pathways for psychosocial support and protection. Their acceptability, effectiveness, and potential unintended consequences should be assessed prospectively. Financial sustainability requires the progressive incorporation of operational costs into national health budgets and proactive mobilization of domestic co-financing mechanisms to reduce dependence on external donors.

Future research should assess the cost-effectiveness of this model to guide evidence-based resource allocation. Future studies should prospectively evaluate male-responsive and peer-supported strategies using sex-disaggregated outcomes for post-diagnosis referral, linkage to care, ART initiation, and retention. They should document male-specific reasons for engagement or non-engagement, examine whether male partner linkage is associated with women’s retention in care, and systematically monitor unintended social harms, including unwanted disclosure and intimate-partner or gender-based violence. Quasi-experimental studies including comparison groups could strengthen causal inference, while targeted qualitative analyses may identify the most effective operational levers to sustain performance during scale-up. Longitudinal cohort studies are also warranted to evaluate long-term outcomes in treatment adherence and retention.

## Conclusion

Overall, the findings show mixed programme performance. The community-clinical index testing model achieved broad reach, wide organizational adoption, and generally high linkage to care and ART initiation among individuals diagnosed HIV-positive, particularly among women and children. However, performance was not uniformly strong across the cascade. Testing referral and test uptake fluctuated during scale-up, linkage to care was markedly lower among men, and sustainability remained constrained by commodity-supply interruptions, data-system limitations, reliance on external financing, and incomplete institutional integration of CBOs. These findings support the continued adaptation of community-based index testing in Guinea, but indicate that scale-up should be accompanied by stronger operational support, male-responsive linkage strategies, more reliable supply-chain mechanisms, and sustainable financing arrangements.

## Supporting information

S1 Fig Geographic distribution of intervention sites for community-based HIV testing in Guinea (2021–2024). Pilot and scale-up districts implementing community index testing through community-based organizations (CBOs) linked to PMTCT sites. Map produced in QGIS v3.44.4 using administrative boundaries from GADM (https://gadm.org/; https://gadm.org/license.html).
(TIF)

S2 Fig Programme theory linking PMTCT sites and community-based organizations (CBOs) within the HIV index case testing cascade in Guinea (2021–2024). The figure illustrates the hypothesized pathways connecting community sensitization, testing, and link.
(TIF)

S1 Table Operationalization of RE-AIM. Definition of the RE-AIM dimensions and corresponding indicators, including calculation methods, data sources, and analytical approaches used to evaluate the community-based HIV index case testing programme in Guinea (2021–2024).
(DOCX)

S1 Text Detailed statistical analysis. Description of study variables, mixed-effects modelling approach, diagnostics, sensitivity analyses, and statistical software/packages used to assess HIV test acceptance, referral, and ART initiation outcomes (Guinea, 2021–2024).
(DOCX)

S2 Table NVivo codebook aligned with the RE-AIM framework. Qualitative coding framework used for thematic analysis, including operational definitions of codes by RE-AIM dimension and representative illustrative quotes from key informants and beneficiaries.
(DOCX)

S3 Table Implementation phases of the community-based HIV testing programme. Summary of implementation phases (2021–2024), including intensity, operational objectives, key mechanisms, and expected quantitative signals used to interpret programme performance over time.
(DOCX)

S3 Fig Mechanism of impact of community-based organizations in the index case HIV testing cascade (Guinea, 2021–2024). Conceptual diagram illustrating how CBO activities contribute to key cascade steps, from sensitization and contact elicitation to testing, referral, and linkage to care.
(TIF)

S1 Data De-identified aggregated dataset and codebook (XLSX). Anonymized aggregated quarterly programme dataset used for quantitative analyses of community-based index case HIV testing in Guinea (2021–2024), including codebook and README.
(XLSX)

S4 Table Cascade performance by district implementation maturity, Guinea, 2023–2024. Weighted aggregate cascade indicators comparing pilot districts and scale-up districts during 2023–2024. Rates were calculated by pooling numerators and denominators within each implementation-maturity group and year. Test uptake estimates for 2023 were not interpreted because denominator inconsistencies produced values exceeding 100%.
(DOCX)
