# Supplementary material for: RE-AIM evaluation of the national scale-up of community-based index case HIV testing in Guinea: Successes and challenges
Source: PLOS Glob Public Health. 2026 Nov 10;6(11):e0005461. doi: 10.1371/journal.pgph.0005461 (PMC13750574; doi:10.1371/journal.pgph.0005461)

Response to Reviewers

Manuscript ID: PGPH-D-25-03261
Title: *RE-AIM evaluation of the national scale-up of community-based index case HIV testing in Guinea: Successes and Challenges*
Journal: PLOS Global Public Health

Dear Editor,
Dear Reviewer,

We would like to thank you for the opportunity to revise our manuscript and for the constructive editorial and reviewer comments. We have carefully revised the manuscript to address all points raised. Major revisions include: clarification of study design and participant roles, clarification of programme rollout and district selection, strengthened description of intervention components, improved reporting and interpretation of anomalous indicators, detailed description of statistical models (including marginal predicted probabilities and longitudinal analyses), revision of adoption indicators and presentation of Figure 3, and strengthened policies-related sections (funding, competing interests, data availability, copyright).

Below we provide a point-by-point response. All changes have been incorporated into the revised manuscript and highlighted in the tracked-changes version.

A. Editorial Requirements

Editor Comment 1. Financial Disclosure statement

Comment: Please amend your detailed Financial Disclosure statement. It must be completed in full sentences and contain the exact wording you wish to be published.

Response:
Thank you for this request. We revised the Financial Disclosure / Funding Statement so that it is written in complete sentences and includes the exact wording intended for publication. The updated statement has been inserted in the manuscript under the "Funding" section.

Editor Comment 2. Clarify all sources of funding

Comment: Please clarify all sources of funding (financial or material support). List grants (with grant number) or organizations (with URL), including institutional funding.

Response:

We confirm that the study received financial support from the WHO Special Programme for Research and Training in Tropical Diseases (TDR) under the support programme for early-career researchers in Francophone Africa (Grant No. V1 20170329), administered through Cheikh Anta Diop University of Dakar (Senegal) and the Africa Centre of Excellence at Université Gamal Abdel Nasser de Conakry (Guinea). We confirm no additional external grants or material support were received. The Global Fund supported the implementation platform of routine HIV services in Guinea but did not fund this evaluation study.

Editor Comment 3. Funding initials

Comment: State the initials, alongside each funding source, of each author to receive each grant.

Response:

Funding was received from WHO/TDR (Grant No. V1 20170329). The author who received this grant was SC (Soriba Camara). All other co-authors (MK, CGH, ABT, EFB, SS, TMM, AS, AC, AD) did not receive funding from this grant.

Editor Comment 4. Role of funders

Comment: State what role the funders took in the study; if none, state the required wording.

Response:

The funders had no role in study design, data collection and analysis, decision to publish, or preparation of the manuscript.

Editor Comment 5. Salary from funders

Comment: If any authors received a salary from any funders, state which authors and which funders.

Response:

No authors received a salary from any funders, including WHO/TDR. The TDR funding

supported research activities (e.g., data collection) and did not include salary payments to any author.

Editor Comment 6. Competing Interests

Comment: Please send a completed Competing Interests statement including any declared COIs; if none, include the required wording.

Response:
All authors have completed the PLOS competing interests requirements. The authors have declared that no competing interests exist.

Editor Comment 7. Data availability

Comment: All data underlying findings must be freely available via repository, manuscript, or supplementary information.

Response:
We updated the Data Availability Statement and provided the underlying de-identified aggregated dataset as Supporting Information, in Excel . The dataset includes no individual-level identifiers and complies with research ethics board-approved protocol and national confidentiality requirements.

Editor Comment 8. Supplementary material formatting

Comment: Supplementary figures/tables must be removed from the manuscript and uploaded as Supporting Information; legends listed after references.

Response:
We revised the manuscript accordingly. Supplementary figures/tables were removed from the main manuscript and uploaded separately as Supporting Information, with legends listed after the References section.

Editor Comment 5. Potential copyright issues : S1 Fig map

Comment: Some material included in your submission may be copyrighted. For S1 Fig, please provide a direct link to the map base layer (shapefile) and a link to the license/terms of use, ensuring compatibility with CC BY 4.0.

Response:
Thank you for raising this important point. We confirm that S1 Fig was created by the

authors using **QGIS (version 3.44.4)** and was not obtained from Google Maps, Mapquest, or any other proprietary basemap.

The map base layer (country and administrative boundaries) was generated from openly accessible administrative boundary shapefiles sourced from GADM (Database of Global Administrative Areas). We have now explicitly added the shapefile source and license/terms-of-use links in the figure legend to ensure full compliance with PLOS copyright and CC BY 4.0 licensing requirements.

- **Mapping software used:** QGIS v3.44.4
- **Basemap / boundary shapefile source:** GADM (Database of Global Administrative Areas)
- **Direct link to shapefile/data source:** https://gadm.org/
- **License / terms-of-use link:** https://gadm.org/license.html

No copyrighted cartographic material was used. The resulting map is therefore suitable for publication under the Creative Commons Attribution 4.0 International (CC BY 4.0) license used by PLOS.

B. Reviewer 1 Comments

Reviewer Comment 1. Clarification of independent data collection (Line 105)

Comment: Clarify who constituted implementation teams and relationship between implementers and data collectors/analysis team.

Response:
We clarified the distinction between implementation teams (PNLSH/FMG/CBO field implementers) and the research team. We revised the Study Design section to explicitly state that data collection and analyses were conducted by independent research assistants/researchers not involved in programme delivery. Relevant clarifying text has been added to the Methods section.

Reviewer Comment 2. Selection of participating districts (Line 112)

Comment: Explain rationale for choosing 17 districts (program presence, burden, convenience, etc.).

Response:

We clarified that districts were included based on the real-world rollout of the national community-based index testing strategy coordinated by PNLSH, rather than convenience sampling or burden-based selection. We specified: pilot in Boké, Siguiri, and N'Zérékoré; scale-up to 17 districts; inclusion restricted to districts with official implementation capacity (PMTCT sites + operational CBOs within Global Fund platform). We revised the Study Population and Sites section accordingly and referenced the geographic distribution in S1 Fig.

Reviewer Comment 3. Data sources and reporting procedures

Comment: Distinction between routine data and KoboCollect requires clarification; specify what info captured in each; who completed each dataset.

Response:

We expanded Methods to clearly distinguish: (i) routine quarterly programme monitoring data reported by CBO focal points to FMG (Excel database), and (ii) research-specific KoboCollect dataset entered by independent research assistants during field visits, capturing individual-level/contextual variables not contained in routine monitoring. We specified staff responsible for each source and described triangulation procedures to ensure consistency.

Reviewer Comment 4. Roles of study participants (Line 130)

Comment: 28 participants' roles are vague; detail involvement.

Response:

We provided a clear breakdown of the 28 interviewees across four functional groups (PNLSH/FMG coordination, PMTCT health workers, CBO staff, beneficiaries/index cases and household members) and explained their contributions. The manuscript Methods now includes these roles to improve transparency and replicability.

Reviewer Comment 5. Geographic scope (Line 141)

Comment: Specify which "three districts" are referred to.

Response:

We revised the text to explicitly name the pilot districts: Boké, Siguiri, and N'Zérékoré.

Reviewer Comment 6. Description of interventions and support provided by CBOs (Line 151)

Comment: Add detail on psychosocial support, appointment reminders, criteria, follow-up visits.

Response:
We expanded the intervention description: psychosocial support components (post-test counselling, stigma coping, disclosure support, navigation/accompaniment, peer support), reminder mechanisms (phone/SMS and home visits), triggers (missed PMTCT schedule R1/R3/R6; reminders initiated within 7 days), and follow-up modalities (monthly routine, intensified for risk of LTFU, disclosure needs, newly diagnosed contacts; home/clinic-based). Changes were inserted into Methods.

Reviewer Comment 7. Testing acceptance in 2023 (value >1,000%)

Comment: Anomalous value not interpretable; remove/correct and explain.

Response:
We confirmed that the 2023 testing acceptance value was due to denominator reporting error in routine program data, producing a non-interpretable outlier (>1,000%). We excluded this value from summary analyses and explicitly documented the issue in Table 1 footnote and Results text.

Reviewer Comment 8. Low referral rate in 2023

Comment: Explain contextual/operational factors contributing to drop.

Response:
Thank you for this important comment. We have revised the manuscript to better contextualize the sharp decline in referral observed in 2023. In the **Results**, we now report this decline descriptively and document concurrent signals of operational instability during the rapid scale-up phase (including increased inter-annual variability and referral-to-testing discrepancies). In the **Discussion**, we added plausible contributing factors supported by qualitative interviews, including turnover of community counsellors, recurrent interruptions in HIV test commodity supply, and increased administrative workload during preparation of the Global Fund 2024–2026 funding request, which may also have affected routine reporting and validation. We

also acknowledge that routine program data do not allow quantifying the relative contribution of each factor.

Reviewer Comment 9. Figure 2 – marginal predicted probabilities definition

Comment: Clarify whether these are model-based or empirical.

Response:
We clarified that Figure 2 values are model-based adjusted probabilities derived from mixed-effects binomial logistic regression, and computed as estimated marginal means (averaging over covariate distributions). This clarification was added to Statistical Analysis, Results, the figure legend and S4.

Reviewer Comment 10. Longitudinal models (Lines 261–264)

Comment: Describe model specifications: type, time, covariates, repeated measures.

Response:
We revised Statistical Analysis and Supplement S4 to describe longitudinal models explicitly (mixed-effects regression with repeated annual measurements at CBO level; calendar year parameterization; covariates including intervention intensity; random intercept for CBO to handle repeated measures; interpretation of annual change as coefficient of time variable). A short explanatory sentence was added in Results to clarify interpretation of estimated annual change.

Reviewer Comment 11. Adoption indicators and Figure 3C

Comment: Define adoption indicators; Figure 3C limited value; report proportion of eligible sites adopting; any eligible sites declined?

Response:
We clarified adoption definitions at district and organizational level, and reported that 26 CBOs across 17 districts were identified as eligible in 2020; scale-up was progressive; by 2023 all 26 eligible CBOs implemented the intervention (100% adoption among eligible sites), and no eligible CBOs/districts declined participation. We revised Adoption Results accordingly. We removed the previous Figure 3C panel on data completeness due to limited added value and reported completeness descriptively in text.

We hope that the revised manuscript satisfactorily addresses all editorial and reviewer comments and improves the clarity, methodological transparency, and replicability of the study. We thank the Editor and Reviewer for their time and constructive feedback.

Sincerely,

Soriba Camara, PhD Candidate (SC)
(on behalf of all authors)

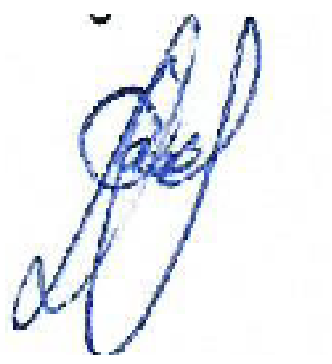

Supplement: Supplementary file 1 [file pgph.0005461.s011.pdf]
