# Supplementary material for: RE-AIM evaluation of the national scale-up of community-based index case HIV testing in Guinea: Successes and challenges
Source: PLOS Glob Public Health. 2026 Nov 10;6(11):e0005461. doi: 10.1371/journal.pgph.0005461 (PMC13750574; doi:10.1371/journal.pgph.0005461)

Response to Editor Comments

Manuscript: Evaluation of community-based HIV index case testing using the RE-AIM framework in Guinea
Journal: *PLOS Global Public Health*

Dear Editor,

We thank you for the careful evaluation of our manuscript and for the detailed and constructive comments provided. These suggestions have helped us substantially improve the clarity, methodological transparency, and interpretation of the study.

In response to the comments, the manuscript has been thoroughly revised. The main revisions include:

- clarification of the study design and analytical framework,
- improved alignment between Methods and Results sections,
- clearer operationalization of RE-AIM dimensions,
- additional methodological explanations for statistical analyses,
- revision of interpretations to avoid causal overstatement,
- improved description of qualitative methods and triangulation, and
- refinement of policy implications to ensure they are directly supported by the empirical findings.

All changes have been incorporated into the revised manuscript. A detailed point-by-point response is provided below.

Point-by-point responses

Lines 42–43

The sentence in the Abstract has been revised to avoid implying that institutionalization is directly demonstrated by the study findings. The text now states that long-term sustainability may depend on stronger institutional integration of community-based organizations alongside improvements in supply chain resilience and financial integration mechanisms.

Lines 44–46

The sentence has been revised to include a concrete recommendation grounded in the evaluation findings. The revised wording highlights practical priorities for programmes expanding community HIV testing, including strengthening supply chain reliability, reinforcing supervision and training of community counsellors, and improving routine data quality systems.

Line 63

The text now explicitly defines index case testing (ICT) as a contact-based HIV testing strategy involving identification and testing of sexual partners and household contacts of individuals diagnosed with HIV.

Line 66

Punctuation has been corrected.

Lines 68–71

The paragraph has been revised to clarify the rationale for evaluating ICT implementation at scale. The revised text now acknowledges that while ICT effectiveness has been documented, evidence on large-scale implementation and long-term integration within national health systems remains limited. References documenting implementation challenges have been added.

Lines 72–73

Absolute numbers have been added. Based on UNAIDS estimates, the figure of 36% of people living with HIV unaware of their status corresponds to approximately **43,200 individuals** in Guinea. The manuscript now explicitly compares this value with the UNAIDS 95-95-95 targets.

Lines 79–80

The rationale for describing ICT as a complex intervention has been clarified, emphasizing its multiple interacting components and dependence on contextual factors such as stigma, disclosure dynamics, supply chains, and coordination between health facilities and community organizations.

Lines 85–87

An appropriate reference supporting the relevance of implementation frameworks in resource-limited settings has been added.

Lines 91–93

The wording has been revised to avoid implying broad regional generalization. The manuscript now emphasizes that findings are context-specific while offering potentially transferable implementation insights.

Lines 97–98

The study design description has been revised to more accurately reflect the analytical structure of the evaluation. The quantitative component combines serial cross-sectional analyses of programme indicators, time-series approaches to assess inter-annual

variability and trends, and longitudinal descriptive analyses to examine organisational continuity of community-based organisations during the implementation period.

Lines 152–153

The description of Phase 3 has been revised for clarity and neutrality.

Line 170

The manuscript now clarifies that geographic expansion was classified under **Adoption**, as it reflects engagement of organisational units (districts and CBOs), whereas **Reach** was defined at the individual level.

Lines 187–190

The Methods section now clarifies that Spearman's rank correlations were used because cascade indicators consisted of aggregated count and proportion data with non-normal distributions. Correlations were calculated using a **region × year panel dataset**.

Lines 191–195

The qualitative analysis is now described as a **hybrid deductive–inductive thematic approach**, with the RE-AIM framework informing the initial coding structure and inductive coding used to identify emergent themes.

Lines 206–208

The manuscript now clarifies that informed consent applied only to qualitative interview participants. Quantitative analyses relied on anonymized aggregated programme data.

Line 230

The term "strong performance" has been removed and replaced with descriptive reporting of indicator values.

Effectiveness indicators (Lines 164–168 vs 230–246)

The Results section has been revised to ensure strict alignment with the predefined effectiveness indicators described in the Methods and S3 Table.

Table 3

Additional methodological detail has been added explaining how correlations were calculated, including the data structure and statistical rationale.

Lines 309–315

The manuscript now clarifies that qualitative findings informed several RE-AIM dimensions. Triangulation was most explicitly presented within the Maintenance

section because qualitative evidence was needed to interpret instability signals identified in quantitative data.

Line 323

The wording has been revised to remove the term “coherent impact,” which could be misleading given the decline observed in some indicators.

Development of themes in Table 4

The manuscript now explains that themes were generated using a hybrid deductive–inductive thematic analysis informed by the RE-AIM framework.

Lines 382–388

The text has been revised to avoid causal interpretation. Observed increases in activity volumes are now described as temporal associations occurring alongside program expansion.

Lines 397–398

The claim that RE-AIM is uncommon in West African research has been removed.

Lines 399–401

The recommendation regarding institutional integration has been explicitly linked to Maintenance findings indicating vulnerabilities in supply chains, financing, and monitoring systems.

Lines 415–417

The manuscript now clarifies that baseline data prior to implementation were not available; therefore the study describes operational performance during the implementation period rather than change relative to baseline.

S3 Table

We confirm that the indicator “median number of testing sessions per CBO per period” was measured using routine monitoring data and used to characterize implementation intensity.

Supplementary materials

All supplementary materials are now explicitly referenced in the manuscript.

Closing statement

We believe these revisions have significantly strengthened the manuscript by improving methodological clarity, analytical consistency, and interpretation of findings. We

appreciate the editor's thoughtful comments and hope the revised manuscript meets the journal's expectations.

Sincerely,
The Authors

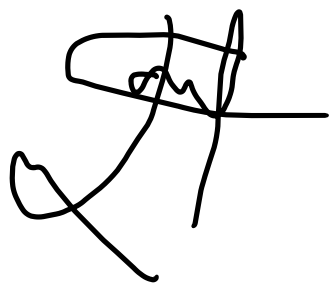

Supplement: Supplementary file 2 [file pgph.0005461.s012.pdf]
