# Supplementary material for: RE-AIM evaluation of the national scale-up of community-based index case HIV testing in Guinea: Successes and challenges
Source: PLOS Glob Public Health. 2026 Nov 10;6(11):e0005461. doi: 10.1371/journal.pgph.0005461 (PMC13750574; doi:10.1371/journal.pgph.0005461)

PLOS Global Public Health

RE-AIM evaluation of the national scale-up of community-based index case HIV testing in Guinea: Successes and Challenges

--Manuscript Draft--

**Manuscript Number:**	PGPH-D-25-03261R3
**Article Type:**	Research Article
**Full Title:**	RE-AIM evaluation of the national scale-up of community-based index case HIV testing in Guinea: Successes and Challenges
**Short Title:**	RE-AIM Evaluation of HIV Index Testing Scale-Up in Guinea
**Corresponding Author:**	Soriba CAMARA, Ph. D studebt<br>Gamal Abdel Nasser University of Conakry: Universite Gamal Abdel Nasser de Conakry<br>Conakry, GUINEA
**Order of Authors:**	Soriba CAMARA, PhD candidate
	Mory 1 Kourouma, PhD candidate
	Castro Gbêmêmali Hounmenou, PhD
	Aly Badara Touré, PhD candidate
	Emile Faya Bongono, PhD candidate
	Sidikiba Sidibé, Associate Professor
	Tamba Mina Millimouno, PhD candidate
	Abdoulaye Sow, PhD
	Alioune Camara, Full Professor
	Alexandre Delamou, Full Professor
**Keywords:**	Keywords: HIV; community-based testing; index case testing; RE-AIM; Guinea.
**Abstract:**	In Guinea, an estimated 36% of people living with HIV remain unaware of their status, hindering progress toward the UNAIDS 95-95-95 targets. This study evaluated the effectiveness, implementation fidelity, and sustainability of the community-based index case testing (ICT) strategy using the RE-AIM framework. We conducted a retrospective mixed-methods evaluation (2021–2024) across 17 districts supported by 26 community-based organizations (CBOs), using the RE-AIM framework (Reach, Effectiveness, Adoption, Implementation, and Maintenance). Quantitative analyses included mixed-effects binomial models, segmented regression, and Spearman correlations. In parallel, 28 semi-structured interviews with key stakeholders were thematically analyzed in NVivo, guided by the five RE-AIM dimensions. Among 50,609 individuals reached, 8,112 underwent HIV testing, yielding a positivity rate of 8.4% (95% CI: 7.8 to 9.0), with 84.9% (95% CI: 81.9 to 87.6) successfully linked to care. Linkage to care was substantially higher among women (93.6%) and children (90.2%) than among men (14.1%). Adoption expanded from 3 to 17 districts between 2021 and 2023, while cascade performance displayed notable inter-district heterogeneity. Qualitative findings identified strong PMTCT mentorship, community trust, and counseling continuity as key facilitators, contrasted by recurrent challenges including commodity stockouts, financial dependency, and intrahousehold disclosure barriers. The ICT strategy was associated with expanding testing uptake and high linkage to care levels under routine programmatic conditions. Maintenance findings suggest that strengthening institutional integration of CBOs, reinforcing supply chain stability, and improving financing continuity may enhance programme sustainability. These findings provide context-sensitive operational evidence that may inform the adaptation of differentiated community-based testing strategies in comparable implementation settings.
**Additional Information:**	

Powered by Editorial Manager® and ProduXion Manager® from Aries Systems Corporation

Question	Response
**Financial Disclosure**<br><br>Enter a financial disclosure statement that describes the sources of funding for the work included in this submission and the role the funder(s) played. This includes grants and any commercial funding of the work or authors.<br><br>This statement will be typeset if the manuscript is accepted for publication.<br><br>*Please review the submission guidelines and the instructions link below for detailed requirements and guidance.*	This work was supported by the WHO Special Programme for Research and Training in Tropical Diseases (TDR) through its support programme for early-career researchers in Francophone Africa (Grant No. V1 20170329 to SC). The award was administered through Cheikh Anta Diop University, Dakar, Senegal, and the Africa Centre of Excellence at Université Gamal Abdel Nasser de Conakry, Guinea. The funders had no role in study design, data collection and analysis, decision to publish, or preparation of the manuscript.
**Competing Interests**<br><br>On behalf of all authors, disclose any competing interests that could be perceived to bias this work.<br><br>This statement will be typeset if the manuscript is accepted for publication.<br><br>*Please review the instructions link below and PLOS Global Public Health's competing interests policy to determine what information must be disclosed at submission.*	The authors have declared that no competing interests exist.
**Data Availability**<br><br>Before publication, Authors are required to make fully available and without restriction all data underlying their findings. Sharing the data underlying your findings increases the visibility, credibility, and impact of your work, while supporting transparency and future scientific discovery. Please see our PLOS Data Policy page for detailed information on this policy.	All data underlying the findings of this study are provided as Supporting Information. The quantitative analyses were conducted using a de-identified aggregated dataset of quarterly programme monitoring data, included as S8 Data. No individual-level identifiers are included in the shared dataset. These data are sufficient to reproduce the analyses reported in the manuscript. Data sharing is consistent with the ethical approval granted by the National Ethics Committee for Health Research in Guinea and complies with applicable confidentiality requirements.

A **Data Availability Statement**, detailing where the data can be accessed, is required at first submission. Insert your Data Availability Statement in the box below.<br><br>Please see the data reporting section of our submission guidelines for instructions on what you need to include in your Data Availability Statement. Examples of acceptable Data Availability Statements are included here for reference:<br>1. The minimal data set [and accompanying code, where generated] is available at [repository name] via [DOI hyperlink]<br>2. No data was generated by this study. The following existing data sources were used: [dataset name] from [dataset location] available via [web address or DOI hyperlink].<br>3. For Study Protocols: No datasets were generated or analysed during the current study. All relevant data from this study will be made available upon study completion.<br><br>*PLOS allows rare exemptions to address legal and ethical concerns. If you have legal or ethical restrictions, please use the box below to detail these in full sentences for the Journal team to consider.*<br><br>This statement will be typeset if the manuscript is accepted for publication.	

Powered by Editorial Manager® and ProduXion Manager® from Aries Systems Corporation

Manuscript Click here to access/download;Manuscript;Manuscript3.docx

RE-AIM evaluation of the national scale-up of community-based index case HIV testing in Guinea: Successes and Challenges

Soriba Camara*[1], Mory 1 Kourouma[1,2], Castro Gbêmêmali Hounmenou [2,3], Aly Badara Touré[1,4], Emile Faya Bongono [2,5]; Sidikiba Sidibé [1], Tamba Mina Millimouno[1,4], Abdoulaye Sow[1], Alioune Camara [1], Alexandre Delamou [1,4].

[1] Department of Public Health, Faculty of Health Sciences and Techniques, Gamal Abdel Nasser University of Conakry, Guinea

[2] Guinea Infectious Disease Research and Training Center, Gamal Abdel Nasser University of Conakry

[3] University of Labé, Department of Computer Science, Labé, Guinea

[4] Africa Center of Excellence for Prevention and Control of Communicable Diseases, Gamal Abdel Nasser University of Conakry, Guinea

[5] Department of Veterinary Medicine, Chair of Infectious and Parasitic Diseases, Higher Institute of Science and Veterinary Medicine of Dalaba, Guinea.

***Corresponding author:** Soriba Camara, Email: soribasante@gmail.com, (SC).

ORCID: https://orcid.org/0009-0009-8951-078X

Abstract

In Guinea, an estimated 36% of people living with HIV remain unaware of their status, hindering progress toward the UNAIDS 95-95-95 targets. This study evaluated the effectiveness, implementation fidelity, and sustainability of the community-based index case testing (ICT) strategy using the RE-AIM framework. We conducted a retrospective mixed-methods evaluation (2021–2024) across 17 districts supported by 26 community-based organizations (CBOs), using the RE-AIM framework (Reach, Effectiveness, Adoption, Implementation, and Maintenance). Quantitative analyses included mixed-effects binomial models, segmented regression, and Spearman correlations. In parallel, 28 semi-structured interviews with key stakeholders were thematically analyzed in NVivo, guided by the five RE-AIM dimensions. Among 50,609 individuals reached, 8,112 underwent HIV testing, yielding a positivity rate of 8.4% (95% CI: 7.8 to 9.0), with 84.9% (95% CI: 81.9 to 87.6) successfully linked to care. Linkage to care was substantially higher among women (93.6%) and children (90.2%) than among men (14.1%). Adoption expanded from 3 to 17 districts between 2021 and 2023, while cascade performance displayed notable inter-district heterogeneity. Qualitative findings identified strong PMTCT mentorship, community trust, and counseling continuity as key facilitators, contrasted by recurrent challenges including commodity stockouts, financial dependency, and intrahousehold disclosure barriers. The ICT strategy was associated with expanding testing uptake and high linkage to care levels under routine programmatic conditions. Maintenance findings suggest that strengthening institutional integration of CBOs, reinforcing supply chain stability, and improving financing continuity may enhance programme sustainability. These findings provide context-sensitive operational evidence that may inform the adaptation of

differentiated community-based testing strategies in comparable implementation settings.

Keywords: HIV, community-based testing, index case testing, RE-AIM; Guinea.

Introduction

HIV testing remains a cornerstone of the global response, as it determines both the initiation of antiretroviral therapy and the interruption of transmission chains [1] . In 2022, an estimated 39 million people were living with HIV worldwide, with a substantial proportion still unaware of their serostatus, jeopardizing progress toward the UNAIDS 95-95-95 targets set for 2030 [2–5]. Sub-Saharan Africa accounts for nearly two-thirds of this burden and continues to face structural and sociocultural barriers that constrain universal access to testing [2,6] .

In West Africa, diagnostic coverage remains particularly low in rural areas and among young populations at risk [7–12]. To address this gap, community-based approaches have emerged as differentiated and complementary strategies to conventional health services. Among these, index case testing (ICT), involves systematic identification and testing of sexual and household contacts of persons diagnosed with HIV, aiming to improve case detection efficiency and early linkage to care [8,13–18] . Although ICT has demonstrated effectiveness in improving HIV case detection and linkage to care [19–21], evidence on its large-scale implementation and long-term integration within national health systems remains comparatively limited, particularly in low-resource settings [22–25] . Prior studies have documented important operational and contextual challenges, including stigma-related barriers, variability in programme performance, workforce constraints, and supply chain disruptions [26–28]. Understanding ICT performance under routine programme conditions is therefore essential [29,30].In Guinea, national HIV prevalence was estimated at 1.5% in 2018, yet approximately 36% of people living with HIV remain unaware of their status (approximately 43,200 individuals) [31]. Since 2019, the National HIV and Hepatitis Control Program (PNLSH) has piloted an innovative ICT strategy anchored in prevention of mother-to-child transmission

(PMTCT) sites and implemented through community-based organizations (CBOs). Despite its promise, ICT has remained marginal in practice, representing only a small fraction of tests performed outside PMTCT services [32].

Evaluating complex interventions requires frameworks that capture both clinical effects and the conditions under which implementation occurs. ICT qualifies as a complex intervention because it involves multiple interdependent components delivered across facility and community levels, including index-case identification, contact elicitation, community sensitization, HIV testing, referral, and linkage to care. In line with the Medical Research Council guidance on complex interventions, ICT outcomes are influenced not only by these components but also by contextual factors such as stigma, disclosure dynamics, supply-chain reliability, and organizational capacity [33–36] .The RE-AIM framework (Reach, Effectiveness, Adoption, Implementation, Maintenance), widely applied in public health, offers a structured approach to assess population coverage, behavioral and clinical outcomes, organizational uptake, fidelity of implementation, and sustainability [37]. This multidimensional lens is particularly relevant in resource-limited settings, where the success of an intervention depends not only on its efficacy but also on its feasibility, scalability, and institutional anchoring [22–25,34].

Against this background, the present study applied the RE-AIM framework to evaluate an index case HIV testing program implemented in Guinea. The objective was to generate robust evidence on its effectiveness, adoption, and implementation processes, while identifying contextual factors influencing sustainability. Findings may provide insights relevant to comparable francophone West African settings.

Methods

Study Design

This study used a mixed-methods implementation evaluation design based on routine programme monitoring data collected between 2021 and 2024. The quantitative component combined serial cross-sectional analyses of programme indicators, time-series approaches to assess inter-annual variability and trends, and longitudinal descriptive analyses to examine organizational continuity of CBOs during the implementation period. Quantitative data were analyzed from April 20 to May 18, 2025, while qualitative interviews and document reviews were conducted between December 16, 2023, and January 5, 2024. The evaluation focused on a community-based HIV ICT programme implemented in Guinea between 2021 and 2024, under the study protocol approved by the National Ethics Committee for Health Research (CNERS, Ref. No. 060/CNERS/23).

The implementation teams consisted of staff from the National HIV and Hepatitis Control Programme (PNLSH), Fraternité Médicale Guinée (FMG) as Global Fund sub-recipient, and community-based organizations (CBOs) and PMTCT sites delivering ICT activities. These actors were responsible for program planning, service delivery, and routine reporting. In contrast, data collection and analysis were conducted by an independent research team based at the University Gamal Abdel Nasser of Conakry, who had no operational responsibility in program implementation. These researchers designed the evaluation protocol, developed data collection tools, conducted interviews, and performed quantitative and qualitative analyses. Implementers did not participate in statistical modelling or interpretation of the findings.

The RE-AIM framework (Reach, Effectiveness, Adoption, Implementation, Maintenance) guided the evaluation, offering a multidimensional lens to assess both clinical effectiveness and implementation fidelity [37].

Study Population and Sites

The study was conducted in the 17 districts in which the community-based index case testing (ICT) program had been formally implemented during the study period. These districts were not purposively selected; rather, they reflected the progressive national rollout of the ICT strategy, which began with a pilot phase in three districts (Boké, Siguiri and N'Zérékoré) in 2021 and was subsequently expanded to 17 districts by 2023 under the coordination of the National HIV and Hepatitis Control Programme. District inclusion was therefore determined by programme implementation status, the presence of PMTCT sites involved in index-case identification, and the operation of designated CBOs within the Global Fund supported platform (S1 Fig)**.**

The target population consisted of HIV-positive women followed in PMTCT services in these districts and their sexual and household contacts identified through the ICT. Eligible participants for qualitative interviews were adults aged 18 years or older who had direct experience with the community HIV testing strategy, either as beneficiaries or implementers. Children and adolescents were not recruited for the qualitative component of the study. The organizational units comprised of 26 community-based organizations (CBOs) operating in the participating districts. Participants were approached through CBO focal points and invited individually after being informed about the study's objectives and confidentiality procedures. Interviews were conducted in French or local languages (Susu, Malinké, Fula, Kpelle, and Guerzé), depending on participant preference. Exclusion criteria included individuals residing outside

intervention areas, those not exposed to the community-based strategy, and those who did not provide informed consent.

Data Sources and Collection

Four complementary data sources were mobilized. Programmatic data consolidated from monthly reports served as the primary basis for quantitative analysis. Routine programmatic data were produced within the national HIV program and reported by community-based organizations (CBOs) to the Global Fund sub-recipient Fraternité Médicale Guinée. These aggregated datasets included cascade indicators such as index cases identified in PMTCT sites, contacts elicited, numbers referred and tested for HIV, HIV-positive diagnoses, linkage to care, and supervision activities. These reports were completed by CBO focal persons and validated by district supervisors. In contrast, KoboCollect was used exclusively for research extraction of individual-level variables not available in routine reports, including age, sex, relationship to the index case, acceptance or refusal of HIV testing and reasons provided, place of testing, linkage outcomes, and reported operational barriers such as commodity stock-outs. KoboCollect forms were completed by trained research assistants independent from implementation teams. Programmatic documents (protocols, guidelines, supervision reports) were also reviewed to contextualize implementation processes and support data triangulation. Children aged <15 years were represented only in the routine programme records used for secondary quantitative analysis. They were not contacted, recruited, or interviewed by the research team, and no research-specific procedures were conducted with them. The quantitative dataset contained de-identified routine programme information, including age group, sex, HIV testing, and linkage indicators.

Semi-structured interviews were conducted with 28 purposively selected participants representing key actor groups involved in or affected by the intervention: (i) national-level program coordinators (PNLSH and Fraternité Médicale Guinée), (ii) health-facility personnel from PMTCT sites, (iii) staff of CBOs (coordinators, supervisors and lay counsellors), and (iv) index cases and household members. These categories contributed respectively to policy-level perspectives, facility-community interface, operational delivery in communities, and user experience and acceptability. All individuals participating in interviews were recruited specifically for research purposes and provided written informed consent prior to data collection. Recruitment continued until thematic saturation was reached. Quantitative analyses relied on de-identified aggregated programmatic data routinely collected within the national HIV programme. Individuals who reached through community awareness, counselling, and testing activities were beneficiaries of routine service delivery and were not enrolled as research participants. Consequently, individual informed consent was not required for secondary analysis of anonymized routine data, consistent with the ethical approval obtained. All programmatic and interview data were anonymized prior to analysis, with no identifying information retained. Data were securely stored on password-protected devices accessible only to the research team. Signed consent forms are archived at the principal investigator's institution. Interviews conducted in local languages were transcribed and translated into French before coding and analysis.

Description of the Intervention

The intervention was implemented in three phases (Boké, Siguri and N'Zérékoré), reflecting a progressive scale-up of community-based HIV index case testing in Guinea.

Phase 1 (2021) launched the pilot with five CBOs across three districts, focusing on establishing operational protocols, building initial capacity, and testing feasibility. At this

stage, PMTCT sites identified index cases from clinical registers and shared lists of partners and household contacts with CBOs. These contacts were approached for sensitization and HIV testing, conducted either at home or in community settings. Individuals who tested positive were referred to PMTCT sites or designated hospitals for confirmatory testing and treatment initiation. **Phase 2 (2022) Adjustment** to 26 CBOs across 17 districts, with emphasis on training and operational rollout. Fraternité Médicale Guinée (FMG), the national sub-recipient of Global Fund grants, coordinated capacity-building, supervision, and resource management. PMTCT sites ensured clinical quality assurance, while CBOs provided community-based continuity through psychosocial support, appointment reminders, and follow-up visits.

Phase 3 (from 2023 onward): corresponded to the geographic expansion and operational consolidation of the intervention. During this phase, the number of participating districts and CBOs increased substantially, while programme activities continued under routine implementation conditions. Referral, testing, and follow-up mechanisms remained anchored within the established PMTCT– community interface (S2 Fig).

CBOs delivered a standardized psychosocial support package, including post-test counselling (emotional support and stigma coping), assistance with voluntary disclosure of HIV status when requested, counselling on ART initiation and adherence, accompaniment to PMTCT/ART services, and linkage to peer and community support groups. Appointment reminders were provided through a dual mechanism combining telephone calls/SMS where feasible and home visits when clients could not be contacted by phone. Reminders were triggered by missed appointments recorded in PMTCT registers according to national follow-up schedules (R1 = one month, R3 = three months, R6 = six months), with reminder actions initiated within seven days after a

missed visit. Follow-up visits were conducted monthly as part of routine support and more frequently for clients at risk of loss to follow-up, those who had not disclosed their HIV status, or newly diagnosed household contacts. Visits occurred either at home, particularly when confidentiality, disclosure support, or household testing were needed, or in clinics during scheduled PMTCT/ART appointments. Priority subgroups included: (i) women lost or at risk of loss to follow-up, (ii) partners not yet tested, (iii) HIV-positive contacts not yet on ART, and (iv) women requesting assistance with disclosure.

Operationalization of the RE-AIM Framework

The RE-AIM framework guided the evaluation by defining operational indicators for each of its five dimensions and specifying the variables analyzed.

Reach was measured by the total number of individuals reached through awareness activities, those who received post-identification counseling, those referred to HIV testing services, and those who accepted testing. Sociodemographic characteristics (age, sex) were used as explanatory variables to describe coverage and beneficiary profiles.

Effectiveness was assessed using analytically distinct conversion and outcome indicators across the HIV testing and care cascade. These included: (i) referral for HIV testing among counselled individuals; (ii) HIV test uptake among those referred for testing; (iii) linkage to care among individuals identified as HIV-positive; and (iv) ART initiation among those referred or linked to care. Whereas Reach captured the absolute number of individuals progressing through the pre-diagnosis cascade, Effectiveness reflected the corresponding conversion proportions and downstream care outcomes. The subgroup analysis presented in Fig 2 specifically examined linkage to care among HIV-positive individuals by age-sex category. **Adoption** was evaluated by the number and proportion of districts and CBOs that implemented the intervention. Geographic

expansion was classified under the Adoption dimension because it reflected the progressive engagement of organizational implementation units (districts and CBOs). Reach indicators were defined at the individual level, capturing the population effectively exposed to intervention activities.

Implementation indicators included intensity (median number of HIV testing sessions per CBO per quarter), inter-annual stability (coefficients of variation for key indicators), and quality (treatment initiation rate), as well as correlations across cascade steps. Correlation analyses were conducted using aggregated cascade indicators structured as a region × year panel dataset. Analyses examined the temporal distribution of sessions, inter-CBO performance, and organizational adaptations. **Maintenance** was assessed through the number of CBOs remaining active at the end of the study period, completeness of the reporting system, and detection of supply chain interruptions. Variables included funding trends, institutional integration, and established monitoring and supervision mechanisms (S3 Table).

Data Analysis

Quantitative analyses were conducted at two complementary levels of observation. Because routine monitoring data were recorded at multiple operational levels, different units of analysis were used depending on the analytical objective: individual-level observations for regression models, CBO × year aggregates for organizational performance analyses, and region × year aggregates for cascade correlation analyses. Individual-level analyses were performed to assess determinants of HIV test acceptance, referral, and treatment initiation. Mixed-effects binomial logistic regression models were applied to account for the hierarchical data structure (individuals nested within community-based organizations, CBOs). Fixed effects included sex, age group, calendar year, and intervention intensity, while a random intercept at the CBO level

accounted for clustering [43]. For the subgroup analysis presented in Fig 2, a separate grouped binomial mixed-effects logistic regression model was fitted to estimate linkage to care among individuals recorded as HIV-positive. The outcome was specified as the number linked to care versus the number not linked among HIV-positive individuals. Age-sex category was included as a fixed effect, and a random intercept at the CBO level accounted for clustering. Four observations in which the recorded number linked to care exceeded the number of HIV-positive individuals were excluded as internally inconsistent. Marginal predicted probabilities and 95% confidence intervals were derived from the fitted model using estimated marginal means. Organizational-level analyses were conducted using aggregated programmatic indicators. For longitudinal analyses of referral and linkage to care outcomes, the unit of analysis was the aggregated CBO × year observation. Repeated annual measurements were modelled using mixed-effects regression. Marginal predicted probabilities were derived from the fitted models as estimated marginal means, representing adjusted model-based probabilities rather than crude observed proportions. Ninety-five percent confidence intervals (95% CI) were calculated using the model-based standard errors. Model diagnostics and technical outputs (AIC, BIC, inter-CBO variance and dispersion parameters) are presented in the Results section and Supporting Information (S4). Associations between cascade activity volumes were assessed using Spearman's rank correlation coefficients [38–40]. The analyses were conducted on aggregated programmatic indicators derived from routine monitoring data and structured as a region × year panel dataset (2021–2024). Variables consisted of aggregated count and proportion measures for sensitization, counseling, testing, HIV positivity, and linkage to care. Given the skewed, non-normal distribution and heteroscedasticity of these indicators, Spearman's nonparametric method was selected. The unit of analysis was

the aggregated region × year observation. Correlation strength was interpreted according to predefined thresholds (Table 3). Implementation intensity was operationalized as the median number of testing sessions conducted per CBO per reporting period, calculated from routine monitoring data (S6 Table). Mean values presented in summary tables (Tables 1 and 2) are provided for descriptive purposes only due to the skewed distribution of activity volumes. All analyses were performed in R version 4.3.1.

Qualitative analysis followed a hybrid deductive-inductive thematic approach supported by NVivo [41]. The RE-AIM framework informed the initial coding structure (deductive component), ensuring conceptual alignment with evaluation dimensions. Within each RE-AIM domain, inductive coding was applied to identify context-specific subthemes and operational factors emerging from participant narratives [42,43], The coding framework was iteratively refined through independent double coding and consensus meetings, enhancing analytic reliability (S5 Table). Themes synthesized in Table 4 reflect this structured analytic process.

Integration of quantitative and qualitative strands followed an explanatory sequential design: quantitative findings guided the focus of interviews and interpretation, enabling exploration of perceived causes of observed trends (e.g., supply shortages, stigma, organizational bottlenecks).

Ethical Considerations

The study protocol, including the secondary analysis of de-identified routine programme data, was approved by the National Ethics Committee for Health Research (CNERS, Ref. No. 060/CNERS/23). The study did not involve any clinical or interventional component but consisted of cross-sectional surveys and semi-structured interviews with participants who had experience with the community HIV testing

approach**.** Written informed consent applied exclusively to participants recruited for the qualitative interviews. All interview participants were adults aged 18 years or older; no children or adolescents were recruited for interviews or any other research-specific data collection. All participants were informed about the study objectives, the voluntary nature of their participation, and the confidentiality measures in place. Children aged <15 years were included only through de-identified routine programme records used for secondary quantitative analysis. They were beneficiaries of standard HIV programme services and were not enrolled or contacted as research participants. Consequently, no additional research-specific parental permission or child assent was sought for the secondary analysis of these records, as covered by the CNERS-approved study protocol. Any consent procedures associated with routine HIV testing and care were governed by national programme procedures and were separate from consent for participation in this research.

For adult interview participants who were unable to read, the information sheet was read aloud in their preferred language and was documented in the presence of an independent witness. All datasets were de-identified before analysis, and no personal identifiers were retained.

Results

Reach

A total of 50,609 individuals were reached through community awareness and mobilization activities across the intervention districts. Among them, 26,025 (51.4%, 95% CI: 51.0 to 51.9) received individualized post-identification risk counseling. Of those counseled, 14,169 (54.4%, 95% CI: 53.8 to 55.0) were referred for HIV testing. Among individuals referred, 8,112 (57.3%, 95% CI: 56.3 to 58.3) completed an HIV test. The overall HIV positivity rate among those tested was 8.4% (95% CI: 7.8 to 9.0). Of individuals diagnosed with HIV, 577 (84.9%, 95% CI: 81.9 to 87.6) were successfully linked to HIV care and treatment services. Progression along the cascade is summarized in Fig 1.

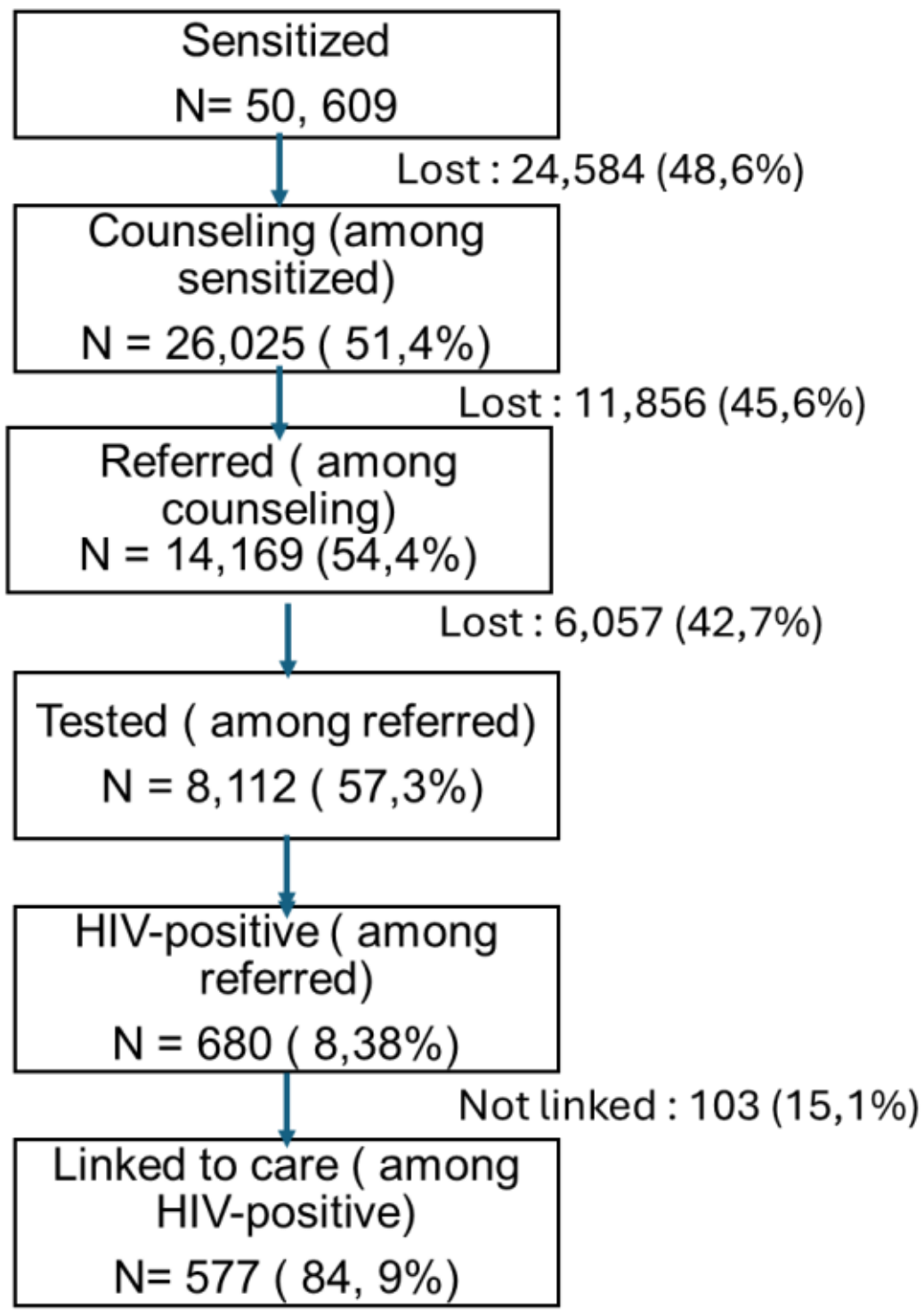

Fig 1: Community-based HIV testing cascade in Guinea, 2021–2024.
Progression of individuals from community outreach to HIV testing and linkage to care, showing cumulative attrition across cascade stages and the proportion linked to care after HIV diagnosis.

Effectiveness

Effectiveness was assessed using four analytically distinct cascade indicators: testing referral among counselled individuals, test uptake among those referred for testing, linkage to care among individuals diagnosed HIV-positive, and ART initiation among those referred or linked to care. These indicators were analysed across implementation phases (Table 1 and Fig 2). Across phases, variation was observed for all three indicators. In 2021 (The launch phase), 53.2% of counseled individuals were referred for HIV testing, 90.2% of those referred completed testing (test acceptance) and 73.3% of individuals referred to care initiated ART. In 2022 (adjustment phase), the proportion of counseled individuals referred for testing declined to 46.7%, and test acceptance among referred individuals decreased to 83.7%. In contrast, ART initiation among those referred increased to 94.3%. The expansion phase (2023–2024) showed heterogeneous patterns. The test uptake value recorded for 2023 (> 1,000%) was identified as a non-interpretable value caused by a denominator reporting error in routine program data, this value was excluded from summary analyses (Table 1). In 2023, the referral rate among counseled individuals dropped to 3.6%, coinciding with geographic expansion from 3 to 17 districts and from 5 to 26 CBOs. During the same year, routine monitoring data showed increased inter-annual variability in cascade indicators and a discrepancy between recorded referrals and tests performed in a subset of CBOs, as further described under the Maintenance dimension. In 2024, referral increased to 75.4% and test acceptance reached 62.6%, and ART initiation stabilized at 76.4%.

A separate subgroup analysis examined linkage to care among individuals identified as HIV-positive. The adjusted probability of linkage was 93.6% among women (95% CI: 88.8 to 96.5; n = 389), 90.2% among children aged <15 years (95% CI: 80.2 to 95.5; n

= 238), and 14.1% among men (95% CI: 7.1 to 26.1; n = 109). The mixed-effects binomial model accounting for clustering at the CBO level showed markedly lower linkage to care among men (Fig 2). These values represent model-based marginal predicted probabilities rather than crude proportions. The subgroup sample sizes correspond to the total numbers of HIV-positive individuals represented in the grouped binomial observations retained by the fitted model.

Adoption and Maintenance dimensions provide complementary context to interpret effectiveness patterns. Adoption describes the scale-up of organizational and geographic coverage, while Maintenance assesses the continuity and stability of implementation conditions influencing cascade progression.

Table 1: Effectiveness indicators of community-based HIV index testing across implementation phases in Guinea (2021–2024)

Phase	Mean testing referral rate	Mean test uptake	Mean ART Initiation
Launch (2021)	53.2%	90.2%	73.3%
Adjustment (2022)	46.7%	83.7%	94.3%
Expansion (2023)	3.6%	NA†	74.3%
(2024)	75.4%	62.6%	76.4%

NA† 2023 test uptake value excluded due to a denominator reporting error producing a non-interpretable rate >1000%. Testing-referral rate was calculated among counselled individuals; test uptake was calculated among individuals referred for HIV testing. These indicators are distinct from the post-diagnosis referral/linkage outcome presented in Fig 2.

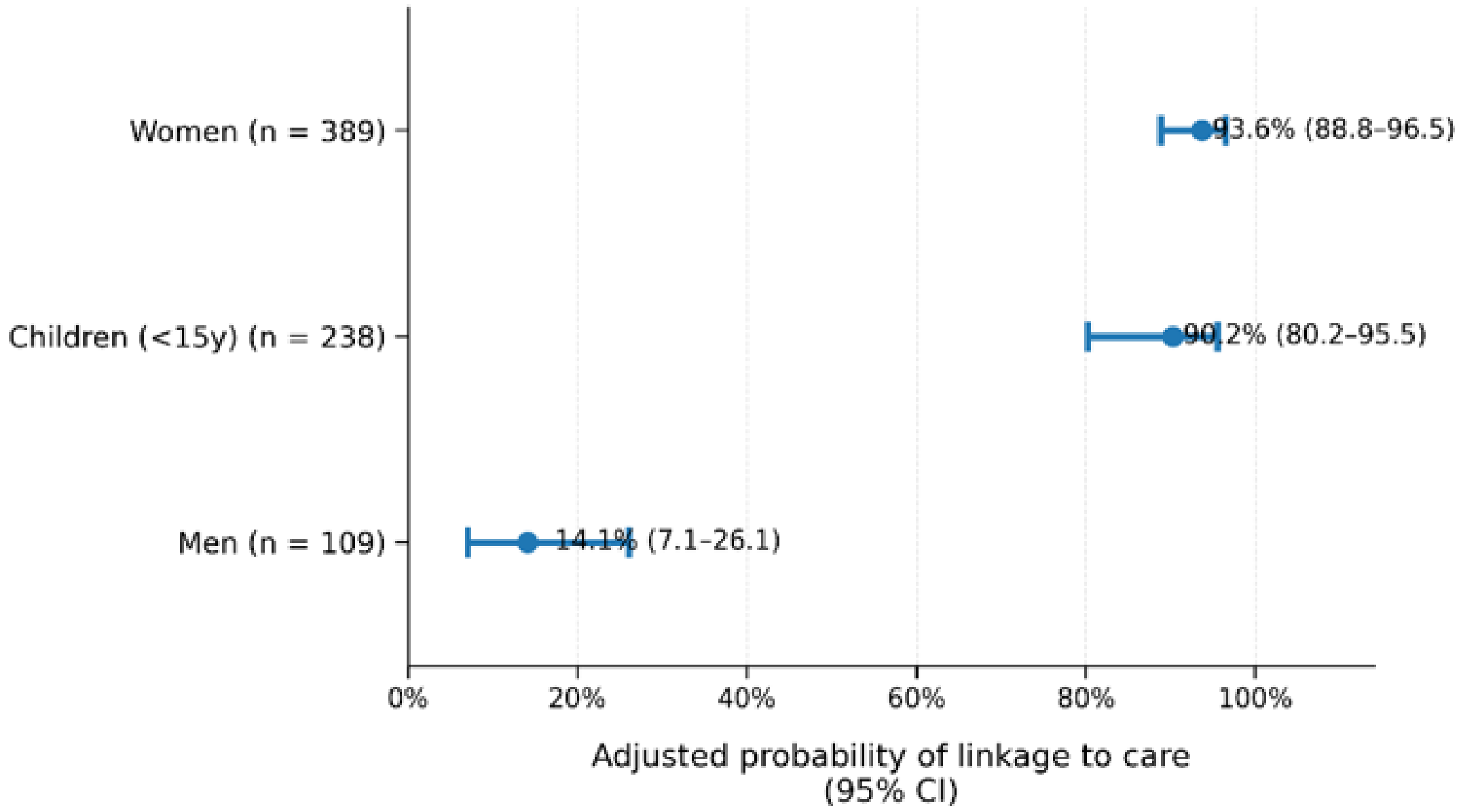

Fig 2 : **Marginal predicted probabilities of linkage to care among HIV-positive individuals by age group and sex. Adjusted probabilities were derived from a mixed-effects binomial logistic regression model with age-sex category as a fixed effect and a random intercept at the CBO level. Sample sizes represent the numbers of HIV-positive individuals contributing to the fitted model: women, n = 389; children aged <15 years, n = 238; and men, n = 109. Points represent marginal predicted probabilities, and horizontal bars represent 95% confidence intervals.**

Adoption

The intervention was initially implemented in three districts and five community-based organizations (CBOs) in 2021 and was progressively expanded to 17 districts and 26 CBOs by 2023. This scaling-up corresponded to a 340% increase in the number of participating districts and a 420% increase in the number of implementing CBOs compared with the 2021 baseline.

At the planning stage in 2020, 26 CBOs across 17 districts had been formally designated as eligible implementers by the national program. By 2023, all 26 eligible CBOs were implementing the intervention, corresponding to 100% organizational adoption among eligible sites. The 340% and 420% increases therefore indicate relative program expansion over time, whereas the 100% value reflects complete adoption among those

officially designated as eligible. No eligible CBOs or districts declined participation. Reporting adoption indicators was high, with 98.7% data completeness over the study period (Fig 3).

Longitudinal changes in referral and linkage indicators were assessed using mixed-effects regression models, with calendar year entered as a time variable and a random intercept at the CBO level to account for repeated measurements within organizations. This analysis showed a statistically significant average annual decline in referral and linkage to care, estimated at −2.54% per year (95% CI: −4.86 to −0.21; p = 0.033).

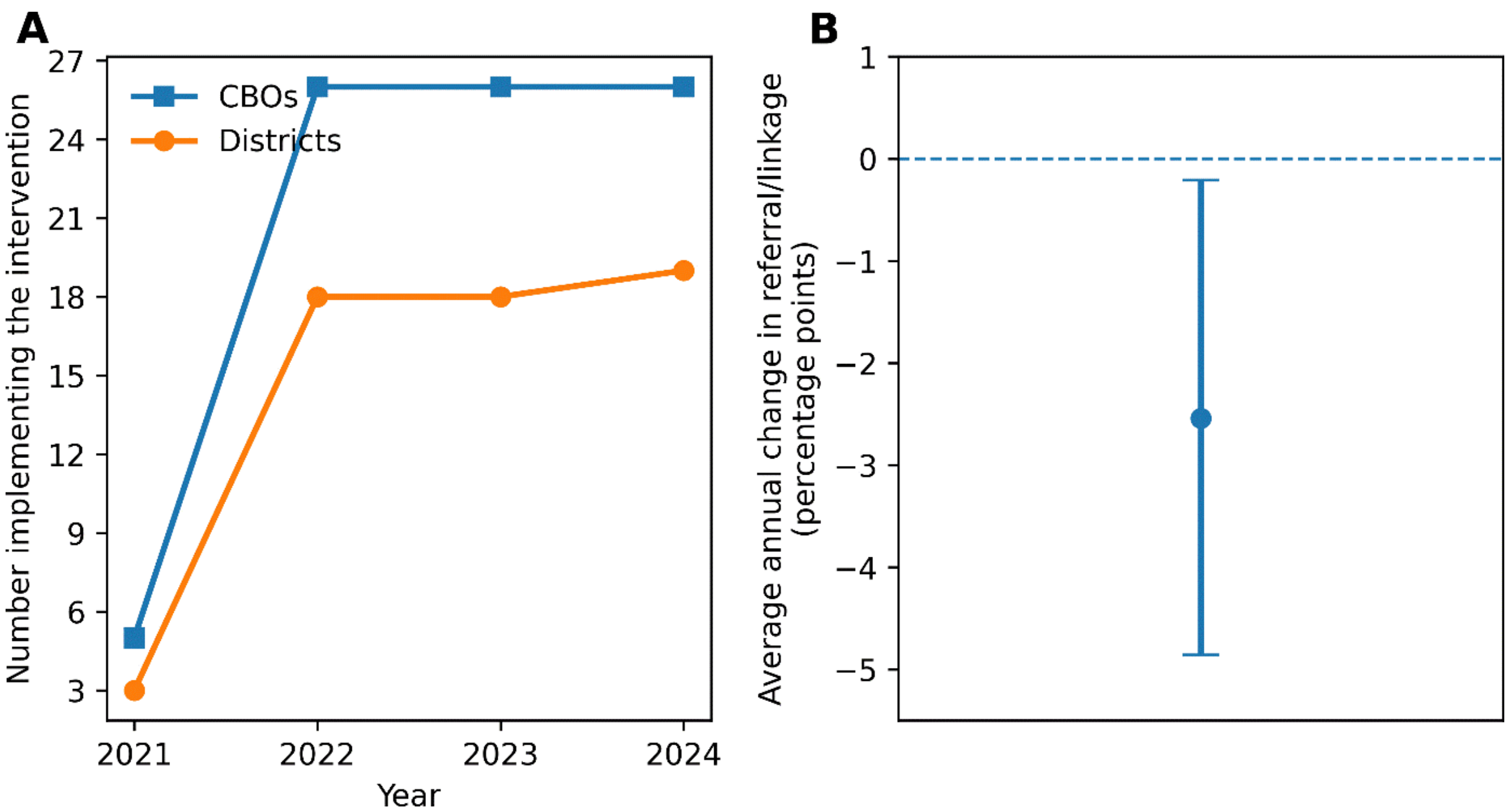

Fig 3 :. Adoption and longitudinal change in referral/linkage indicators. (A) Scale-up of community-based index testing, showing the number of districts and CBOs implementing the intervention (2021–2023). (B) Average annual change in referral/linkage estimated from mixed-effects regression with calendar year as time variable and a CBO-level random intercept; points show model-based estimates and bars 95% confidence intervals (percentage-point change).

Implementation

The implementation unfolded in three operational phases (Table 4, S6 Table): an initial high-intensity phase in 2021, characterized by rapid mobilization and high activity volumes; an adjustment phase in 2022, marked by stabilization of procedures; and an

expansion phase in 2023, corresponding to the geographic scale-up of community-based organizations (CBOs).

Interannual stability analysis showed substantial variability in upstream cascade indicators, including sensitization (CV = 0.63), counseling (CV = 0.63), and testing (CV = 0.58). Downstream indicators such as HIV positivity (CV = 1.82) and linkage to care (CV = 1.87) exhibited markedly higher variability (Table 2). Correlation analyses, performed on cascade indicators structured as a region × year panel dataset, showed very strong monotonic associations between sensitization, counseling, and testing ($\rho > 0.90$, $p < 0.001$). The correlation between HIV-positive cases and linkage to care was also very strong ($\rho = 0.946$, $p < 0.001$). Moderate correlations were observed between counseling and linkage to care ($\rho = 0.561$, $p = 0.029$) and between testing and linkage to care ($\rho = 0.542$, $p = 0.037$), while other associations were weaker and not statistically significant (Table 3).

Table 2: Interannual stability of HIV testing cascade indicators in the community-based programme, Guinea (2021-2024)

Indicator	N° years	Annual mean	Annual SD	CV (2021–2024)	Interpretation
Counseling	4	542.0	341.0	0.63	High variability
Linked to care	4	20.8	38.9	1.87	Very high variability
HIV positive	4	21.5	39.1	1.82	Very high variability
Sensitized	4	531.0	341.0	0.64	High variability
Tested	4	490.0	282.0	0.58	High variability

CV = coefficient of variation (SD/mean). Interpretive thresholds: <0.20 = low; 0.20–0.50 = moderate; 0.50–1.00 = high; >1.00 = very high.

Table 3: Spearman correlation coefficients (ρ) between HIV testing cascade indicators (region × year panel, 2021–2024)

Cascade relationship (Variable 1 → Variable 2)	ρ (Spearman)	*p*-value	Strength	Expected programmatic linkage
Awareness → Counseling	0.929	<0.001	Very strong	Conversion from sensitization to counseling
Counseling → Testing	0.945	<0.001	Very strong	Expected stepwise progression
Awareness → Testing	0.917	<0.001	Very strong	Overall upstream coherence
HIV positive → Linked to care	0.946	<0.001	Very strong	Post-diagnosis referral mechanism
Awareness → Linked to care	0.592	0.020	Moderate	Indirect association
Counseling → Linked to care	0.561	0.029	Moderate	Indirect linkage via testing
Testing → Linked to care	0.542	0.037	Moderate	Reflects conversion to treatment
Awareness → HIV positive	0.492	0.063	Weak–moderate	Not significant
Counseling → HIV positive	0.466	0.080	Weak–moderate	Not significant
Testing → HIV positive	0.495	0.061	Weak–moderate	Not significant

Spearman correlation coefficient. Interpretive thresholds: < 0.20 = very weak; 0.20–0.39 = weak; 0.40–0.59 = moderate; 0.60–0.79 = strong; ≥ 0.80 = very strong. Statistical significance set at $p < 0.05$.

Maintenance

Between 2022 and 2024 the community-based network remained stable, with 26 active CBOs and a median operational tenure of three years. Programmatic records did not directly document the availability of testing commodities at the CBO level. Nevertheless, several indirect signals of operational instability were detected. Each year since 2022, approximately 19.2% of CBOs showed a discrepancy between the number of recorded referrals and the number of tests performed. Likewise, 7.7% to 11.5% of CBOs exhibited atypical intra-annual fluctuations in the tested-to-referred ratio. Taken together, these findings indicate that “maintenance” was achieved in terms of organizational presence

and program footprint, but not uniformly in terms of routine operational performance. These quantitative patterns were further contextualized by qualitative findings, which also informed other RE-AIM dimensions (Reach, Effectiveness, and Implementation), but were particularly critical for interpreting sustainability-related instability signals. Multiple participants reported structural and organizational constraints affecting service continuity, notably irregular commodity supplies and dependence on external funding. Taken together, these findings indicate that "maintenance" was achieved in terms of organizational presence and program footprint, but not uniformly in terms of routine operational performance**.** The divergence between standardized databases and implementers' perceptions underscores the need for systematic triangulation of information sources, improved data quality systems, and stronger supply-chain monitoring to accurately assess the stability and sustainability of community-based interventions (Table 4).

Table 4: Summary of quantitative and qualitative findings related to Maintenance and implementation conditions, Guinea, 2021–2024

**Element**	**Indicator / Quantitative evidence**	**Qualitative corroboration**	**Illustrative verbatims**	**Suggested actions**
**Organizational stability**	26 active CBOs; median tenure = 3 years (2022–2024)	Implementers highlighted sustained CBO engagement and local legitimacy	*"The advantage we had was selecting NGOs that were already active in the field."* (Coordinator 1)	Formalize CBO roles in district health plans; establish MOUs and routine supervision schedules

**Data coherence**	~19.2% of CBOs annually showed referral-to-test discrepancies; 7.7–11.5% presented atypical intra-annual tested/referred fluctuations	Staff reported mismatches between program databases and field realities	*"At the beginning we did not have a well-established strategy; we had to adapt the health centre tools."* (Coordinator 1)	Strengthen routine data validation, introduce triangulation checks, and train CBO data focal points
**Commodity security**	No official programmatic stockout records; implementers nevertheless reported intermittent supply interruptions	Several implementers cited irregular HIV test kit supply as affecting continuity	*"The biggest problem we had was with inputs, since some health centre managers were reluctant to supply CBOs."*	Institutionalize supply chain reporting at CBO level; establish buffer stocks and rapid resupply procedures
**Financial sustainability**	Ongoing dependence on external funding reported	Concerns raised about continuity if external support declines	*"After CBO training in 2020, activities only began in 2021 because we had not received funding."*	Develop phased domestic financing plans; diversify funding and formalize cost-sharing mechanisms
**Human resources and supervision**	Reports of variable training quality, high turnover, and	Implementers highlighted gaps in refresher training and logistical support	*"There are many changes among community counselors;*	Implement regular refresher training, standardized supervision tools,

	limited supervision		*sometimes allowances are delayed, and newcomers need training.”*	and retention incentives
**Confidentiality and stigma**	Identified as a constraint for linkage and test uptake	Fear of disclosure within households frequently reported	*“Only my children know I am sick, because if other family members knew, I would be rejected.”* (Index case 6)	Strengthen confidentiality protocols, counselor training, and community stigma-reduction activities
**Community ownership**	Continued involvement of local leaders and CBOs documented	Reports described local leaders organizing awareness sessions and enhancing legitimacy	*“When we enter a community, before starting sensitization, we approach the local leaders; they give us the green light and their support.”* (CBO 11)	Sustain and formalize community leadership roles; involve local leaders in performance reviews

Mechanisms of impact of the intervention

The five RE-AIM dimensions describe multidimensional programme dynamics. Expansion of coverage (Reach) was accompanied by household exposure to HIV testing activities, whereas Effectiveness indicators displayed heterogeneous patterns. Linkage to care was substantially higher among women and children than among men,

revealing a marked sex-related gap in engagement with the care pathway following an HIV-positive result. Across the RE-AIM dimensions, periods of program expansion, reflected by increased organizational adoption and geographic coverage, were temporally associated with higher activity volumes, while also coinciding with greater variability in effectiveness and maintenance indicators. Implementation patterns evolved through phases of intensity, adjustment, and expansion, with performance varying in relation to session intensity and inter-CBO stability. Qualitative analyses suggested several operational factors perceived to support program functioning, including community counseling, PMTCT mentorship, and longitudinal follow-up, that enhanced trust and continuity of care. They also identified recurrent structural and operational constraints, notably, commodity stockouts, reliance on external funding, and difficulties with intra-household disclosure. These factors were perceived by participants as influencing the conversion of sensitization into testing uptake and linkage to care. Integrated quantitative – qualitative triangulation helped identify patterns not fully captured by routine indicators. Taken together, these observations suggest that sustaining program performance may depend on balancing coverage expansion with operational stability, including strengthening supply chain reliability and institutional support mechanisms (S7 Fig).

Discussion

This study, guided by the RE-AIM framework, evaluated the implementation and effectiveness of an index-assisted community HIV testing strategy in Guinea from 2021 to 2024. The intervention reached more than 50,000 individuals, but performance varied across the cascade, including marked age- and sex-related differences in linkage to care. Three principal findings frame the discussion: (1) variable effectiveness across implementation phases and population subgroups; (2) a three-phase implementation

trajectory, initial intensity, organizational adjustment, and territorial expansion; and (3) fragile institutional sustainability.

The index approach documented substantial community-based testing activity and linkage to care among individuals diagnosed during routine implementation. The markedly lower adjusted probability of linkage among men represents an important implementation gap. Comparable studies in African settings have documented lower engagement of men in HIV testing and care pathways than women, reflecting gender-related differences in healthcare use, stigma, disclosure concerns, and service accessibility [44,45] . In the present study, qualitative findings concerning stigma and difficulties with intra-household disclosure provide plausible contextual explanations. However, the analysis did not directly collect male-specific reasons for non-referral, and the observed difference should not be interpreted as direct evidence that men refused to accept their HIV status. Rather, it indicates lower male engagement with the post-diagnosis care pathway, which may reflect a combination of individual, household, community, and service-level barriers.

Lower male engagement may reduce opportunities for partner support and complicate voluntary and safe disclosure within households. Nevertheless, this evaluation did not assess women's retention according to their partners' referral status or measure intimate-partner or gender-based violence outcomes; no causal relationship can therefore be inferred. Male-responsive strategies, including trained male peer navigators or trusted community champions, confidential counselling, flexible service-delivery modalities, and supported referral, should be prospectively evaluated. Such approaches should also incorporate voluntary disclosure options, confidential assessment of disclosure-related safety, and appropriate referral pathways for individuals at risk of violence [46–48]. Nevertheless, pronounced heterogeneity in

referral rates highlights persistent structural constraints. As reported in Burkina Faso, irregular availability of commodities and stigma-related barriers limited cascade performance [49,50]. Qualitative interviews indicated that difficulties in disclosing serostatus to partners are an important deterrent to care-seeking, thereby reducing the intervention's net impact. In our study, the sharp decline in referral observed in 2023 occurred during the rapid expansion phase and may reflect a combination of operational instability and reporting constraints. Implementers reported turnover among community counsellors, requiring training and a transition period to rebuild trust with index cases and households, as well as recurrent interruptions or irregular supply of HIV testing commodities affecting community testing and referral pathways. In addition, 2023 coincided with preparation of the Global Fund funding request for 2024–2026, which may have temporarily affected routine supervision, reporting and data validation processes. Collectively, these observations suggest that, despite favourable implementation outcomes in several cascade steps, index testing remains vulnerable to local resource dynamics, sociocultural norms, and coordination gaps, as documented in other West African studies [51–59]. However, the relative contribution of these factors cannot be quantified with precision using routine programme data alone**.**

The three-phase implementation pattern reflects organizational learning during scale-up [60–63] . The expansion phase may have introduced a dilution effect: as the programme broadened beyond initial high-priority networks, diagnostic yield and cascade conversion may have become more variable. This pattern, reported in West and Southern Africa, indicates that territorial expansion must be accompanied by adaptive targeting and revised operational criteria to preserve effectiveness while maintaining access [64–66].

Sustainability of the community testing model in Guinea depends on two interdependent conditions: the long-term viability of external financing amid declining international aid, and formal integration of the approach within national health systems. This dual dependence creates structural fragility that has undermined community interventions elsewhere in sub-Saharan Africa when external funding ceased [67,68]. In Guinea, the lack of institutionalized supply mechanisms for CBOs exacerbated vulnerability: intermittent commodity shortages were reported by several implementers, despite no documented “stockout” events in routine program databases. This discrepancy exposes limitations of standard monitoring indicators and underscores the value of systematic triangulation between quantitative records and qualitative inquiry to detect operational interruptions. The RE-AIM framework highlighted programme expansion, reflected by increased organizational adoption and geographic coverage, coincided with higher reported activity volumes, while also revealing variability in effectiveness and maintenance indicators. These multidimensional tensions would likely have remained undetected in evaluations focused solely on biomedical outcomes.

This study has several methodological limitations. Its retrospective design and reliance on routine programmatic data may have introduced reporting and completeness biases. The study did not directly examine the reasons why HIV-positive men were not linked to care, assess women’s treatment retention according to their partners’ referral status, or measure adverse social outcomes such as unwanted disclosure, intimate-partner violence, or other forms of gender-based violence. The findings therefore identify a substantial male-engagement gap but do not establish its underlying mechanisms or its consequences for female partners’ treatment outcomes. The absence of a contemporaneous comparison group limits causal inference, and residual inter-site variability suggests that unmeasured contextual factors, such as local

coordination capacity, supply consistency, and population mobility, may have influenced outcomes. Nevertheless, the study presents notable strengths, including broad geographic coverage across 17 districts, methodological triangulation combining quantitative and qualitative data, and the structured application of the RE-AIM framework to evaluate a national-scale community-based HIV testing strategy.

Policy and programmatic implications follow directly. Findings from the Maintenance dimension indicate that long-term sustainability remains vulnerable due to reliance on external funding, incomplete integration into national budgeting mechanisms, and gaps in commodity supply reporting at the CBO level. Strengthening institutional integration within district health planning processes and national financing frameworks may enhance stability of community-based index testing. Structural integration of community-based organizations into the formal health system can enhance service reach among hard-to-reach and at-risk populations, while reducing attrition along the cascade. Programmes should consider a male-responsive post-diagnosis support package combining trained male peer navigators or trusted community champions, confidential counselling, flexible service hours and referral options, and active accompaniment to HIV care. Because partner engagement and disclosure may raise safety concerns, these approaches should remain voluntary and include confidential assessment of disclosure-related risks and functional referral pathways for psychosocial support and protection. Their acceptability, effectiveness, and potential unintended consequences should be assessed prospectively. Financial sustainability requires the progressive incorporation of operational costs into national health budgets and proactive mobilization of domestic co-financing mechanisms to reduce dependence on external donors.

Future research should assess the cost-effectiveness of this model to guide evidence-based resource allocation. Future studies should prospectively evaluate male-responsive and peer-supported strategies using sex-disaggregated outcomes for post-diagnosis referral, linkage to care, ART initiation, and retention. They should document male-specific reasons for engagement or non-engagement, examine whether male partner linkage is associated with women's retention in care, and systematically monitor unintended social harms, including unwanted disclosure and intimate-partner or gender-based violence. Quasi-experimental studies including comparison groups could strengthen causal inference, while targeted qualitative analyses may identify the most effective operational levers to sustain performance during scale-up. Longitudinal cohort studies are also warranted to evaluate long-term outcomes in treatment adherence and retention.

Conclusion

Overall, the findings illustrate the operational performance of the integrated "*community – clinical index testing model*" in identifying HIV-positive individuals and facilitating linkage to care during the implementation period. However, the markedly lower probability of linkage among men identifies a critical gap in the community-clinical pathway. Programmes should consider and rigorously evaluate male-responsive, peer-supported, and safety-centred approaches to improve men's engagement with HIV care following diagnosis. Consolidation of supply chains, institutional support for community-based organizations, and sustainable financing are critical to ensuring durability. These findings contribute to the evidence based on large-scale implementation of community-based index testing under routine programme conditions, highlighting factors relevant to programme performance and sustainability.

Data availability statement

All data underlying the findings of this study are provided as Supporting Information. The quantitative analyses were conducted using a de-identified aggregated dataset of quarterly programme monitoring data, included as S8 Data. No individual-level identifiers are included in the shared dataset. These data are sufficient to reproduce the analyses reported in the manuscript. Data sharing is consistent with the ethical approval granted by the National Ethics Committee for Health Research in Guinea and complies with applicable confidentiality requirements.

Acknowledgments

We thank all study participants for sharing their perspectives on community-based HIV testing. We acknowledge the National HIV and Hepatitis Control Program (PNLSH) and Fraternité Médicale Guinée (FMG) for facilitating access to study sites, community-based organizations, and programme records. We also thank the Africa Centre of Excellence for Prevention and Control of Communicable Diseases and Cheikh Anta Diop University of Dakar for their academic and institutional support. We acknowledge the use of AI-assisted tools, including ChatGPT, for language editing. All authors reviewed and approved the final manuscript and take full responsibility for its content.

References

1. UNAIDS. Global AIDS Update : The Path That Ends AIDS. Geneva; 2023. Available: https://www.unaids.org/en/resources/documents/2023/global-aids-update
2. UNAIDS. Global AIDS report : The Urgency of Now: AIDS at a Crossroads. In: AIDS report 2024 [Internet]. 2024. Available: https://www.unaids.org/en/resources/documents/2024/global-aids-update-2024
3. Akullian A, Akulu R, Aliyu G, Anam F, Guichard AC, Ayles H, et al. The HIV response beyond 2030: preparing for decades of sustained HIV epidemic control in eastern and southern Africa. The Lancet. 2024;404: 638–641. doi:10.1016/S0140-6736(24)00980-2
4. Hlongwa M, Moyo E, Dzinamarira T. Approaches for improving linkage to HIV care among HIV self-testing individuals in sub-Saharan Africa. BMJ Glob Health. 2023;8: e012664. doi:10.1136/BMJGH-2023-012664
5. World Health Organization (WHO). Consolidated Guidelines on HIV Prevention, Testing, Treatment, Service Delivery and Monitoring: Recommendations for a Public Health Approach, Geneva. Jul 2025. Available: https://pubmed.ncbi.nlm.nih.gov/34370423/
6. World Health Organization (WHO). HIV and AIDS report. 15 Jul 2025. Available: https://www.who.int/news-room/fact-sheets/detail/hiv-aids
7. Boye S, Kouadio A, Kouvahe AF, Vautier A, Ky-Zerbo O, Rouveau N, et al. Organisation of testing services, structural barriers and facilitators of routine HIV self-testing during sexually transmitted infection consultations: a qualitative study of patients and providers in Abidjan, Côte d’Ivoire. BMC Infect Dis. 2022;22. doi:10.1186/S12879-023-08625-X,
8. Seidu A-A. Using Anderson’s Model of Health Service Utilization to Assess the Use of HIV Testing Services by Sexually Active Men in Ghana. Front Public Health. 2020;8: 512. doi:10.3389/fpubh.2020.00512
9. Lakhe NA, Diallo Mbaye K, Sylla K, Ndour CT. HIV screening in men and women in Senegal: coverage and associated factors; analysis of the 2017 demographic and health survey. BMC Infect Dis. 2019;20: 1. doi:10.1186/S12879-019-4717-5
10. Rouveau N, Ky-Zerbo O, Boye S, Fotso AS, d’Elbée M, Maheu-Giroux M, et al. Describing, analysing and understanding the effects of the introduction of HIV self-testing in West Africa through the ATLAS programme in Côte d’Ivoire, Mali and Senegal. BMC Public Health. 2021;21. doi:10.1186/s12889-021-10212-1
11. Morin L, Béhanzin L, Guédou FA, Kêkê RK, Bushman L, Anderson PL, et al. HIV Prevention and Treatment Cascades among Female Sex Workers in Benin, West Africa. Sex Transm Dis. 2021;48: 654–662. doi:10.1097/OLQ.0000000000001399
12. Hatzold K, Dunkley Y. Effects of self-testing on HIV outcomes in west Africa. Lancet HIV. 2024;11: e498–e500. doi:10.1016/S2352-3018(24)00179-6
13. Dalal S, Johnson C, Fonner V, Kennedy CE, Siegfried N, Figueroa C, et al. Improving HIV test uptake and case finding with assisted partner notification services. AIDS. 2017;31: 1867–1876. doi:10.1097/QAD.0000000000001555
14. Assefa E, Figa Z. Acceptance of the Index Case HIV Testing and Associated Factors among Anti-Retroviral Therapy Patients in Public Health Facilities in Gedeo Zone, Southern Ethiopia. Cross-Sectional Study Design with Document Review. Inquiry (United States). 2024;61. doi:10.1177/00469580241271185
15. Gizaw YT, Boke MM, Geremew AB. Determinants of hiv testing uptake among partners of pregnant women in addis ababa, ethiopia: A community-based study. Pan African Medical Journal. 2021;39. doi:10.11604/pamj.2021.39.7.27839

16. Lindsay BR, Mwango L, Toeque MG, Malupande SL, Nkhuwa E, Moonga CN, et al. Peer community health workers improve HIV testing and ART linkage among key populations in Zambia: retrospective observational results from the Z-CHECK project, 2019–2020. J Int AIDS Soc. 2022;25. doi:10.1002/jia2.26030
17. Tarekegn D, Habtegiorgis SD, Telayneh AT, Mitiku KW, Adugna A, Alemayehu D, et al. The Magnitude and Associated Factors of Early Index Case Testing Among Adult HIV Index Cases at Debre Markos Town High Load Health Facilities 2023. AIDS Res Treat. 2025: 8237131. doi:10.1155/arat/8237131
18. Onovo A, Kalaiwo A, Agweye A, Emmanuel G, Keiser O. Diagnosis and case finding according to key partner risk populations of people living with HIV in Nigeria: A retrospective analysis of community-led index partner testing services. EClinicalMedicine. 2022;43. doi:10.1016/j.eclinm.2021.101265
19. Munthali T, Masano A, Chen JS, Kumwenda W, Pedersen C, Chinthenga K, et al. Assisted partner notification as a strategy to enhance Pre-Exposure Prophylaxis (PrEP) screening and uptake – results from a prospective cohort study in Lilongwe, Malawi. BMC Health Serv Res. 2025;25. doi:10.1186/s12913-025-13038-4
20. Khandu L, Hallett J, Crawford G, Leavy JE, Vujcich D. HIV self-testing and partner notification strategies for key populations in low- to upper-middle-income countries: A mixed-methods systematic review. PLoS One. 2025;20. doi:10.1371/journal.pone.0338639
21. Brown LB, Miller WC, Kamanga G, Nyirenda N, Mmodzi P, Pettifor A, et al. HIV partner notification is effective and feasible in sub-Saharan Africa: Opportunities for HIV treatment and prevention. J Acquir Immune Defic Syndr (1988). 2011;56: 437–442. doi:10.1097/QAI.0b013e318202bf7d
22. Mugisha N, Tirera F, Coulibaly-Kouyate N, Aguie W, He Y, Kemper K, et al. Implementation process and challenges of index testing in Côte d'Ivoire from healthcare workers' perspectives. PLoS One. 2023;18: e0280623. doi:10.1371/journal.pone.0280623
23. Mshweshwe-Pakela N, Mabuto T, Lebyane R, Hamisi M, Gininza S, Chihota V, et al. HIV index testing implementation at public health clinics in South Africa. S Afr Med J. 2025;115. doi:10.7196/SAMJ.2025.v115i8.2652
24. Meek CJ, Munkhondya TEM, Mphande M, Tembo TA, Chitani M, Jean-Baptiste M, et al. Examining the feasibility of assisted index case testing for HIV case-finding: a qualitative analysis of barriers and facilitators to implementation in Malawi. BMC Health Services Research 2024 24:1. 2024;24: 606-. doi:10.1186/s12913-024-10988-z
25. Henley C, Forgwei G, Welty T, Golden M, Adimora A, Shields R, et al. Scale-up and case-finding effectiveness of an HIV partner services program in Cameroon: An innovative HIV prevention intervention for developing countries. Sex Transm Dis. 2013;40: 909–914. doi:10.1097/OLQ.0000000000000032
26. Perera S, Swartz A, Githaiga JN. "Getting involved": understanding informal practices, challenges and alternatives in assisted partner notification services for HIV in Cape Town, South Africa. AIDS Care. 2026 [cited 26 Feb 2026]. doi:10.1080/09540121.2026.2621031
27. Camara S, Millimouno TM, Kourouma M, Sow A, Sidibé S, Touré AB, et al. Facilitators and barriers to community-based HIV testing in Guinea: a CFIR-based implementation analysis. Front Public Health. 2025;13: 1593697. doi:10.3389/fpubh.2025.1593697
28. Bärnighausen K, Berner-Rodoreda A, McGowan M, Reñosa MD, Mtaita C, Neuhann F. 'You Can Get That Person on ART but You Can't Give Them Back Their Social System': A Qualitative Analysis of Voluntary Assisted Partner Notification for HIV for

Marginalised and Vulnerable Populations. J Int Assoc Provid AIDS Care. 2024;23. doi:10.1177/23259582241272059
29. Lewis ZH, Ottenbacher KJ, Fisher SR, Jennings K, Brown AF, Swartz MC, et al. The feasibility and RE-AIM evaluation of the TAME health pilot study. Int J Behav Nutr Phys Act. 2017;14. doi:10.1186/S12966-017-0560-5
30. Proctor E, Silmere H, Raghavan R, Hovmand P, Aarons G, Bunger A, et al. Outcomes for implementation research: conceptual distinctions, measurement challenges, and research agenda. Adm Policy Ment Health. 2011;38: 65–76. doi:10.1007/s10488-010-0319-7
31. UNAIDS. UNAIDS data 2022 | UNAIDS. In: report AIDS 2022 [Internet]. 2023 [cited 15 Sep 2025]. Available: https://www.unaids.org/en/resources/documents/2023/2022_unaids_data
32. The Global Funds. Office of the Inspector General . Guinea: Grant Audit Report. Geneva. 2024 Dec. Available: https://www.theglobalfund.org/media/15325/oig_gf-oig-24-019_report_fr.pdf?utm_source=chatgpt.com
33. Mugisha N, Tirera F, Coulibaly-Kouyate N, Aguie W, He Y, Kemper K, et al. Implementation process and challenges of index testing in Côte d’Ivoire from healthcare workers’ perspectives. PLoS One. 2023;18: e0280623. doi:10.1371/journal.pone.0280623
34. Meek CJ, Munkhondya TEM, Mphande M, Tembo TA, Chitani M, Jean-Baptiste M, et al. Examining the feasibility of assisted index case testing for HIV case-finding: a qualitative analysis of barriers and facilitators to implementation in Malawi. Res Sq. 2023; rs.3.rs-3314925. doi:10.21203/rs.3.rs-3314925/v1
35. Craig P, Dieppe P, Macintyre S, Mitchie S, Nazareth I, Petticrew M. Developing and evaluating complex interventions: the new Medical Research Council guidance. The BMJ. 2008;337: a1655. doi:10.1136/bmj.a1655
36. Consolidated guidelines on HIV testing services (2015). [cited 7 Nov 2025]. Available: https://www.who.int/publications/i/item/9789241508926?
37. D’Lima D, Soukup T, Hull L. Evaluating the Application of the RE-AIM Planning and Evaluation Framework: An Updated Systematic Review and Exploration of Pragmatic Application. Front Public Health. 2022;9. doi:10.3389/FPUBH.2021.755738/PDF
38. Burnham KP, Anderson DR. Multimodel inference: Understanding AIC and BIC in model selection. Sociol Methods Res. 2004;33: 261–304. doi:10.1177/0049124104268644
39. Brewer MJ, Butler A, Cooksley SL. The relative performance of AIC, AICC and BIC in the presence of unobserved heterogeneity. Methods Ecol Evol. 2016;7: 679–692. doi:10.1111/2041-210X.12541
40. Burnham KP, Anderson DR, Huyvaert KP. AIC model selection and multimodel inference in behavioral ecology: Some background, observations, and comparisons. Behav Ecol Sociobiol. 2011;65: 23–35. doi:10.1007/S00265-010-1029-6
41. Zamawe FC. The Implication of Using NVivo Software in Qualitative Data Analysis: Evidence-Based Reflections. Malawi Medical Journal. 2015;27: 13. doi:10.4314/MMJ.V27I1.4
42. Gale NK, Heath G, Cameron E, Rashid S, Redwood S. Using the framework method for the analysis of qualitative data in multi-disciplinary health research. BMC Med Res Methodol. 2013;13. doi:10.1186/1471-2288-13-117
43. Hoff TJ, Amick HR, Lund JL, Lee SYD. Review: Use of Qualitative Methods in Published Health Services and Management Research: A 10-Year Review. Medical Care Research and Review. 2011;68: 3–33. doi:10.1177/1077558710372810

44. Hlongwa M, Basera W, Hlongwana K, Lombard C, Laubscher R, Duma S, et al. Linkage to HIV care and early retention in HIV care among men in the 'universal test-and-treat' era in a high HIV-burdened district, KwaZulu-Natal, South Africa. BMC Health Services Research 2024 24:1. 2024;24: 384-. doi:10.1186/S12913-024-10736-3
45. Hlongwa M, Hlongwana K, Makhunga S, Choko AT, Dzinamarira T, Conserve D, et al. Linkage to HIV Care Following HIV Self-testing Among Men: Systematic Review of Quantitative and Qualitative Studies from Six Countries in Sub-Saharan Africa. AIDS and Behavior 2022 27:2. 2022;27: 651–666. doi:10.1007/S10461-022-03800-8
46. Lippman SA, De Kadt J, Ratlhagana MJ, Agnew E, Gilmore H, Sumitani J, et al. Impact of short message service and peer navigation on linkage to care and antiretroviral therapy initiation in South Africa. AIDS. 2023;37: 647–657. doi:10.1097/QAD.0000000000003453
47. Tavakoli F, Karamouzian M, Haghdoost AA, Mirzazadeh A, Dehghan M, Bazrafshani MS, et al. Barriers and facilitators of HIV partner status notification in low- and lower-middle-income countries: A mixed-methods systematic review. BMC Infectious Diseases 2024 24:1. 2024;24: 1404-. doi:10.1186/S12879-024-10241-2
48. Sanena M, Hagstrom C, Phiri K, Kalande P, Songo J, Maruwo L, et al. Intimate partner violence among HIV index case testing clients in Malawi, a mixed-methods study. PLOS Global Public Health. 2025;5: e0003818. doi:10.1371/JOURNAL.PGPH.0003818
49. Traore IT, Meda N, Hema NM, Ouedraogo D, Some F, Some R, et al. HIV prevention and care services for female sex workers: Efficacy of a targeted community-based intervention in Burkina Faso. J Int AIDS Soc. 2015;18. doi:10.7448/IAS.18.1.20088
50. Kirakoya-Samadoulougou F, Jean K, Maheu-Giroux M. Uptake of HIV testing in Burkina Faso: an assessment of individual and community-level determinants. BMC Public Health. 2017;17. doi:10.1186/s12889-017-4417-2
51. Boisvert Moreau M, Kintin FD, Atchekpe S, Batona G, Béhanzin L, Guédou FA, et al. HIV self-testing implementation, distribution and use among female sex workers in Cotonou, Benin: a qualitative evaluation of acceptability and feasibility. BMC Public Health. 2022;22. doi:10.1186/S12889-022-12917-3
52. Oyebade AO, Bello MA, Familusi IO. Effect of the antenatal hiv testing scale up community intervention in south west, nigeria: A cross-sectional study. Pan African Medical Journal. 2021;40. doi:10.11604/pamj.2021.40.90.30230
53. Mohammed A, Cheabu BSN, Amoah-Larbi J, Osei FA, Benyah G, Asampong R, et al. Perception and experience of HIV-induced stigma among people with HIV seeking healthcare in Ghana. BMC Health Serv Res. 2024;24. doi:10.1186/S12913-024-11930-Z
54. Coulaud PJ, Préau M, Maradan G, Mora M, Traoré F, Oga M, et al. Taking empowerment into account: the response of community-based organisations to the HIV care needs of men who have sex with men in West Africa (CohMSM ANRS 12324 - Expertise France). AIDS Care. 2019;31: 1403–1411. doi:10.1080/09540121.2019.1587358
55. Arije O, Titus R, Olaniran A, Dadi A, Garba D, Okeke E, et al. Effectiveness of community mobilisation models in improving HIV testing services uptake among women and children in Nigeria: A quasi-experimental study. Glob Public Health. 2023;18. doi:10.1080/17441692.2023.2284880
56. Arije O, Titus R, Omisile I, Dadi A, Garba D, Godpower O, et al. Process evaluation of the 'Lafiyan Yara' project on enhancing access to HIV testing services using existing community structures in Nigeria. BMC Public Health. 2024;24. doi:10.1186/s12889-024-18045-4

57. Camara S, Millimouno TM, Kourouma M, Sow A, Sidibé S, Touré AB, et al. Facilitators and barriers to community-based HIV testing in Guinea: a CFIR-based implementation analysis. Front Public Health. 2025;13: 1593697. doi:10.3389/fpubh.2025.1593697
58. Perray M, Traore D, Riegel L, Rojas Castro D, Spire B, Mora M, et al. Benefits and challenges of a community-based programme for women living with HIV in Mali. AIDS Care. 2023;35: 1100–1106. doi:10.1080/09540121.2023.2208319
59. Abubakari GMR, Turner DA, Ni Z, Conserve DF, Dada D, Otchere A, et al. Community-Based Interventions as Opportunities to Increase HIV Self-Testing and Linkage to Care Among Men Who Have Sex With Men – Lessons From Ghana, West Africa. Front Public Health. 2021;9. doi:10.3389/fpubh.2021.660256
60. Ngcobo S, Scheepers S, Mbatha N, Grobler E, Rossouw T. Roles, Barriers, and Recommendations for Community Health Workers Providing Community-Based HIV Care in Sub-Saharan Africa: A Review. AIDS Patient Care STDS. 2022;36: 130–144. doi:10.1089/apc.2022.0020
61. Hlongwa M, Mashamba-Thompson T, Makhunga S, Hlongwana K. Barriers to HIV testing uptake among men in sub-Saharan Africa: a scoping review. Afr J AIDS Res. 2020;19: 13–23. doi:10.2989/16085906.2020.1725071
62. Chikwari CD, Dringus S, Ferrand RA. Barriers to, and emerging strategies for, HIV testing among adolescents in sub-Saharan Africa. Curr Opin HIV AIDS. 2018;13: 257–264. doi:10.1097/COH.0000000000000452
63. Donessouné FMG, Sossa OG, Kouanda S. Using CFIR framework for understanding barriers and facilitators to implementation of community tuberculosis program in Burkina Faso. Frontiers in Health Services. 2023;3: 1294176. doi:10.3389/FRHS.2023.1294176/BIBTEX
64. Conserve DF, Msofe J, Issango J, Tureski K, McCarthy P, Rwezahura P, et al. Development, Implementation, and Scale Up of the National Furaha Yangu Campaign to Promote HIV Test and Treat Services Uptake Among Men in Tanzania. Am J Mens Health. 2022;16. doi:10.1177/15579883221087838
65. Grabowski MK, Mpagazi J, Kiboneka S, Ssekubugu R, Kereba JB, Nakayijja A, et al. The HIV and sexually transmitted infection syndemic following mass scale-up of combination HIV interventions in two communities in southern Uganda: a population-based cross-sectional study. Lancet Glob Health. 2022;10: e1825–e1834. doi:10.1016/S2214-109X(22)00424-7
66. Oyebade AO, Bello MA, Familusi IO. Effect of the antenatal hiv testing scale up community intervention in south west, nigeria: A cross-sectional study. Pan African Medical Journal. 2021;40. doi:10.11604/pamj.2021.40.90.30230
67. Okere NE, Lennox L, Urlings L, Ford N, Naniche D, Rinke De Wit TF, et al. Exploring Sustainability in the Era of Differentiated HIV Service Delivery in Sub-Saharan Africa: A Systematic Review. J Acquir Immune Defic Syndr. 2021;87: 1055–1071. doi:10.1097/QAI.0000000000002688
68. Nwaozuru U, Murphy P, Richard A, Obiezu-Umeh C, Shato T, Obionu I, et al. The sustainability of health interventions implemented in Africa: an updated systematic review on evidence and future research perspectives. Implement Sci Commun. 2025;6. doi:10.1186/S43058-025-00716-X

Supporting information

S1 Fig (TIF): Geographic distribution of intervention sites for community-based HIV testing in Guinea (2021–2024). Pilot and scale-up districts implementing community index testing through community-based organizations (CBOs) linked to PMTCT sites**.** Map produced in QGIS v3.44.4 using administrative boundaries from GADM (https://gadm.org/; https://gadm.org/license.html).

S2 Fig: Program theory linking PMTCT sites and community-based organizations (CBOs) within the HIV index case testing cascade in Guinea (2021–2024). The figure illustrates the hypothesized pathways connecting community sensitization, testing, and link (TIF)

S3 Table. Operationalization of RE-AIM. (DOCX)
Definition of the RE-AIM dimensions and corresponding indicators, including calculation methods, data sources, and analytical approaches used to evaluate the community-based HIV index case testing program in Guinea (2021–2024).

S4 Text. Detailed statistical analysis. (DOCX)
Description of study variables, mixed-effects modelling approach, diagnostics, sensitivity analyses, and statistical software/packages used to assess HIV test acceptance, referral, and ART initiation outcomes (Guinea, 2021–2024).

S5 Table. NVivo codebook aligned with the RE-AIM framework. (DOCX)
Qualitative coding framework used for thematic analysis, including operational definitions of codes by RE-AIM dimension and representative illustrative quotes from key informants and beneficiaries.

S6 Table. Implementation phases of the community-based HIV testing program. (DOCX)
Summary of implementation phases (2021–2023), including intensity, operational objectives, key mechanisms, and expected quantitative signals used to interpret program performance over time.

S7 Fig. Mechanism of impact of community-based organizations in the index case HIV testing cascade (Guinea, 2021–2024). (TIF)

Conceptual diagram illustrating how CBO activities contribute to key cascade steps, from sensitization and contact elicitation to testing, referral, and linkage to care.

S8 Data. De-identified aggregated dataset and codebook (XLSX).

Anonymized aggregated quarterly program dataset used for quantitative analyses of community-based index case HIV testing in Guinea (2021–2024), including codebook and README.

Figure

Click here to access/download;Figure;Fig1.tif

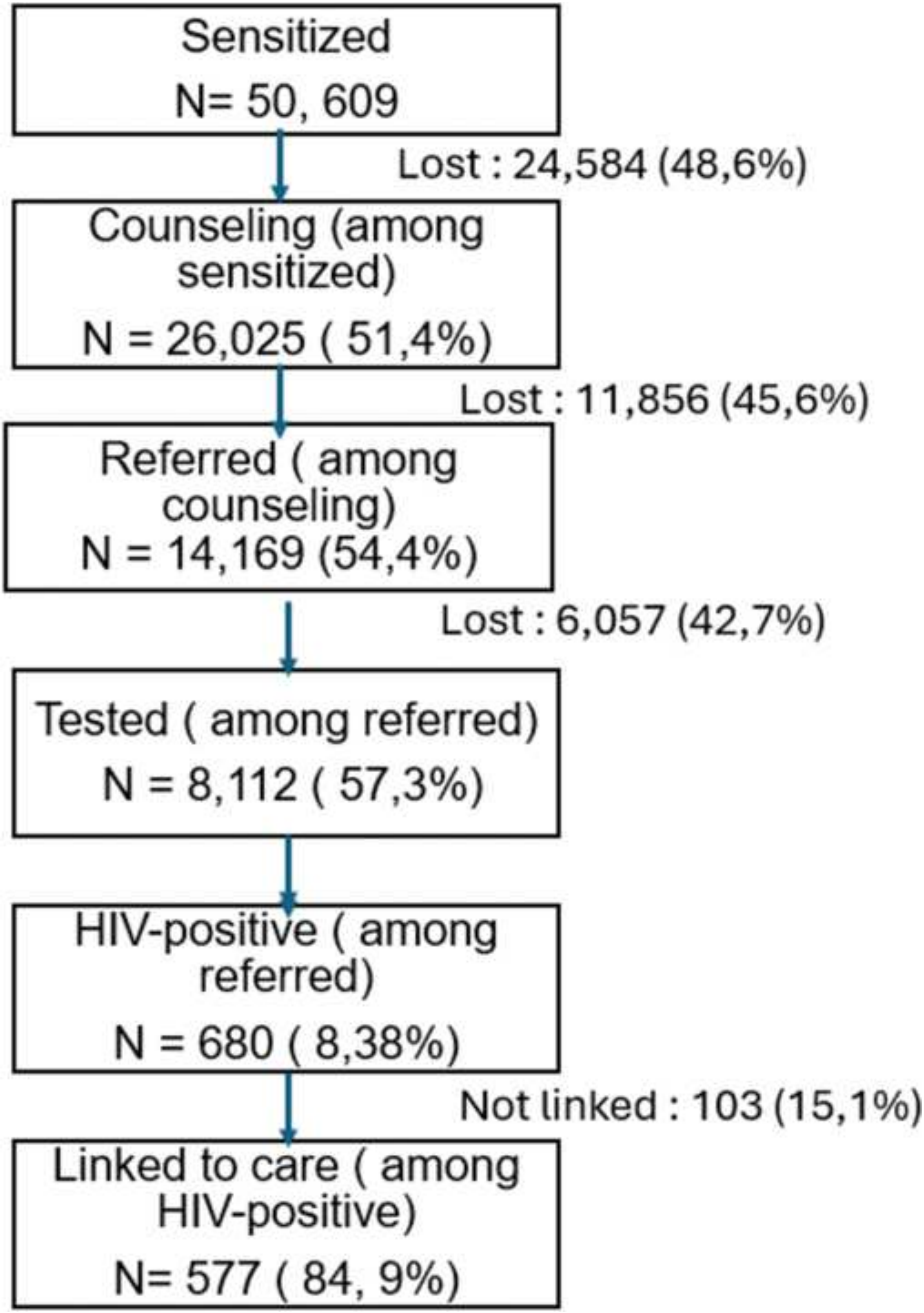

 Click here to access/download;Figure;Fig 2.tif

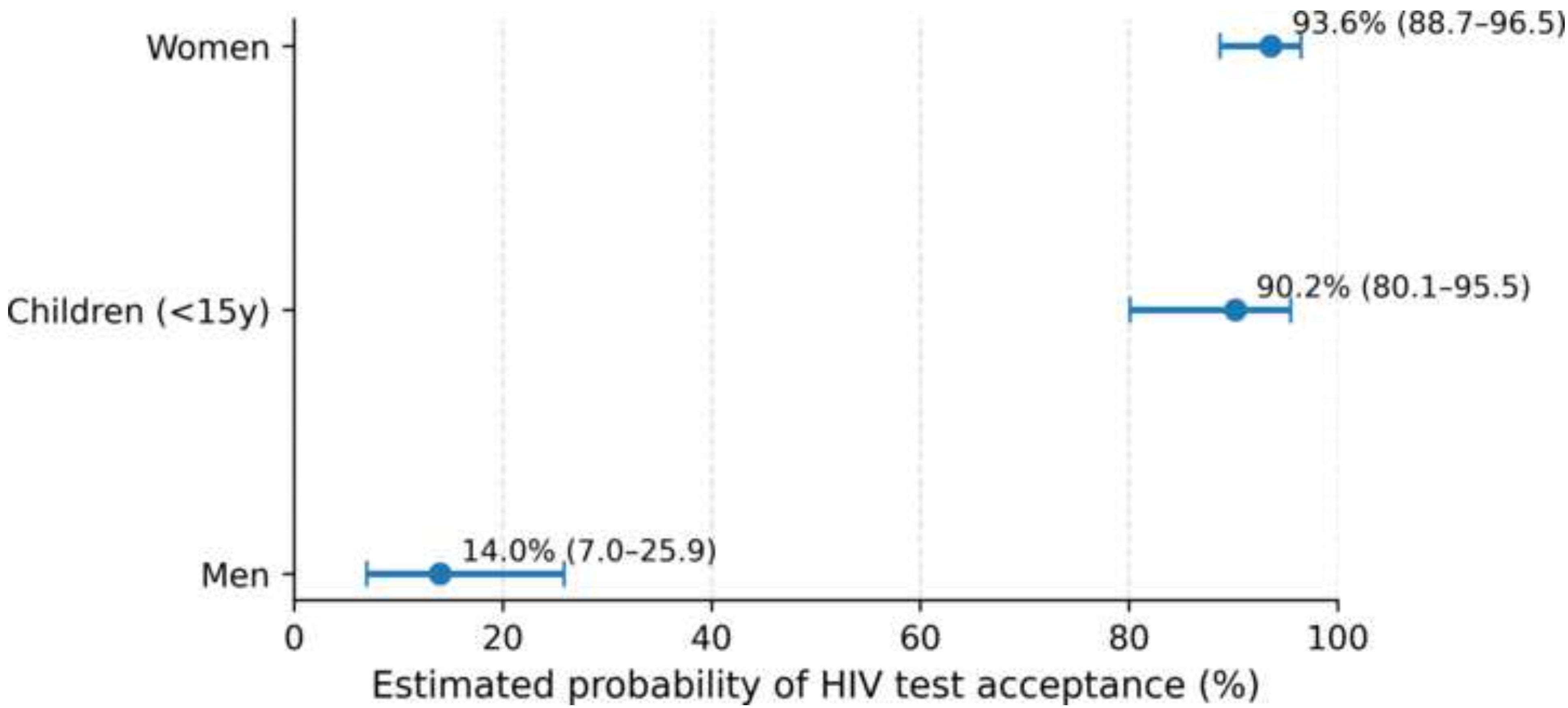

Figure

Click here to access/download;Figure;Fig 3.tif

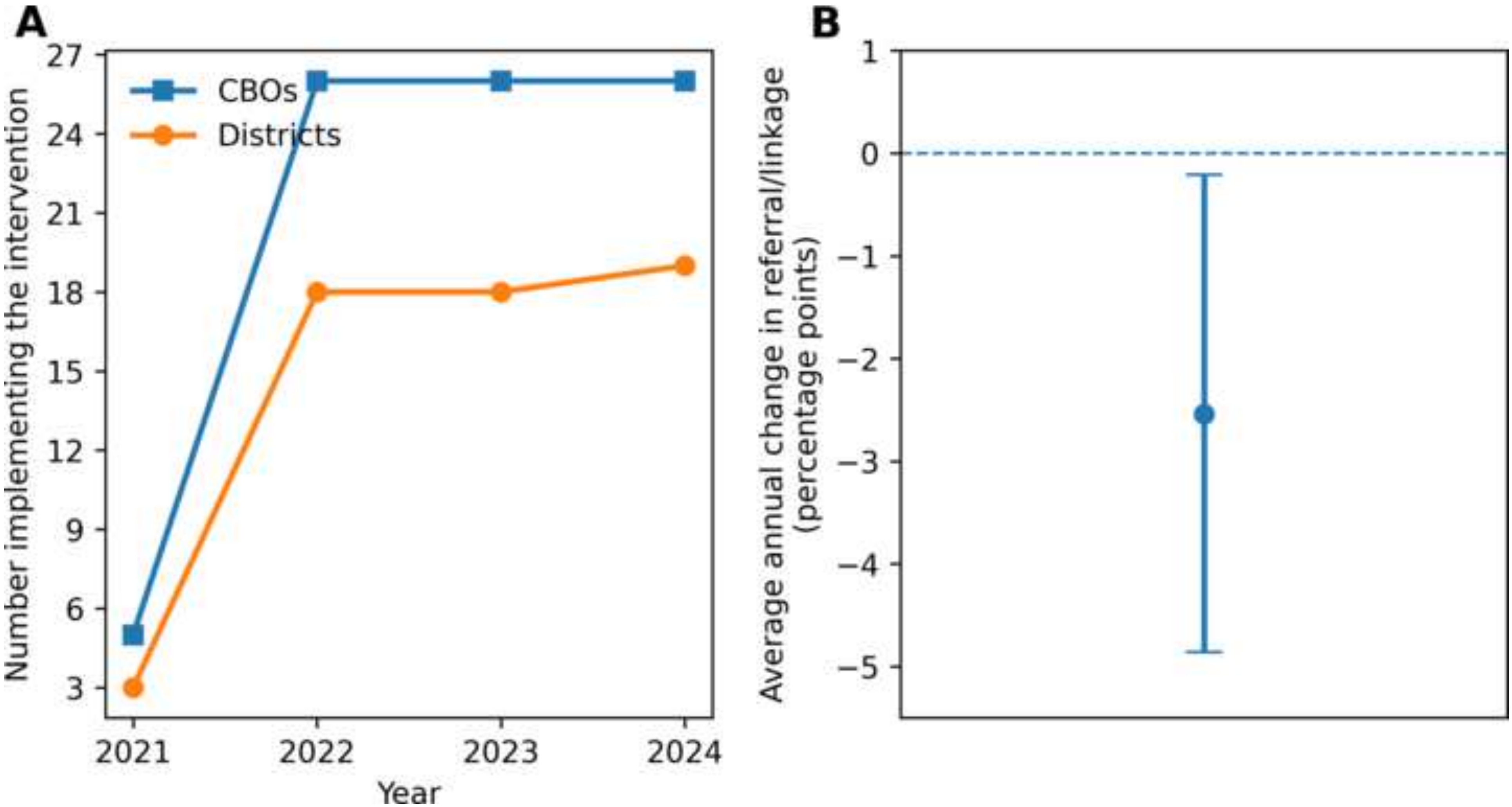

Figure

Click here to access/download;Figure;Fig2_rev.tif

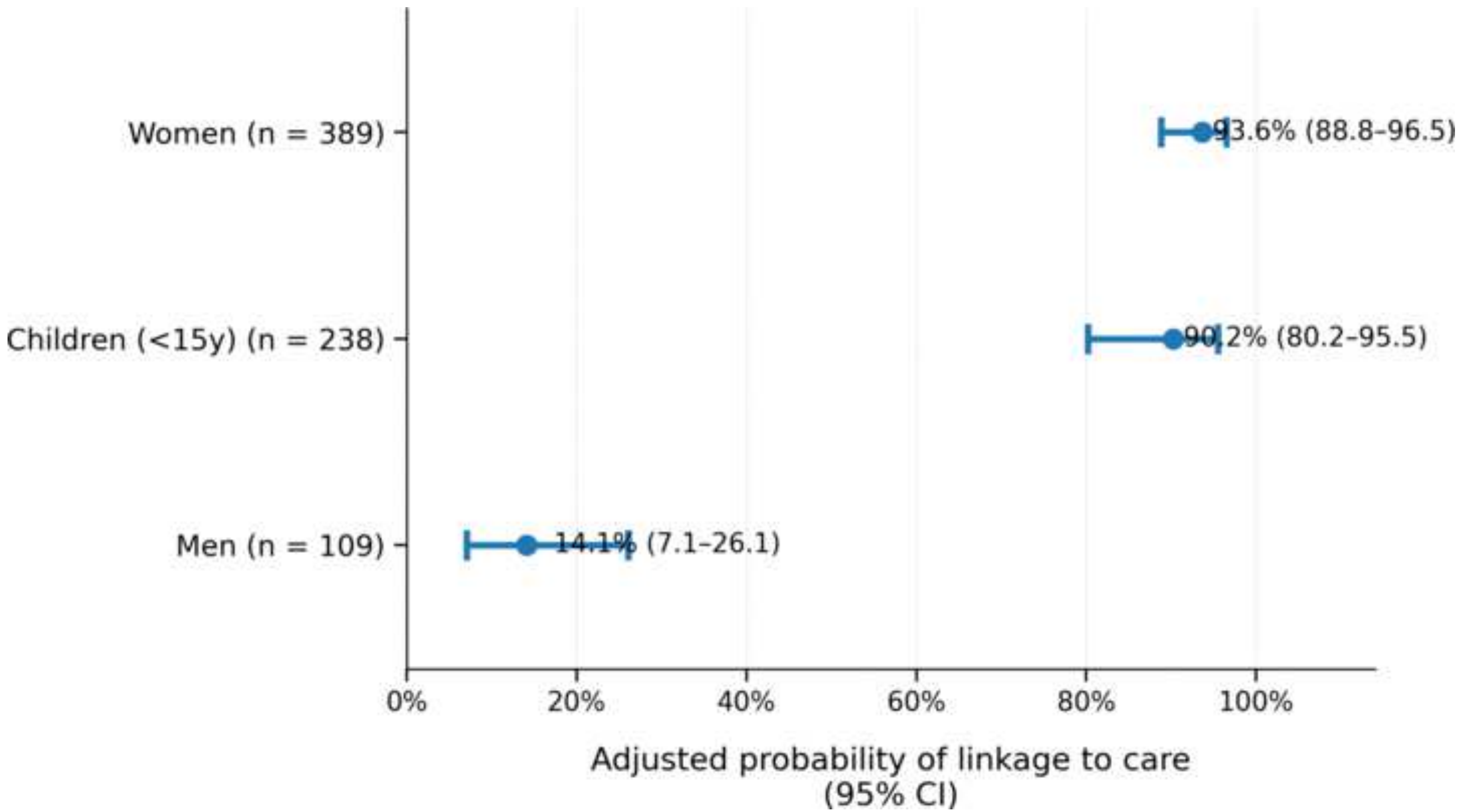

Click here to access/download
Supporting Information
S5_Table.docx

Click here to access/download
Supporting Information
S6_Table.docx

Click here to access/download
Supporting Information
S1_Fig.tif

Click here to access/download
Supporting Information
S2_Fig.tif

Supporting Information

Click here to access/download
Supporting Information
S7_Fig.tif

Click here to access/download
Supporting Information
S4_Description_review.docx

Click here to access/download
Supporting Information
S4_Description.docx

Click here to access/download
Supporting Information
S8_Data.xlsx

Click here to access/download
Supporting Information
S3_Table.docx

RE-AIM evaluation of the national scale-up of community-based index case HIV testing in Guinea: Successes and Challenges

Soriba Camara*[1], Mory 1 Kourouma[1,2], Castro Gbêmêmali Hounmenou [2,3], Aly Badara Touré[1,4], Emile Faya Bongono [2,5]; Sidikiba Sidibé [1], Tamba Mina Millimouno[1,4], Abdoulaye Sow[1], Alioune Camara [1], Alexandre Delamou [1,4].

[1] Department of Public Health, Faculty of Health Sciences and Techniques, Gamal Abdel Nasser University of Conakry, Guinea

[2] Guinea Infectious Disease Research and Training Center, Gamal Abdel Nasser University of Conakry

[3] University of Labé, Department of Computer Science, Labé, Guinea

[4] Africa Center of Excellence for Prevention and Control of Communicable Diseases , Gamal Abdel Nasser University of Conakry, Guinea

[5] Department of Veterinary Medicine, Chair of Infectious and Parasitic Diseases, Higher Institute of Science and Veterinary Medicine of Dalaba, Guinea.

***Corresponding author:** Soriba Camara, Email: soribasante@gmail.com, (SC).

ORCID: https://orcid.org/0009-0009-8951-078X

Abstract

In Guinea ~~Despite notable progress~~, an estimated 36% of people living with HIV ~~in Guinea~~ remain unaware of their status, hindering ~~undermining~~ progress toward the UNAIDS 95-95-95 targets. This study evaluated the effectiveness, implementation fidelity, and sustainability of the community-based index case testing (ICT) strategy using the RE-AIM framework. We conducted a ~~A~~ retrospective mixed-methods evaluation ~~design~~ (2021–2024) ~~was applied~~ across 17 districts supported by 26 community-based organizations (CBOs) using the RE-AIM framework (Reach, Effectiveness, Adoption, Implementation, and Maintenance). Quantitative ~~data were~~ analyses ~~zed using~~ mixed-effects binomial models, segmented regression, and Spearman correlations. In parallel, 28 semi-structured interviews with key stakeholders were thematically analyzed in NVivo, guided by the five RE-AIM dimensions~~: Reach, Effectiveness, Adoption, Implementation, and Maintenance~~. Among 50,609 individuals reached, 8,112 underwent HIV testing, yielding a positivity rate of 8.4% (95% CI: 7.8 to 9.0), with 84.9% (95% CI: 81.9 to 87.6) successfully linked to care. Linkage to care was substantially higher ~~Test acceptance was markedly higher~~ among women (93.6%) and children (90.2%) than among men (14.0%). Adoption expanded from 3 to 17 districts between 2021 and 2023, while cascade performance displayed notable inter-district heterogeneity. Qualitative findings identified strong PMTCT mentorship, community trust, and counseling continuity as key facilitators, contrasted by recurrent challenges including commodity stockouts, financial dependency, and intrahousehold disclosure barriers. The ICT strategy was associated with expanding testing uptake and high linkage to care levels under routine programmatic conditions. Maintenance findings suggest that strengthening institutional integration of CBOs, reinforcing supply chain stability, and improving financing continuity may enhance programme

sustainability. These findings provide context-sensitive operational evidence that may inform the adaptation of differentiated community-based testing strategies in comparable implementation settings.

Keywords: HIV, community-based testing, index case testing, RE-AIM; Guinea.

Introduction

HIV testing remains a cornerstone of the global response, as it determines both the initiation of antiretroviral therapy and the interruption of transmission chains [1] . In 2022, an estimated 39 million people were living with HIV worldwide, with a substantial proportion still unaware of their serostatus, jeopardizing progress toward the UNAIDS 95-95-95 targets set for 2030 [2–5]. Sub-Saharan Africa accounts for nearly two-thirds of this burden and continues to face structural and sociocultural barriers that constrain universal access to testing [2,6] .

In West Africa, diagnostic coverage remains particularly low in rural areas and among young populations at risk [7–12]. To address this gap, community-based approaches have emerged as differentiated and complementary strategies to conventional health services. Among these, index case testing (ICT), involves systematic identification and testing of sexual and household contacts of persons diagnosed with HIV, aiming to improve case detection efficiency and early linkage to care [8,13–18] . Although ICT has demonstrated effectiveness in improving HIV case detection and linkage to care [19–21], evidence on its large-scale implementation and long-term integration within national health systems remains comparatively limited, particularly in low-resource settings [22–25] . Prior studies have documented important operational and contextual challenges, including stigma-related barriers, variability in programme performance, workforce constraints, and supply chain disruptions [26–28]. Understanding ICT performance under routine programme conditions is therefore essential [29,30].In Guinea, national HIV prevalence was estimated at 1.5% in 2018, yet approximately 36% of people living with HIV remain unaware of their status (approximately 43,200 individuals) [31]. Since 2019, the National HIV and Hepatitis Control Program (PNLSH) has piloted an innovative ICT strategy anchored in prevention of mother-to-child transmission

(PMTCT) sites and implemented through community-based organizations (CBOs). Despite its promise, ICT has remained marginal in practice, representing only a small fraction of tests performed outside PMTCT services [32].

Evaluating complex interventions requires frameworks that capture both clinical effects and the conditions under which implementation occurs. ICT qualifies as a complex intervention because it involves multiple interdependent components delivered across facility and community levels, including index-case identification, contact elicitation, community sensitization, HIV testing, referral, and linkage to care. In line with the Medical Research Council guidance on complex interventions, ICT outcomes are influenced not only by these components but also by contextual factors such as stigma, disclosure dynamics, supply-chain reliability, and organizational capacity [33–36] .The RE-AIM framework (Reach, Effectiveness, Adoption, Implementation, Maintenance), widely applied in public health, offers a structured approach to assess population coverage, behavioral and clinical outcomes, organizational uptake, fidelity of implementation, and sustainability [37]. This multidimensional lens is particularly relevant in resource-limited settings, where the success of an intervention depends not only on its efficacy but also on its feasibility, scalability, and institutional anchoring [22–25,34].

Against this background, the present study applied the RE-AIM framework to evaluate an index case HIV testing program implemented in Guinea. The objective was to generate robust evidence on its effectiveness, adoption, and implementation processes, while identifying contextual factors influencing sustainability. Findings may provide insights relevant to ~~other~~ comparable francophone~~s~~ ~~in~~ West African settings.

Methods

Study Design

This study used a mixed-methods implementation evaluation design based on routine programme monitoring data collected between 2021 and 2024. The quantitative component combined serial cross-sectional analyses of programme indicators, time-series approaches to assess inter-annual variability and trends, and longitudinal descriptive analyses to examine organizational continuity of CBOs ~~OCBs~~ during the implementation period. Quantitative data were analyzed from April 20 to May 18, 2025, while qualitative interviews and document reviews were conducted between December 16, 2023, and January 5, 2024. The evaluation focused on a community-based HIV ICT programme implemented in Guinea between 2021 and 2024, under the study protocol approved by the National Ethics Committee for Health Research (CNERS, Ref. No. 060/CNERS/23).

The implementation teams consisted of staff from the National HIV and Hepatitis Control Programme (PNLSH), Fraternité Médicale Guinée (FMG) as Global Fund sub-recipient, and community-based organizations (CBOs) and PMTCT sites delivering ICT activities. These actors were responsible for program planning, service delivery, and routine reporting. In contrast, data collection and analysis were conducted by an independent research team based at the University Gamal Abdel Nasser of Conakry, who had no operational responsibility in program implementation. These researchers designed the evaluation protocol, developed data collection tools, conducted interviews, and performed quantitative and qualitative analyses. Implementers did not participate in statistical modelling or interpretation of the findings.

The RE-AIM framework (Reach, Effectiveness, Adoption, Implementation, Maintenance) guided the evaluation, offering a multidimensional lens to assess both clinical effectiveness and implementation fidelity [37].

Study Population and Sites

The study was conducted in the 17 districts in which the community-based index case testing (ICT) program had been formally implemented during the study period. These districts were not purposively selected; rather, they reflected the progressive national rollout of the ICT strategy, which began with a pilot phase in three districts (Boké, Siguiri and N'Zérékoré) in 2021 and was subsequently expanded to 17 districts by 2023 under the coordination of the National HIV and Hepatitis Control Programme. District inclusion was therefore determined by programme implementation status, the presence of PMTCT sites involved in index-case identification, and the operation of designated CBOs within the Global Fund supported platform (S1 Fig)**.**

The target population consisted of HIV-positive women followed in PMTCT services in these districts and their sexual and household contacts identified through the ICT. Eligible participants for qualitative interviews were adults aged 18 years or older who had direct experience with the community HIV testing strategy, either as beneficiaries or implementers. Children and adolescents were not recruited for the qualitative component of the study ~~Eligible participants for qualitative interviews were adults (≥16 years) who had direct experience with the community HIV testing strategy, either as beneficiaries or implementers~~. The organizational units comprised ~~of~~ 26 community-based organizations (CBOs) operating in the participating districts. Participants were approached through CBO focal points and invited individually after being informed about the study's objectives and confidentiality procedures. Interviews were conducted in French or local languages (Susu, Malinké, Fula, Kpelle, and Guerzé), depending on

participant preference. Exclusion criteria included individuals residing outside intervention areas, those not exposed to the community-based strategy, and those who did not provide informed consent.

Data Sources and Collection

Four complementary data sources were mobilized. Programmatic data consolidated from monthly reports served as the primary basis for quantitative analysis. Routine programmatic data were produced within the national HIV program and reported by community-based organizations (CBOs) to the Global Fund sub-recipient Fraternité Médicale Guinée. These aggregated datasets included cascade indicators such as index cases identified in PMTCT sites, contacts elicited, numbers referred and tested for HIV, HIV-positive diagnoses, linkage to care, and supervision activities. These reports were completed by CBO focal persons and validated by district supervisors. In contrast, KoboCollect was used exclusively for research extraction of individual-level variables not available in ~~not a~~ routine reports ~~ing system~~,~~. It was used exclusively for the research component to extract individual-level and contextual information from CBO registers and verified documents during site visits. KoboCollect captured variables unavailable in routine systems,~~ including age, sex, relationship to the index case, acceptance or refusal of HIV testing and reasons provided, place of testing, linkage outcomes, and reported operational barriers such as commodity stock-outs. KoboCollect forms were completed by trained research assistants who were independent from implementation teams. Programmatic documents (protocols, guidelines, supervision reports) were also reviewed to contextualize implementation processes and support data triangulation. Children aged <15 years were represented only in the routine programme records used for secondary quantitative analysis. They were not contacted, recruited, or interviewed by the research team, and no research-

specific procedures were conducted with them. The quantitative dataset contained de-identified routine programme information, including age group, sex, HIV testing, and linkage indicators.

Semi-structured interviews were conducted with 28 purposively selected participants representing key actor groups involved in or affected by the intervention: (i) national-level program coordinators (PNLSH and Fraternité Médicale Guinée), (ii) health-facility personnel from PMTCT sites, (iii) staff of CBOs (coordinators, supervisors and lay counsellors), and (iv) index cases and household members. These categories contributed respectively to policy-level perspectives, facility-community interface, operational delivery in communities, and user experience and acceptability. All individuals participating in interviews were recruited specifically for research purposes and provided written informed consent prior to data collection. Recruitment continued until thematic saturation was reached. Quantitative analyses relied on de-identified aggregated programmatic data routinely collected within the national HIV programme. Individuals who reached through community awareness, counselling, and testing activities were beneficiaries of routine service delivery and were not enrolled as research participants. Consequently, individual informed consent was not required for secondary analysis of anonymized routine data, consistent with the ethical approval obtained. All programmatic and interview data were anonymized prior to analysis, with no identifying information retained. Data were securely stored on password-protected devices accessible only to the research team. Signed consent forms are archived at the principal investigator’s institution. Interviews conducted in local languages were transcribed and translated into French before coding and analysis.

Description of the Intervention

The intervention was implemented in three phases (Boké, Siguri and N'Zérékoré), reflecting a progressive scale-up of community-based HIV index case testing in Guinea.

Phase 1 (2021) launched the pilot with five CBOs across three districts, focusing on establishing operational protocols, building initial capacity, and testing feasibility. At this stage, PMTCT sites identified index cases from clinical registers and shared lists of partners and household contacts with CBOs. These contacts were approached for sensitization and HIV testing, conducted either at home or in community settings. Individuals who tested positive were referred to PMTCT sites or designated hospitals for confirmatory testing and treatment initiation. **Phase 2 (2022) Adjustment** to 26 CBOs across 17 districts, with emphasis on training and operational rollout. Fraternité Médicale Guinée (FMG), the national sub-recipient of Global Fund grants, coordinated capacity-building, supervision, and resource management. PMTCT sites ensured clinical quality assurance, while CBOs provided community-based continuity through psychosocial support, appointment reminders, and follow-up visits.

Phase 3 (from 2023 onward): corresponded to the geographic expansion and operational consolidation of the intervention. During this phase, the number of participating districts and CBOs increased substantially, while programme activities continued under routine implementation conditions. Referral, testing, and follow-up mechanisms remained anchored within the established PMTCT– community interface (S2 Fig).

CBOs delivered a standardized psychosocial support package, including post-test counselling (emotional support and stigma coping), assistance with voluntary disclosure of HIV status when requested, counselling on ART initiation and adherence, accompaniment to PMTCT/ART services, and linkage to peer and community support groups. Appointment reminders were provided through a dual mechanism combining

telephone calls/SMS where feasible and home visits when clients could not be contacted by phone. Reminders were triggered by missed appointments recorded in PMTCT registers according to national follow-up schedules (R1 = one month, R3 = three months, R6 = six months), with reminder actions initiated within seven days after a missed visit. Follow-up visits were conducted monthly as part of routine support and more frequently for clients at risk of loss to follow-up, those who had not disclosed their HIV status, or newly diagnosed household contacts. Visits occurred either at home, particularly when confidentiality, disclosure support, or household testing were needed, or in clinics during scheduled PMTCT/ART appointments. Priority subgroups included: (i) women lost or at risk of loss to follow-up, (ii) partners not yet tested, (iii) HIV-positive contacts not yet on ART, and (iv) women requesting assistance with disclosure.

Operationalization of the RE-AIM Framework

The RE-AIM framework guided the evaluation by defining operational indicators for each of its five dimensions and specifying the variables analyzed.

Reach was measured by the total number of individuals reached through awareness activities, those who received post-identification counseling, those referred to HIV testing services, and those who accepted testing. Sociodemographic characteristics (age, sex) were used as explanatory variables to describe coverage and beneficiary profiles.

Effectiveness was assessed using analytically distinct conversion and outcome indicators across the HIV testing and care cascade. These included: (i) referral for HIV testing among counselled individuals; (ii) HIV test uptake among those referred for testing; (iii) linkage to care among individuals identified as HIV-positive; and (iv) ART initiation among those referred or linked to care. Whereas Reach captured the absolute number of individuals progressing through the pre-diagnosis cascade, Effectiveness reflected the corresponding conversion proportions and downstream care outcomes. The subgroup analysis presented in Fig 2 specifically examined post-diagnosis referral/linkage to care among HIV-positive individuals by age-sex category.

~~through the acceptance rate among referred individuals, the referral rate of those testing positive to healthcare facilities, and the treatment initiation rate among those referred. Analyses included individual-level covariates (sex, age), contextual variables (descriptive subgroup exposure indicators), and temporal factors (calendar year, with quarterly aggregation applied for implementation-intensity indicators)~~. **Adoption** was evaluated by the number and proportion of districts and CBOs that implemented the intervention. Geographic expansion was classified under the Adoption dimension because it reflected the progressive engagement of organiz~~s~~ational implementation units (districts and CBOs). Reach indicators were defined at the individual level, capturing the population effectively exposed to intervention activities.

Implementation indicators included intensity (median number of HIV testing sessions per CBO per quarter), inter-annual stability (coefficients of variation for key indicators), and quality (treatment initiation rate), as well as correlations across cascade steps. Correlation analyses were conducted using aggregated cascade indicators structured as a region × year panel dataset. Analyses examined the temporal distribution of

sessions, inter-CBO performance, and organizational adaptations. **Maintenance** was assessed through the number of CBOs remaining active at the end of the study period, completeness of the reporting system, and detection of supply chain interruptions. Variables included funding trends, institutional integration, and established monitoring and supervision mechanisms (S3 Table).

Data Analysis

Quantitative analyses were conducted at two complementary levels of observation. Because routine monitoring data were recorded at multiple operational levels, different units of analysis were used depending on the analytical objective: individual-level observations for regression models, CBO × year aggregates for organizational performance analyses, and region × year aggregates for cascade correlation analyses. Individual-level analyses were performed to assess determinants of HIV test acceptance, referral, and treatment initiation. Mixed-effects binomial logistic regression models were applied to account for the hierarchical data structure (individuals nested within community-based organizations, CBOs). Fixed effects included sex, age group, calendar year, and intervention intensity, while a random intercept at the CBO level accounted for clustering [43]. For the subgroup analysis presented in Fig 2, a separate grouped binomial mixed-effects logistic regression model was fitted to estimate linkage to care among individuals recorded as HIV-positive. The outcome was specified as the number linked to care versus the number not linked among HIV-positive individuals. Age-sex category was included as a fixed effect, and a random intercept at the CBO level accounted for clustering. Four observations in which the recorded number linked to care exceeded the number of HIV-positive individuals were excluded as internally inconsistent. Marginal predicted probabilities and 95% confidence intervals were derived from the fitted model using estimated marginal means. Organizational-level

analyses were conducted using aggregated programmatic indicators. For longitudinal analyses of referral and linkage to care outcomes, the unit of analysis was the aggregated CBO × year observation. Repeated annual measurements were modelled using mixed-effects regression. Marginal predicted probabilities were derived from the fitted models as estimated marginal means, representing adjusted model-based probabilities rather than crude observed proportions. Ninety-five percent confidence intervals (95% CI) were calculated using the model-based standard errors. Model diagnostics and technical outputs (AIC, BIC, inter-CBO variance and dispersion parameters) are presented in the Results section and Supporting Information (S4). Associations between cascade activity volumes were assessed using Spearman's rank correlation coefficients [38–40]. The analyses were conducted on aggregated programmatic indicators derived from routine monitoring data and structured as a region × year panel dataset (2021–2024). Variables consisted of aggregated count and proportion measures for sensitization, counseling, testing, HIV positivity, and linkage to care. Given the skewed, non-normal distribution and heteroscedasticity of these indicators, Spearman's nonparametric method was selected. The unit of analysis was the aggregated region × year observation. Correlation strength was interpreted according to predefined thresholds (Table 3). Implementation intensity was operationalized as the median number of testing sessions conducted per CBO per reporting period, calculated from routine monitoring data (S6 Table). Mean values presented in summary tables (Tables 1 and 2) are provided for descriptive purposes only due to the skewed distribution of activity volumes. All analyses were performed in R version 4.3.1.

Qualitative analysis followed a hybrid deductive–inductive thematic approach supported by NVivo [41]. The RE-AIM framework informed the initial coding structure (deductive

component), ensuring conceptual alignment with evaluation dimensions. Within each RE-AIM domain, inductive coding was applied to identify context-specific subthemes and operational factors emerging from participant narratives [42,43], The coding framework was iteratively refined through independent double coding and consensus meetings, enhancing analytic reliability (S5 Table). Themes synthesized in Table 4 reflect this structured analytic process.

Integration of quantitative and qualitative strands followed an explanatory sequential design: quantitative findings guided the focus of interviews and interpretation, enabling exploration of perceived causes of observed trends (e.g., supply shortages, stigma, organizational bottlenecks).

Ethical Considerations

The study protocol, including the secondary analysis of de-identified routine programme data, was ~~This doctoral research is part of a national implementation study on community-based HIV testing in Guinea,~~ approved by the National Ethics Committee for Health Research (CNERS, Ref. No. 060/CNERS/23). The study did not involve any clinical or interventional component but consisted of cross-sectional surveys and semi-structured interviews with participants who had experience with the community HIV testing approach. Written informed consent applied exclusively to participants recruited for the qualitative interviews. All interview participants were adults aged 18 years or older; no children or adolescents were recruited for interviews or any other research-specific data collection. All participants were informed about the study objectives, the voluntary nature of their participation, and the confidentiality measures in place. Children aged <15 years were included only through de-identified routine programme records used for secondary quantitative analysis. They were beneficiaries of standard HIV programme services and were not enrolled or contacted as research participants.

Formatted: Font: Not Bold, Not Highlight

Formatted: Font: Bold

Formatted: Font: Bold, Not Highlight

Formatted: Font: (Default) Arial, Not Bold

Consequently, no additional research-specific parental permission or child assent was sought for the secondary analysis of these records, as covered by the CNERS-approved study protocol. Any consent procedures associated with routine HIV testing and care were governed by national programme procedures and were separate from consent for participation in this research.

~~Informed consent was obtained exclusively from participants involved in the qualitative interviews. These individuals were recruited specifically for research purposes and provided written consent prior to participation~~. For adult interview ~~illiterate~~ participants, who were unable to read, ~~interviews and the consent process were conducted in local languages~~, the information sheet was read aloud in their preferred language, and ~~verbal consent~~ was documented in the presence of an independent witness. All datasets were de-identified before analysis, and no personal identifiers were retained. ~~Quantitative analyses were based on de-identified aggregated programmatic data routinely collected within the national HIV programme. The individuals reached through community awareness and service delivery activities were beneficiaries of standard programme implementation and were not enrolled as research participants. In accordance with national ethical guidelines, individual informed consent was therefore not required for secondary analysis of anonymized routine data.~~

Formatted: Font: Not Bold

Formatted: Font: Not Bold, Not Highlight

Formatted: Not Highlight

Formatted: Not Highlight

Formatted: Font: Not Bold, Not Highlight

Formatted: Not Highlight

Formatted: Font: Not Bold

Formatted: Font: Not Bold

Results

Reach

A total of 50,609 individuals were reached through community awareness and mobilization activities across the intervention districts. Among them, 26,025 (51.4%, 95% CI: 51.0–51.9) received individualized post-identification risk counseling. Of those counseled, 14,169 (54.4%, 95% CI: 53.8 to 55.0) were referred for HIV testing. Among individuals referred, 8,112 (57.3%, 95% CI: 56.3 to 58.3) completed an HIV test. The overall HIV positivity rate among those tested was 8.4% (95% CI: 7.8 to 9.0). Of individuals diagnosed with HIV, 577 (84.9%, 95% CI: 81.9 to 87.6) were successfully linked to HIV care and treatment services. Progression along the cascade is summarized in Fig 1.

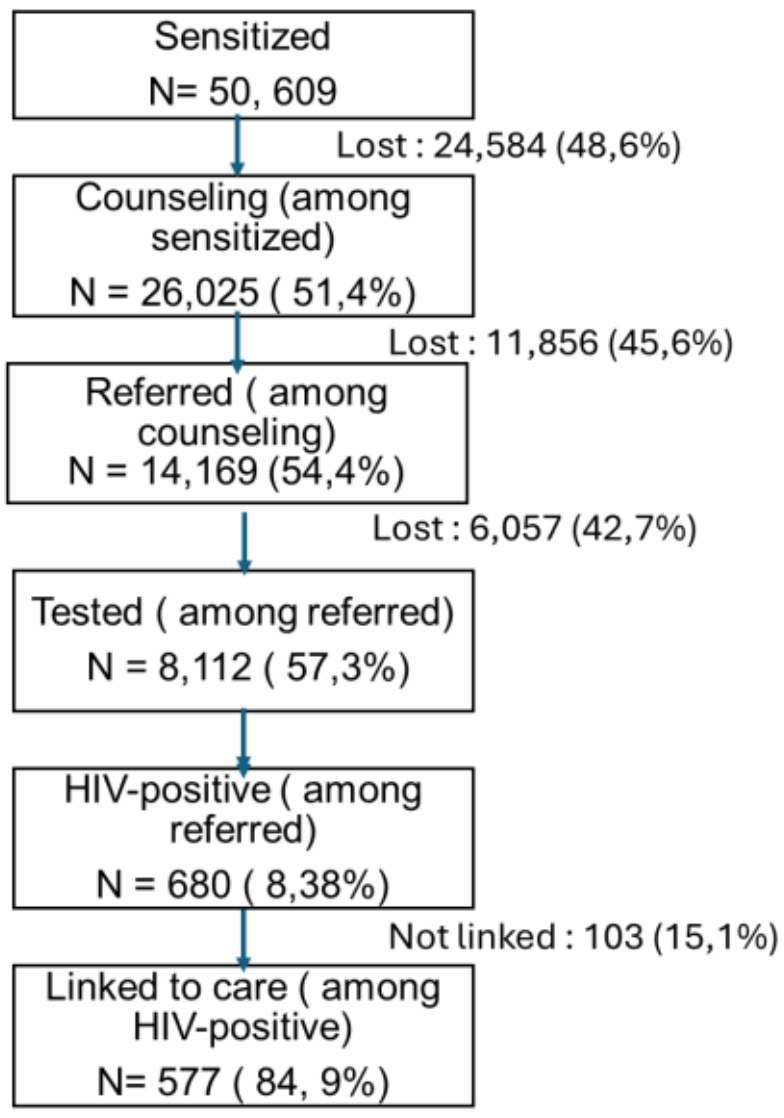

Fig 1: Community-based HIV testing cascade in Guinea, 2021–2024.
Progression of individuals from community outreach to HIV testing and linkage to care, showing cumulative attrition across cascade stages and ~~strong performance~~ the proportion linked to care after ~~in post-~~ HIV diagnosis ~~linkage~~.

Effectiveness

Effectiveness was assessed using four analytically distinct cascade indicators: testing referral among counselled individuals, test uptake among those referred for testing, linkage to care among individuals diagnosed HIV-positive, and ART initiation among those referred or linked to care~~Effectiveness was assessed using three predefined indicators: (i) HIV test acceptance among referred individuals, (ii) referral of individuals diagnosed with HIV to health facilities, and (iii) ART initiation among those referred~~. These indicators were analysed~~s~~ across implementation phases (Table 1 and Fig 2). Across phases, variation was observed for all three indicators. In 2021 (The launch phase), 53.2% of counseled individuals were referred ~~al~~ for HIV testing, 90.2% of those referred completed testing (test acceptance) and 73.3% of individuals referred to care initiated ART. In 2022 (adjustment phase), the proportion of counseled individuals referred for testing declined to 46.7%, and test acceptance among referred individuals decreased to 83.7%. In contrast, ART initiation among those referred increased to 94.3%. The expansion phase (2023–2024) showed heterogeneous patterns. The test uptake ~~acceptance~~ value recorded for 2023 (> 1,000%) was identified as a non-interpretable value ~~outlier~~ caused by a denominator reporting error in routine program data, this value ~~and~~ was excluded from summary analyses (Table 1). In 2023, the referral rate among counseled individuals dropped to 3.6%, coinciding with geographic expansion from 3 to 17 districts and from 5 to 26 CBOs. During the same year, routine monitoring data showed increased inter-annual variability in cascade indicators and a discrepancy between recorded referrals and tests performed in a subset of CBOs, as further described under the Maintenance dimension. In 2024,

referral increased to 75.4% and test acceptance reached 62.6%, and ART initiation stabilized at 76.4%.

A separate sSubgroup analysies – examined linkage to care among individuals identified as HIV-positive. The adjusted probability of linkage was 93.6% among women (95% CI: 88.8 to 96.5; n = 389), 90.2% among children aged <15 years (95% CI: 80.2 to 95.5; n = 238), and 14.1% among men (95% CI: 7.1 to 26.1; n = 109). The mixed-effects binomial model accounting for clustering at the CBO level showed markedly lower linkage to care among men (Fig 2). These values represent model-based marginal predicted probabilities rather than crude proportions. The subgroup sample sizes correspond to the total numbers of HIV-positive individuals represented in the grouped binomial observations retained by the fitted model.

focused on HIV test acceptance among referred individuals. Acceptance was significantly high among children (< 15 years) 90.2% (95% CI: 80.1 to 95.5) and adult women (≥ 16 years) 93.6% (95% CI: 88.7 to 96.5) compared with adult men 14.0% (95% CI: 7.0 to 25.9). Mixed-effects binomial models accounting for clustering at the CBO level confirmed these differences (Fig 2). Marginal predicted probabilities represent adjusted model-based estimates rather than crude proportions. Inter-organizational variability remained measurable, as indicated by the random intercept variance at the CBO level.

Formatted: Highlight

Adoption and Maintenance dimensions provide complementary context to interpret effectiveness patterns. Adoption describes the scale-up of organizational and geographic coverage, while Maintenance assesses the continuity and stability of implementation conditions influencing cascade progression.

Table 1: Effectiveness indicators of community-based HIV index testing across implementation phases in Guinea (2021–2024)

Phase		Mean testing referral rate	Mean test~~ing~~ uptake ~~acceptance~~	Mean ART ~~treatment~~ Initiation
Launch (2021)		53.2%	90.2%	73.3%
Adjustment (2022)		46.7%	83.7%	94.3%
Expansion	(2023)	3.6%	NA†	74.3%
	(2024)	75.4%	62.6%	76.4%

NA† 2023 test~~ing~~ uptake- ~~acceptance~~ value excluded due to a denominator reporting error producing a non-interpretable rate >1000%. Testing-referral rate was calculated among counselled individuals; test uptake was calculated among individuals referred for HIV testing. These indicators are distinct from the post-diagnosis referral/linkage outcome presented in Fig 2.

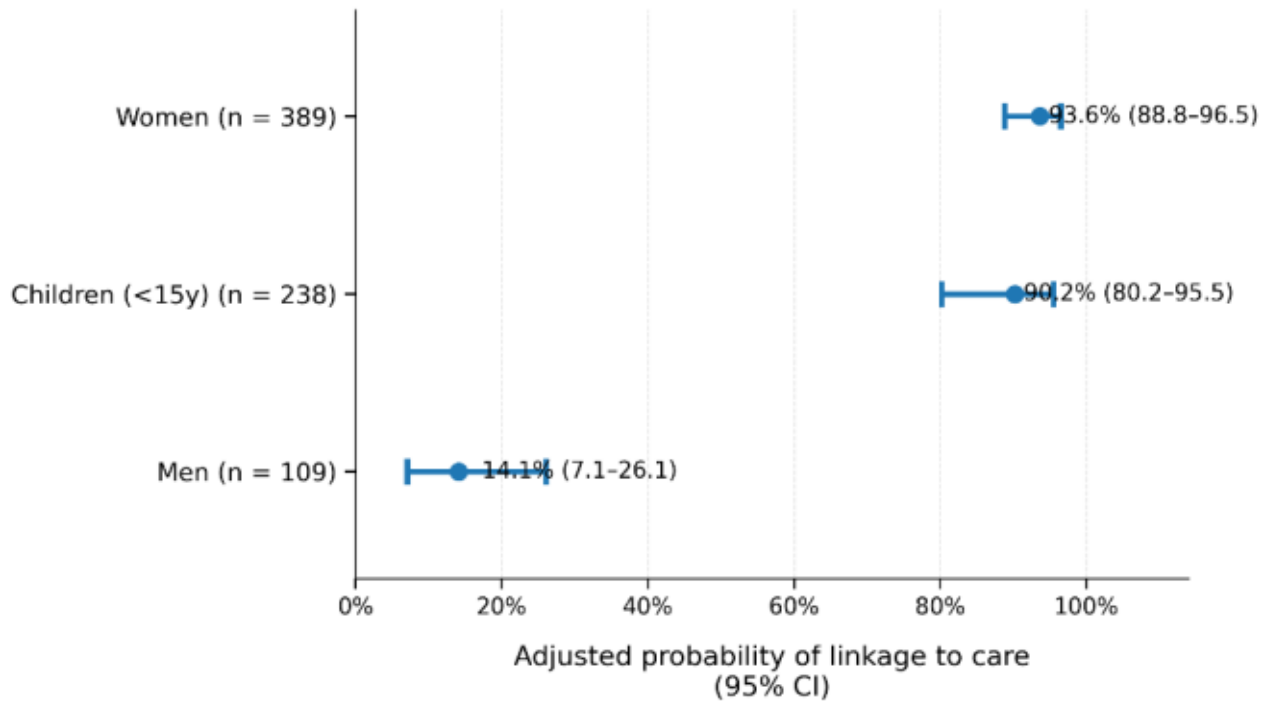

Fig 2 : Marginal predicted probabilities of linkage to care among HIV-positive individuals ~~HIV test acceptance~~ by age group and sex. Adjusted probabilities were derived from a mixed-effects binomial logistic regression model with age-sex category as a fixed effect and a random intercept at the CBO level. Sample sizes represent the numbers of HIV-positive individuals contributing to the fitted model: women, n = 389; children aged <15 years, n = 238; and men, n = 109. Points represent marginal predicted probabilities and horizontal bars represent 95% confidence intervals. ~~including age group, sex, calendar year and intervention intensity as fixed effects, with a CBO-level random intercept to account for clustering. Estimates are modelbased (estimated marginal means) and do not represent crude observed proportions.~~

Adoption

The intervention was initially implemented in three districts and five community-based organizations (CBOs) in 2021 and was progressively expanded to 17 districts and 26 CBOs by 2023. This scaling-up corresponded to a 340% increase in the number of

Formatted: Left
Formatted: Left
Formatted: Left
Formatted: Justified
Formatted: Font: (Default) Arial, 11 pt
Formatted: Font color: Accent 1

participating districts and a 420% increase in the number of implementing CBOs compared with the 2021 baseline.

At the planning stage in 2020, 26 CBOs across 17 districts had been formally designated as eligible implementers by the national program. By 2023, all 26 eligible CBOs were implementing the intervention, corresponding to 100% organizational adoption among eligible sites. The 340% and 420% increases therefore indicate relative program expansion over time, whereas the 100% value reflects complete adoption among those officially designated as eligible. No eligible CBOs or districts declined participation. Reporting adoption indicators was high, with 98.7% data completeness over the study period (Figure 3).

Longitudinal changes in referral and linkage indicators were assessed using mixed-effects regression models, with calendar year entered as a time variable and a random intercept at the CBO level to account for repeated measurements within organizations. This analysis showed a statistically significant average annual decline in referral and linkage to care, estimated at −2.54% per year (95% CI: −4.86 to −0.21; $p = 0.033$).

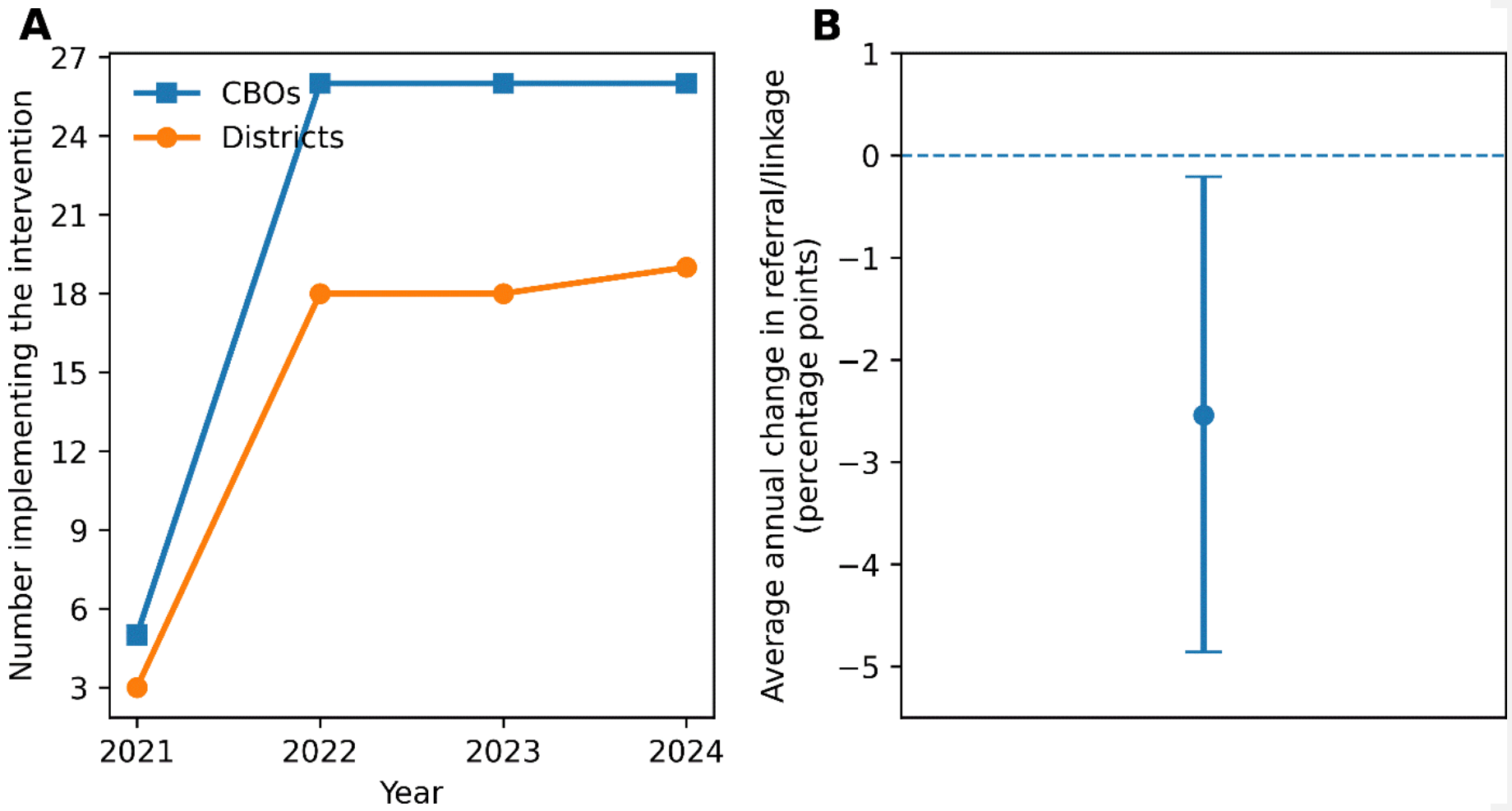

Fig 3 :. Adoption and longitudinal change in referral/linkage indicators. (A) Scale-up of community-based index testing, showing the number of districts and CBOs implementing the intervention (2021–2023). (B) Average annual change in referral/linkage estimated from mixed-effects regression with calendar year as time variable and a CBO-level random intercept; points show model-based estimates and bars 95% confidence intervals (percentage-point change).

Implementation

The implementation unfolded in three operational phases (Table 4, S6 Table): an initial high-intensity phase in 2021, characterized by rapid mobilization and high activity volumes; an adjustment phase in 2022, marked by stabilization of procedures; and an expansion phase in 2023, corresponding to the geographic scale-up of community-based organizations (CBOs).

Interannual stability analysis showed substantial variability in upstream cascade indicators, including sensitization (CV = 0.63), counseling (CV = 0.63), and testing (CV = 0.58). Downstream indicators such as HIV positivity (CV = 1.82) and linkage to care (CV = 1.87) exhibited markedly higher variability (Table 2). Correlation analyses, performed on cascade indicators structured as a region × year panel dataset, showed very strong monotonic associations between sensitization, counseling, and testing ($\rho > 0.90$, $p < 0.001$). The correlation between HIV-positive cases and linkage to care was

also very strong (ρ = 0.946, $p < 0.001$). Moderate correlations were observed between counseling and linkage to care (ρ = 0.561, $p = 0.029$) and between testing and linkage to care (ρ = 0.542, $p = 0.037$), while other associations were weaker and not statistically significant (Table 3).

Table 2: Interannual stability of HIV testing cascade indicators in the community-based programme, Guinea (2021-2024)

Indicator	N° years	Annual mean	Annual SD	CV (2021–2024)	Interpretation
Counseling	4	542.0	341.0	0.63	High variability
Linked to care	4	20.8	38.9	1.87	Very high variability
HIV positive	4	21.5	39.1	1.82	Very high variability
Sensitized	4	531.0	341.0	0.64	High variability
Tested	4	490.0	282.0	0.58	High variability

CV = coefficient of variation (SD/mean). Interpretive thresholds: <0.20 = low; 0.20–0.50 = moderate; 0.50–1.00 = high; >1.00 = very high.

Formatted: Justified
Formatted: Justified
Formatted: Justified
Formatted: Justified
Formatted: Justified

Table 3: Spearman correlation coefficients (ρ) between HIV testing cascade indicators (region × year panel, 2021–2024)

Cascade relationship (Variable 1 → Variable 2)	ρ (Spearman)	*p*-value	Strength	Expected programmatic linkage
Awareness → Counseling	0.929	<0.001	Very strong	Conversion from sensitization to counseling
Counseling → Testing	0.945	<0.001	Very strong	Expected stepwise progression
Awareness → Testing	0.917	<0.001	Very strong	Overall upstream coherence
HIV positive → Linked to care	0.946	<0.001	Very strong	Post-diagnosis referral mechanism
Awareness → Linked to care	0.592	0.020	Moderate	Indirect association
Counseling → Linked to care	0.561	0.029	Moderate	Indirect linkage via testing
Testing → Linked to care	0.542	0.037	Moderate	Reflects conversion to treatment
Awareness → HIV positive	0.492	0.063	Weak–moderate	Not significant
Counseling → HIV positive	0.466	0.080	Weak–moderate	Not significant
Testing → HIV positive	0.495	0.061	Weak–moderate	Not significant

Formatted: Justified

Spearman correlation coefficient. Interpretive thresholds: < 0.20 = very weak; 0.20–0.39 = weak; 0.40–0.59 = moderate; 0.60–0.79 = strong; ≥ 0.80 = very strong. Statistical significance set at $p < 0.05$.

Maintenance

Between 2022 and 2024 the community-based network remained stable, with 26 active CBOs and a median operational tenure of three years. Programmatic records did not directly document the availability of testing commodities at the CBO level. Nevertheless, several indirect signals of operational instability were detected. Each year since 2022, approximately 19.2% of CBOs showed a discrepancy between the number of recorded referrals and the number of tests performed. Likewise, 7.7% to 11.5% of CBOs exhibited atypical intra-annual fluctuations in the tested-to-referred ratio. Taken together, these

findings indicate that “maintenance” was achieved in terms of organizational presence and program footprint, but not uniformly in terms of routine operational performance. These quantitative patterns were further contextualized by qualitative findings, which also informed other RE-AIM dimensions (Reach, Effectiveness, and Implementation), but were particularly critical for interpreting sustainability-related instability signals. Multiple participants reported structural and organizational constraints affecting service continuity, notably irregular commodity supplies and dependence on external funding. Taken together, these findings indicate that “maintenance” was achieved in terms of organizational presence and program footprint, but not uniformly in terms of routine operational performance**.** The divergence between standardized databases and implementers’ perceptions underscores the need for systematic triangulation of information sources, improved data quality systems, and stronger supply-chain monitoring to accurately assess the stability and sustainability of community-based interventions (Table 4).

Table 4: Summary of quantitative and qualitative findings related to Maintenance and implementation conditions, Guinea, 2021–2024

**Element**	**Indicator / Quantitative evidence**	**Qualitative corroboration**	**Illustrative verbatims**	**Suggested actions**
**Organizational stability**	26 active CBOs; median tenure = 3 years (2022–2024)	Implementers highlighted sustained CBO engagement and local legitimacy	*“The advantage we had was selecting NGOs that were already active in the*	Formalize CBO roles in district health plans; establish MOUs and routine supervision schedules

			field." (Coordinator 1)	
**Data coherence**	~19.2% of CBOs annually showed referral-to-test discrepancies; 7.7–11.5% presented atypical intra-annual tested/referred fluctuations	Staff reported mismatches between program databases and field realities	*"At the beginning we did not have a well-established strategy; we had to adapt the health centre tools."* (Coordinator 1)	Strengthen routine data validation, introduce triangulation checks, and train CBO data focal points
**Commodity security**	No official programmatic stockout records; implementers nevertheless reported intermittent supply interruptions	Several implementers cited irregular HIV test kit supply as affecting continuity	*"The biggest problem we had was with inputs, since some health centre managers were reluctant to supply CBOs."*	Institutionalize supply chain reporting at CBO level; establish buffer stocks and rapid resupply procedures
**Financial sustainability**	Ongoing dependence on external funding reported	Concerns raised about continuity if external support declines	*"After CBO training in 2020, activities only began in 2021 because we had not received funding."*	Develop phased domestic financing plans; diversify funding and formalize cost-sharing mechanisms

**Human resources and supervision**	Reports of variable training quality, high turnover, and limited supervision	Implementers highlighted gaps in refresher training and logistical support	*"There are many changes among community counselors; sometimes allowances are delayed, and newcomers need training."*	Implement regular refresher training, standardized supervision tools, and retention incentives
**Confidentiality and stigma**	Identified as a constraint for linkage and test uptake	Fear of disclosure within households frequently reported	*"Only my children know I am sick, because if other family members knew, I would be rejected."* (Index case 6)	Strengthen confidentiality protocols, counselor training, and community stigma-reduction activities
**Community ownership**	Continued involvement of local leaders and CBOs documented	Reports described local leaders organizing awareness sessions and enhancing legitimacy	*"When we enter a community, before starting sensitization, we approach the local leaders; they give us the green light and their support."* (CBO 11)	Sustain and formalize community leadership roles; involve local leaders in performance reviews

Formatted: Left

Mechanisms of impact of the intervention

The five RE-AIM dimensions describe multidimensional ~~of~~ programme dynamics. Expansion of coverage (Reach) was accompanied by household exposure to HIV testing activities, whereas Effectiveness indicators displayed heterogeneous ~~patterns.~~ patterns. Linkage to care was substantially higher among women and children than among men, revealing a marked sex-related gap in engagement with the care pathway following an HIV-positive result.~~Test acceptance was high among women and children but remained comparatively low among men, contributing to variability across the cascade steps~~. Across the RE-AIM dimensions, periods of program expansion, reflected by increased organizational adoption and geographic coverage, were temporally associated with higher activity volumes, while also coinciding with greater variability in effectiveness and maintenance indicators. Implementation patterns evolved through phases of intensity, adjustment, and expansion, with performance varying in relation to session intensity and inter-CBO stability. Qualitative analyses suggested several operational factors perceived to support program functioning, including community counseling, PMTCT mentorship, and longitudinal follow-up, that enhanced trust and continuity of care. They also identified recurrent structural and operational constraints, notably, commodity stockouts, reliance on external funding, and difficulties with intra-household disclosure. These factors were perceived by participants as influencing the conversion of sensitization into testing uptake and linkage to care. Integrated quantitative – qualitative triangulation helped identify patterns not fully captured by routine indicators. Taken together, these observations suggest that sustaining program performance may depend on balancing coverage expansion with operational stability, including strengthening supply chain reliability and institutional support mechanisms (S7 Fig).

Discussion

This study, guided by the RE-AIM framework, evaluated the implementation and effectiveness of an index-assisted community HIV testing strategy in Guinea from 2021 to 2024. The intervention reached more than 50,000 individuals, but performance varied across the cascade, including marked age- and sex-related differences in linkage to care. ~~and achieved high testing acceptance, yet performance varied across the cascade, particularly for referral to care and treatment initiation~~. Three principal findings frame the discussion: (1) variable effectiveness across implementation phases and population subgroups~~strong initial effectiveness followed by a relative decline during expansion, with marked inter-site heterogeneity~~; (2) a three-phase implementation trajectory, initial intensity, organizational adjustment, and territorial expansion; and (3) fragile institutional sustainability.

The index approach documented substantial community-based testing activity and linkage to care among individuals diagnosed during routine implementation. ~~The index approach substantially expanded testing access and accelerated treatment initiation among those diagnosed.~~The markedly lower adjusted probability of linkage among men represents an important implementation gap. Comparable studies in African settings have documented lower engagement of men in HIV testing and care pathways than women, reflecting gender-related differences in healthcare use, stigma, disclosure concerns, and service accessibility [44,45] . In the present study, qualitative findings concerning stigma and difficulties with intra-household disclosure provide plausible contextual explanations. However, the analysis did not directly collect male-specific reasons for non-referral, and the observed difference should not be interpreted as direct evidence that men refused to accept their HIV status. Rather, it indicates lower male

Formatted: Not Highlight

Formatted: Highlight

Formatted: Not Highlight

engagement with the post-diagnosis care pathway, which may reflect a combination of individual, household, community, and service-level barriers.

Lower male engagement may reduce opportunities for partner support and complicate voluntary and safe disclosure within households. Nevertheless, this evaluation did not assess women's retention according to their partners' referral status or measure intimate-partner or gender-based violence outcomes; no causal relationship can therefore be inferred. Male-responsive strategies, including trained male peer navigators or trusted community champions, confidential counselling, flexible service-delivery modalities, and supported referral, should be prospectively evaluated. Such approaches should also incorporate voluntary disclosure options, confidential assessment of disclosure-related safety, and appropriate referral pathways for individuals at risk of violence [46–48] .~~Test acceptance was markedly higher among women and children, which is plausibly attributable to the strong community anchoring of CBOs and their formal collaboration with PMTCT sites; this articulation appears to have promoted family engagement and smoothed linkages between community screening and clinical care. These findings align with evidence from Senegal and Côte d'Ivoire showing that targeted intrafamilial testing improves early case detection and rapid linkage to treatment~~ . Nevertheless, pronounced heterogeneity in referral rates highlights persistent structural constraints. As reported in Burkina Faso, irregular availability of commodities and stigma-related barriers limited cascade performance [49,50]. Qualitative interviews indicated that difficulties in disclosing serostatus to partners are an important deterrent to care-seeking, thereby reducing the intervention's net impact. In our study, the sharp decline in referral observed in 2023 occurred during the rapid expansion phase and may reflect a combination of operational instability and reporting constraints. Implementers reported turnover among community counsellors,

requiring training and a transition period to rebuild trust with index cases and households, as well as recurrent interruptions or irregular supply of HIV testing commodities affecting community testing and referral pathways. In addition, 2023 coincided with preparation of the Global Fund funding request for 2024–2026, which may have temporarily affected routine supervision, reporting and data validation processes. Collectively, these observations suggest~~confirm~~ that, despite favourable implementation outcomes in several cascade steps, index testing remains vulnerable to local resource dynamics, sociocultural norms, and coordination gaps, as documented in other West African studies [51–59]. However, the relative contribution of these factors cannot be quantified with precision using routine programme data al one.

The three-phase implementation pattern reflects organizational learning during scale-up [60–63] . The expansion phase may have introduced a dilution effect: as the programme broadened beyond initial high-priority networks, diagnostic yield and cascade conversion may have become more variable~~The observed negative correlation between effectiveness and acceptability suggests a dilution effect: as the program broadened beyond high-risk networks, it reached larger populations with lower HIV prevalence, mechanically reducing the diagnostic yield~~. This pattern, reported in West and Southern Africa, indicates that territorial expansion must be accompanied by adaptive targeting and revised operational criteria to preserve effectiveness while maintaining access [64–66].

Sustainability of the community testing model in Guinea depends on two interdependent conditions: the long-term viability of external financing amid declining international aid, and formal integration of the approach within national health systems. This dual dependence creates structural fragility that has undermined community interventions elsewhere in sub-Saharan Africa when external funding ceased [67,68]. In

Guinea, the lack of institutionalized supply mechanisms for CBOs exacerbated vulnerability: intermittent commodity shortages were reported by several implementers, despite no documented “stockout” events in routine program databases. This discrepancy exposes limitations of standard monitoring indicators and underscores the value of systematic triangulation between quantitative records and qualitative inquiry to detect operational interruptions. The RE-AIM framework highlighted programme expansion, reflected by increased organizational adoption and geographic coverage, coincided with higher reported activity volumes, while also revealing variability in effectiveness and maintenance indicators. These multidimensional tensions would likely have remained undetected in evaluations focused solely on biomedical outcomes.

This study has several methodological limitations. Its retrospective design and reliance on routine programmatic data may have introduced reporting and completeness biases. -The study did not directly examine the reasons why HIV-positive men were not linked to care, assess women’s treatment retention according to their partners’ referral status, or measure adverse social outcomes such as unwanted disclosure, intimate-partner violence, or other forms of gender-based violence. The findings therefore identify a substantial male-engagement gap but do not establish its underlying mechanisms or its consequences for female partners’ treatment outcomes. The absence of a contemporaneous comparison group limits causal inference, and residual inter-site variability suggests that unmeasured contextual factors, such as local coordination capacity, supply consistency, and population mobility, may have influenced outcomes. Nevertheless, the study presents notable strengths, including broad geographic coverage across 17 districts, methodological triangulation combining quantitative and qualitative data, and the structured application of the RE-AIM framework to evaluate a national-scale community-based HIV testing strategy.

Policy and programmatic implications follow directly. Findings from the Maintenance dimension indicate that long-term sustainability remains vulnerable due to reliance on external funding, incomplete integration into national budgeting mechanisms, and gaps in commodity supply reporting at the CBO level. Strengthening institutional integration within district health planning processes and national financing frameworks may enhance stability of community-based index testing. Structural integration of community-based organizations into the formal health system can enhance service reach among hard-to-reach and at-risk populations, while reducing attrition along the cascade. Programmes should consider a male-responsive post-diagnosis support package combining trained male peer navigators or trusted community champions, confidential counselling, flexible service hours and referral options, and active accompaniment to HIV care. Because partner engagement and disclosure may raise safety concerns, these approaches should remain voluntary and include confidential assessment of disclosure-related risks and functional referral pathways for psychosocial support and protection. Their acceptability, effectiveness, and potential unintended consequences should be assessed prospectively.-. Financial sustainability requires the progressive incorporation of operational costs into national health budgets and proactive mobilization of domestic co-financing mechanisms to reduce dependence on external donors.

Future research should assess the cost-effectiveness of this model to guide evidence-based resource allocation. Future studies should prospectively evaluate male-responsive and peer-supported strategies using sex-disaggregated outcomes for post-diagnosis referral, linkage to care, ART initiation, and retention. They should document male-specific reasons for engagement or non-engagement, examine whether male partner linkage is associated with women's retention in care, and systematically monitor

unintended social harms, including unwanted disclosure and intimate-partner or gender-based violence. Quasi-experimental studies including comparison groups could strengthen causal inference, while targeted qualitative analyses may identify the most effective operational levers to sustain performance during scale-up. Longitudinal cohort studies are also warranted to evaluate long-term outcomes in treatment adherence and retention.

Conclusion

Overall, the findings illustrate the operational performance of the integrated "*community – clinical index testing model*" in identifying HIV-positive individuals and facilitating linkage to care during the implementation period. However, the markedly lower probability of linkage among men identifies a critical gap in the community-clinical pathway. Programmes should consider and rigorously evaluate male-responsive, peer-supported, and safety-centred approaches to improve men's engagement with HIV care following diagnosis. Consolidation of supply chains, institutional support for community-based organizations, and sustainable financing are critical to ensuring durability. These findings contribute to the evidence based on large-scale implementation of community-based index testing under routine programme conditions, highlighting factors relevant to programme performance and sustainability.

Formatted: Indent: Left: 0"

Data availability statement

All data underlying the findings of this study are provided as Supporting Information. The quantitative analyses were conducted using a de-identified aggregated dataset of quarterly programme monitoring data, included as S8 Data. No individual-level identifiers are included in the shared dataset. These data are sufficient to reproduce the analyses reported in the manuscript. Data sharing is consistent with the ethical approval granted by the National Ethics Committee for Health Research in Guinea and complies with applicable confidentiality requirements.

Acknowledgments

We thank all study participants for sharing their perspectives on community-based HIV testing. We acknowledge the National HIV and Hepatitis Control Program (PNLSH) and Fraternité Médicale Guinée (FMG) for facilitating access to study sites, community-based organizations, and programme records. We also thank the Africa Centre of Excellence for Prevention and Control of Communicable Diseases and Cheikh Anta Diop University of Dakar for their academic and institutional support. We acknowledge the use of AI-assisted tools, including ChatGPT, for language editing. All authors reviewed and approved the final manuscript and take full responsibility for its content.

References

1. UNAIDS. Global AIDS Update : The Path That Ends AIDS. Geneva; 2023. Available: https://www.unaids.org/en/resources/documents/2023/global-aids-update
2. UNAIDS. Global AIDS report : The Urgency of Now: AIDS at a Crossroads. In: AIDS report 2024 [Internet]. 2024. Available: https://www.unaids.org/en/resources/documents/2024/global-aids-update-2024
3. Akullian A, Akulu R, Aliyu G, Anam F, Guichard AC, Ayles H, et al. The HIV response beyond 2030: preparing for decades of sustained HIV epidemic control in eastern and southern Africa. The Lancet. 2024;404: 638–641. doi:10.1016/S0140-6736(24)00980-2
4. Hlongwa M, Moyo E, Dzinamarira T. Approaches for improving linkage to HIV care among HIV self-testing individuals in sub-Saharan Africa. BMJ Glob Health. 2023;8: e012664. doi:10.1136/BMJGH-2023-012664
5. World Health Organization (WHO). Consolidated Guidelines on HIV Prevention, Testing, Treatment, Service Delivery and Monitoring: Recommendations for a Public Health Approach, Geneva. Jul 2025. Available: https://pubmed.ncbi.nlm.nih.gov/34370423/
6. World Health Organization (WHO). HIV and AIDS report. 15 Jul 2025. Available: https://www.who.int/news-room/fact-sheets/detail/hiv-aids
7. Boye S, Kouadio A, Kouvahe AF, Vautier A, Ky-Zerbo O, Rouveau N, et al. Organisation of testing services, structural barriers and facilitators of routine HIV self-testing during sexually transmitted infection consultations: a qualitative study of patients and providers in Abidjan, Côte d'Ivoire. BMC Infect Dis. 2022;22. doi:10.1186/S12879-023-08625-X,
8. Seidu A-A. Using Anderson's Model of Health Service Utilization to Assess the Use of HIV Testing Services by Sexually Active Men in Ghana. Front Public Health. 2020;8: 512. doi:10.3389/fpubh.2020.00512
9. Lakhe NA, Diallo Mbaye K, Sylla K, Ndour CT. HIV screening in men and women in Senegal: coverage and associated factors; analysis of the 2017 demographic and health survey. BMC Infect Dis. 2019;20: 1. doi:10.1186/S12879-019-4717-5
10. Rouveau N, Ky-Zerbo O, Boye S, Fotso AS, d'Elbée M, Maheu-Giroux M, et al. Describing, analysing and understanding the effects of the introduction of HIV self-testing in West Africa through the ATLAS programme in Côte d'Ivoire, Mali and Senegal. BMC Public Health. 2021;21. doi:10.1186/s12889-021-10212-1
11. Morin L, Béhanzin L, Guédou FA, Kêkê RK, Bushman L, Anderson PL, et al. HIV Prevention and Treatment Cascades among Female Sex Workers in Benin, West Africa. Sex Transm Dis. 2021;48: 654–662. doi:10.1097/OLQ.0000000000001399
12. Hatzold K, Dunkley Y. Effects of self-testing on HIV outcomes in west Africa. Lancet HIV. 2024;11: e498–e500. doi:10.1016/S2352-3018(24)00179-6
13. Dalal S, Johnson C, Fonner V, Kennedy CE, Siegfried N, Figueroa C, et al. Improving HIV test uptake and case finding with assisted partner notification services. AIDS. 2017;31: 1867–1876. doi:10.1097/QAD.0000000000001555
14. Assefa E, Figa Z. Acceptance of the Index Case HIV Testing and Associated Factors among Anti-Retroviral Therapy Patients in Public Health Facilities in Gedeo Zone, Southern Ethiopia. Cross-Sectional Study Design with Document Review. Inquiry (United States). 2024;61. doi:10.1177/00469580241271185
15. Gizaw YT, Boke MM, Geremew AB. Determinants of hiv testing uptake among partners of pregnant women in addis ababa, ethiopia: A community-based study. Pan African Medical Journal. 2021;39. doi:10.11604/pamj.2021.39.7.27839

Formatted: French (Caribbean)

16. Lindsay BR, Mwango L, Toeque MG, Malupande SL, Nkhuwa E, Moonga CN, et al. Peer community health workers improve HIV testing and ART linkage among key populations in Zambia: retrospective observational results from the Z-CHECK project, 2019–2020. J Int AIDS Soc. 2022;25. doi:10.1002/jia2.26030
17. Tarekegn D, Habtegiorgis SD, Telayneh AT, Mitiku KW, Adugna A, Alemayehu D, et al. The Magnitude and Associated Factors of Early Index Case Testing Among Adult HIV Index Cases at Debre Markos Town High Load Health Facilities 2023. AIDS Res Treat. 2025: 8237131. doi:10.1155/arat/8237131
18. Onovo A, Kalaiwo A, Agweye A, Emmanuel G, Keiser O. Diagnosis and case finding according to key partner risk populations of people living with HIV in Nigeria: A retrospective analysis of community-led index partner testing services. EClinicalMedicine. 2022;43. doi:10.1016/j.eclinm.2021.101265
19. Munthali T, Masano A, Chen JS, Kumwenda W, Pedersen C, Chinthenga K, et al. Assisted partner notification as a strategy to enhance Pre-Exposure Prophylaxis (PrEP) screening and uptake – results from a prospective cohort study in Lilongwe, Malawi. BMC Health Serv Res. 2025;25. doi:10.1186/s12913-025-13038-4
20. Khandu L, Hallett J, Crawford G, Leavy JE, Vujcich D. HIV self-testing and partner notification strategies for key populations in low- to upper-middle-income countries: A mixed-methods systematic review. PLoS One. 2025;20. doi:10.1371/journal.pone.0338639
21. Brown LB, Miller WC, Kamanga G, Nyirenda N, Mmodzi P, Pettifor A, et al. HIV partner notification is effective and feasible in sub-Saharan Africa: Opportunities for HIV treatment and prevention. J Acquir Immune Defic Syndr (1988). 2011;56: 437–442. doi:10.1097/QAI.0b013e318202bf7d
22. Mugisha N, Tirera F, Coulibaly-Kouyate N, Aguie W, He Y, Kemper K, et al. Implementation process and challenges of index testing in Côte d'Ivoire from healthcare workers' perspectives. PLoS One. 2023;18: e0280623. doi:10.1371/journal.pone.0280623
23. Mshweshwe-Pakela N, Mabuto T, Lebyane R, Hamisi M, Gininza S, Chihota V, et al. HIV index testing implementation at public health clinics in South Africa. S Afr Med J. 2025;115. doi:10.7196/SAMJ.2025.v115i8.2652
24. Meek CJ, Munkhondya TEM, Mphande M, Tembo TA, Chitani M, Jean-Baptiste M, et al. Examining the feasibility of assisted index case testing for HIV case-finding: a qualitative analysis of barriers and facilitators to implementation in Malawi. BMC Health Services Research 2024 24:1. 2024;24: 606-. doi:10.1186/s12913-024-10988-z
25. Henley C, Forgwei G, Welty T, Golden M, Adimora A, Shields R, et al. Scale-up and case-finding effectiveness of an HIV partner services program in Cameroon: An innovative HIV prevention intervention for developing countries. Sex Transm Dis. 2013;40: 909–914. doi:10.1097/OLQ.0000000000000032
26. Perera S, Swartz A, Githaiga JN. "Getting involved": understanding informal practices, challenges and alternatives in assisted partner notification services for HIV in Cape Town, South Africa. AIDS Care. 2026 [cited 26 Feb 2026]. doi:10.1080/09540121.2026.2621031
27. Camara S, Millimouno TM, Kourouma M, Sow A, Sidibé S, Touré AB, et al. Facilitators and barriers to community-based HIV testing in Guinea: a CFIR-based implementation analysis. Front Public Health. 2025;13: 1593697. doi:10.3389/fpubh.2025.1593697
28. Bärnighausen K, Berner-Rodoreda A, McGowan M, Reñosa MD, Mtaita C, Neuhann F. 'You Can Get That Person on ART but You Can't Give Them Back Their Social System': A Qualitative Analysis of Voluntary Assisted Partner Notification for HIV for

Marginalised and Vulnerable Populations. J Int Assoc Provid AIDS Care. 2024;23. doi:10.1177/23259582241272059

29. Lewis ZH, Ottenbacher KJ, Fisher SR, Jennings K, Brown AF, Swartz MC, et al. The feasibility and RE-AIM evaluation of the TAME health pilot study. Int J Behav Nutr Phys Act. 2017;14. doi:10.1186/S12966-017-0560-5
30. Proctor E, Silmere H, Raghavan R, Hovmand P, Aarons G, Bunger A, et al. Outcomes for implementation research: conceptual distinctions, measurement challenges, and research agenda. Adm Policy Ment Health. 2011;38: 65–76. doi:10.1007/s10488-010-0319-7
31. UNAIDS. UNAIDS data 2022 | UNAIDS. In: report AIDS 2022 [Internet]. 2023 [cited 15 Sep 2025]. Available: https://www.unaids.org/en/resources/documents/2023/2022_unaids_data
32. The Global Funds. Office of the Inspector General . Guinea: Grant Audit Report. Geneva. 2024 Dec. Available: https://www.theglobalfund.org/media/15325/oig_gf-oig-24-019_report_fr.pdf?utm_source=chatgpt.com
33. Mugisha N, Tirera F, Coulibaly-Kouyate N, Aguie W, He Y, Kemper K, et al. Implementation process and challenges of index testing in Côte d'Ivoire from healthcare workers' perspectives. PLoS One. 2023;18: e0280623. doi:10.1371/journal.pone.0280623
34. Meek CJ, Munkhondya TEM, Mphande M, Tembo TA, Chitani M, Jean-Baptiste M, et al. Examining the feasibility of assisted index case testing for HIV case-finding: a qualitative analysis of barriers and facilitators to implementation in Malawi. Res Sq. 2023; rs.3.rs-3314925. doi:10.21203/rs.3.rs-3314925/v1
35. Craig P, Dieppe P, Macintyre S, Mitchie S, Nazareth I, Petticrew M. Developing and evaluating complex interventions: the new Medical Research Council guidance. The BMJ. 2008;337: a1655. doi:10.1136/bmj.a1655
36. Consolidated guidelines on HIV testing services (2015). [cited 7 Nov 2025]. Available: https://www.who.int/publications/i/item/9789241508926?
37. D'Lima D, Soukup T, Hull L. Evaluating the Application of the RE-AIM Planning and Evaluation Framework: An Updated Systematic Review and Exploration of Pragmatic Application. Front Public Health. 2022;9. doi:10.3389/FPUBH.2021.755738/PDF
38. Burnham KP, Anderson DR. Multimodel inference: Understanding AIC and BIC in model selection. Sociol Methods Res. 2004;33: 261–304. doi:10.1177/0049124104268644
39. Brewer MJ, Butler A, Cooksley SL. The relative performance of AIC, AICC and BIC in the presence of unobserved heterogeneity. Methods Ecol Evol. 2016;7: 679–692. doi:10.1111/2041-210X.12541
40. Burnham KP, Anderson DR, Huyvaert KP. AIC model selection and multimodel inference in behavioral ecology: Some background, observations, and comparisons. Behav Ecol Sociobiol. 2011;65: 23–35. doi:10.1007/S00265-010-1029-6
41. Zamawe FC. The Implication of Using NVivo Software in Qualitative Data Analysis: Evidence-Based Reflections. Malawi Medical Journal. 2015;27: 13. doi:10.4314/MMJ.V27I1.4
42. Gale NK, Heath G, Cameron E, Rashid S, Redwood S. Using the framework method for the analysis of qualitative data in multi-disciplinary health research. BMC Med Res Methodol. 2013;13. doi:10.1186/1471-2288-13-117
43. Hoff TJ, Amick HR, Lund JL, Lee SYD. Review: Use of Qualitative Methods in Published Health Services and Management Research: A 10-Year Review. Medical Care Research and Review. 2011;68: 3–33. doi:10.1177/1077558710372810

Formatted: French (Caribbean)

44. Hlongwa M, Basera W, Hlongwana K, Lombard C, Laubscher R, Duma S, et al. Linkage to HIV care and early retention in HIV care among men in the 'universal test-and-treat' era in a high HIV-burdened district, KwaZulu-Natal, South Africa. BMC Health Services Research 2024 24:1. 2024;24: 384-. doi:10.1186/S12913-024-10736-3
45. Hlongwa M, Hlongwana K, Makhunga S, Choko AT, Dzinamarira T, Conserve D, et al. Linkage to HIV Care Following HIV Self-testing Among Men: Systematic Review of Quantitative and Qualitative Studies from Six Countries in Sub-Saharan Africa. AIDS and Behavior 2022 27:2. 2022;27: 651–666. doi:10.1007/S10461-022-03800-8
46. Lippman SA, De Kadt J, Ratlhagana MJ, Agnew E, Gilmore H, Sumitani J, et al. Impact of short message service and peer navigation on linkage to care and antiretroviral therapy initiation in South Africa. AIDS. 2023;37: 647–657. doi:10.1097/QAD.0000000000003453
47. Tavakoli F, Karamouzian M, Haghdoost AA, Mirzazadeh A, Dehghan M, Bazrafshani MS, et al. Barriers and facilitators of HIV partner status notification in low- and lower-middle-income countries: A mixed-methods systematic review. BMC Infectious Diseases 2024 24:1. 2024;24: 1404-. doi:10.1186/S12879-024-10241-2
48. Sanena M, Hagstrom C, Phiri K, Kalande P, Songo J, Maruwo L, et al. Intimate partner violence among HIV index case testing clients in Malawi, a mixed-methods study. PLOS Global Public Health. 2025;5: e0003818. doi:10.1371/JOURNAL.PGPH.0003818
49. Traore IT, Meda N, Hema NM, Ouedraogo D, Some F, Some R, et al. HIV prevention and care services for female sex workers: Efficacy of a targeted community-based intervention in Burkina Faso. J Int AIDS Soc. 2015;18. doi:10.7448/IAS.18.1.20088
50. Kirakoya-Samadoulougou F, Jean K, Maheu-Giroux M. Uptake of HIV testing in Burkina Faso: an assessment of individual and community-level determinants. BMC Public Health. 2017;17. doi:10.1186/s12889-017-4417-2
51. Boisvert Moreau M, Kintin FD, Atchekpe S, Batona G, Béhanzin L, Guédou FA, et al. HIV self-testing implementation, distribution and use among female sex workers in Cotonou, Benin: a qualitative evaluation of acceptability and feasibility. BMC Public Health. 2022;22. doi:10.1186/S12889-022-12917-3
52. Oyebade AO, Bello MA, Familusi IO. Effect of the antenatal hiv testing scale up community intervention in south west, nigeria: A cross-sectional study. Pan African Medical Journal. 2021;40. doi:10.11604/pamj.2021.40.90.30230
53. Mohammed A, Cheabu BSN, Amoah-Larbi J, Osei FA, Benyah G, Asampong R, et al. Perception and experience of HIV-induced stigma among people with HIV seeking healthcare in Ghana. BMC Health Serv Res. 2024;24. doi:10.1186/S12913-024-11930-Z
54. Coulaud PJ, Préau M, Maradan G, Mora M, Traoré F, Oga M, et al. Taking empowerment into account: the response of community-based organisations to the HIV care needs of men who have sex with men in West Africa (CohMSM ANRS 12324 - Expertise France). AIDS Care. 2019;31: 1403–1411. doi:10.1080/09540121.2019.1587358
55. Arije O, Titus R, Olaniran A, Dadi A, Garba D, Okeke E, et al. Effectiveness of community mobilisation models in improving HIV testing services uptake among women and children in Nigeria: A quasi-experimental study. Glob Public Health. 2023;18. doi:10.1080/17441692.2023.2284880
56. Arije O, Titus R, Omisile I, Dadi A, Garba D, Godpower O, et al. Process evaluation of the 'Lafiyan Yara' project on enhancing access to HIV testing services using existing community structures in Nigeria. BMC Public Health. 2024;24. doi:10.1186/s12889-024-18045-4

Formatted: French (Caribbean)

Formatted: French (Caribbean)

57. Camara S, Millimouno TM, Kourouma M, Sow A, Sidibé S, Touré AB, et al. Facilitators and barriers to community-based HIV testing in Guinea: a CFIR-based implementation analysis. Front Public Health. 2025;13: 1593697. doi:10.3389/fpubh.2025.1593697
58. Perray M, Traore D, Riegel L, Rojas Castro D, Spire B, Mora M, et al. Benefits and challenges of a community-based programme for women living with HIV in Mali. AIDS Care. 2023;35: 1100–1106. doi:10.1080/09540121.2023.2208319
59. Abubakari GMR, Turner DA, Ni Z, Conserve DF, Dada D, Otchere A, et al. Community-Based Interventions as Opportunities to Increase HIV Self-Testing and Linkage to Care Among Men Who Have Sex With Men – Lessons From Ghana, West Africa. Front Public Health. 2021;9. doi:10.3389/fpubh.2021.660256
60. Ngcobo S, Scheepers S, Mbatha N, Grobler E, Rossouw T. Roles, Barriers, and Recommendations for Community Health Workers Providing Community-Based HIV Care in Sub-Saharan Africa: A Review. AIDS Patient Care STDS. 2022;36: 130–144. doi:10.1089/apc.2022.0020
61. Hlongwa M, Mashamba-Thompson T, Makhunga S, Hlongwana K. Barriers to HIV testing uptake among men in sub-Saharan Africa: a scoping review. Afr J AIDS Res. 2020;19: 13–23. doi:10.2989/16085906.2020.1725071
62. Chikwari CD, Dringus S, Ferrand RA. Barriers to, and emerging strategies for, HIV testing among adolescents in sub-Saharan Africa. Curr Opin HIV AIDS. 2018;13: 257–264. doi:10.1097/COH.0000000000000452
63. Donessouné FMG, Sossa OG, Kouanda S. Using CFIR framework for understanding barriers and facilitators to implementation of community tuberculosis program in Burkina Faso. Frontiers in Health Services. 2023;3: 1294176. doi:10.3389/FRHS.2023.1294176/BIBTEX
64. Conserve DF, Msofe J, Issango J, Tureski K, McCarthy P, Rwezahura P, et al. Development, Implementation, and Scale Up of the National Furaha Yangu Campaign to Promote HIV Test and Treat Services Uptake Among Men in Tanzania. Am J Mens Health. 2022;16. doi:10.1177/15579883221087838
65. Grabowski MK, Mpagazi J, Kiboneka S, Ssekubugu R, Kereba JB, Nakayijja A, et al. The HIV and sexually transmitted infection syndemic following mass scale-up of combination HIV interventions in two communities in southern Uganda: a population-based cross-sectional study. Lancet Glob Health. 2022;10: e1825–e1834. doi:10.1016/S2214-109X(22)00424-7
66. Oyebade AO, Bello MA, Familusi IO. Effect of the antenatal hiv testing scale up community intervention in south west, nigeria: A cross-sectional study. Pan African Medical Journal. 2021;40. doi:10.11604/pamj.2021.40.90.30230
67. Okere NE, Lennox L, Urlings L, Ford N, Naniche D, Rinke De Wit TF, et al. Exploring Sustainability in the Era of Differentiated HIV Service Delivery in Sub-Saharan Africa: A Systematic Review. J Acquir Immune Defic Syndr. 2021;87: 1055–1071. doi:10.1097/QAI.0000000000002688
68. Nwaozuru U, Murphy P, Richard A, Obiezu-Umeh C, Shato T, Obionu I, et al. The sustainability of health interventions implemented in Africa: an updated systematic review on evidence and future research perspectives. Implement Sci Commun. 2025;6. doi:10.1186/S43058-025-00716-X

Formatted: French (Caribbean)

Supporting information

S1 Fig (TIF): Geographic distribution of intervention sites for community-based HIV testing in Guinea (2021–2024). Pilot and scale-up districts implementing community index testing through community-based organizations (CBOs) linked to PMTCT sites**.** Map produced in QGIS v3.44.4 using administrative boundaries from GADM (https://gadm.org/; https://gadm.org/license.html).

S2 Fig: Program theory linking PMTCT sites and community-based organizations (CBOs) within the HIV index case testing cascade in Guinea (2021–2024). The figure illustrates the hypothesized pathways connecting community sensitization, testing, and link (TIF)

S3 Table. Operationalization of RE-AIM. (DOCX)
Definition of the RE-AIM dimensions and corresponding indicators, including calculation methods, data sources, and analytical approaches used to evaluate the community-based HIV index case testing program in Guinea (2021–2024).

S4 Text. Detailed statistical analysis. (DOCX)
Description of study variables, mixed-effects modelling approach, diagnostics, sensitivity analyses, and statistical software/packages used to assess HIV test acceptance, referral, and ART initiation outcomes (Guinea, 2021–2024).

S5 Table. NVivo codebook aligned with the RE-AIM framework. (DOCX)
Qualitative coding framework used for thematic analysis, including operational definitions of codes by RE-AIM dimension and representative illustrative quotes from key informants and beneficiaries.

S6 Table. Implementation phases of the community-based HIV testing program. (DOCX)
Summary of implementation phases (2021–2023), including intensity, operational objectives, key mechanisms, and expected quantitative signals used to interpret program performance over time.

S7 Fig. Mechanism of impact of community-based organizations in the index case HIV testing cascade (Guinea, 2021–2024). (TIF)
Conceptual diagram illustrating how CBO activities contribute to key cascade steps, from sensitization and contact elicitation to testing, referral, and linkage to care.

S8 Data. De-identified aggregated dataset and codebook (XLSX).
Anonymized aggregated quarterly program dataset used for quantitative analyses of community-based index case HIV testing in Guinea (2021–2024), including codebook and README.

Response to Reviewer Comments

Manuscript: Evaluation of community-based HIV index case testing using the RE-AIM framework in Guinea
Journal: *PLOS Global Public Health*

Dear Reviewer,

We sincerely thank the reviewer for the careful reading of our manuscript and for the constructive comments. We are grateful for the positive assessment of the manuscript and have revised the paper to address each point raised. The main revisions include updating Figure 2 with subgroup sample sizes, expanding the Discussion and Conclusion regarding the markedly lower linkage to care among men, clarifying the ethical treatment of minors represented in routine programme data, and harmonizing table formatting.

Comment 1: Fig 2 needs sample size for each population so that it can stand alone.

Response:
Thank you for this helpful suggestion. We agree that Figure 2 should include the sample size for each subgroup so that it can be interpreted independently of the main text.

While revising the figure, we re-examined the specification of the mixed-effects model and clarified that the outcome displayed in Figure 2 was linkage to care among HIV-positive individuals, rather than HIV test acceptance among referred contacts. The numerical estimates and fitted model remain unchanged; however, the outcome label and interpretation have been corrected throughout the manuscript.

The revised Figure 2 now reports the analytic denominators for each subgroup: women, n = 389; children aged <15 years, n = 238; and men, n = 109. The figure legend has also been revised to clarify that the values represent adjusted marginal predicted probabilities with 95% confidence intervals, derived from a mixed-effects binomial logistic regression model with a CBO-level random intercept.

Comment 2: The poor acceptance rate among men was not addressed adequately in the discussion or conclusions as it is a critical issue impacting on

the female partner's retention on treatment as well as issues of GBV. Use of peers is a recognised strategy which could be addressed as one of the possible interventions to improve acceptance, linkage and retention.

Response:

Thank you for highlighting this important issue. We agree that the markedly lower probability observed among men required fuller discussion and clearer programmatic interpretation.

As noted above, the outcome shown in Figure 2 has now been clarified as linkage to care among HIV-positive individuals. We have therefore revised the Discussion and Conclusion to describe this finding as a major male engagement gap in the post-diagnosis care pathway. The revised Discussion now considers plausible explanations, including stigma, confidentiality concerns, difficulties with intra-household disclosure, gender-related norms, limited engagement with health services, and service-level barriers.

We have also added a cautious discussion of the potential implications for partner support, voluntary disclosure, women's retention in care, and gender-based or intimate-partner violence. We explicitly state that the study did not measure women's retention according to male partner linkage status and did not assess gender-based violence outcomes; therefore, no causal relationship is inferred.

In response to the reviewer's suggestion, we have added male-responsive and peer-supported strategies as potential programmatic adaptations. These include trained male peer navigators, trusted community champions, confidential counselling, flexible service-delivery modalities, supported referral, and safety-centred disclosure approaches. We present these as strategies that should be prospectively evaluated, rather than as effects demonstrated by the present study.

Comment 3: Under ethical considerations, it is mentioned that no minors were included, however children <15 years were included in the study. There needs to be clarification that consent was taken from the parents/legal guardians due to the higher age of consent in the country. Was no assent requested? Please clarify.

Response:

Thank you for identifying this important ambiguity. We have revised the Study Population, Data Sources and Collection, and Ethical Considerations sections to clearly distinguish between children represented in routine programme records and participants recruited specifically for research.

Children aged <15 years were included only through de-identified routine programme records used for secondary quantitative analysis. They were beneficiaries of standard HIV programme services and were not contacted, recruited, or interviewed by the research team. No research-specific procedures were conducted with minors.

All participants recruited for qualitative interviews were adults aged 18 years or older. Therefore, parental or legal guardian permission and child assent were not applicable to the interview component.

The study protocol, including the secondary analysis of de-identified routine programme data, was approved by the National Ethics Committee for Health Research of Guinea (CNERS, Ref. No. 060/CNERS/23). No additional research-specific parental permission or child assent was sought for the secondary analysis of these de-identified routine records, as this analysis was covered by the CNERS-approved protocol. Consent procedures related to routine HIV testing and care were governed separately by national programme procedures and were distinct from consent for participation in this research.

Comment 4: Minor formatting issues in the tables, all text in the cell needs left justification, as it is not consistent.

Response:

Thank you for noting this formatting inconsistency. We have revised all tables to ensure consistent left alignment of cell contents and harmonized the formatting of headings, numeric entries, footnotes, and spacing throughout the manuscript.

Closing statement:

We appreciate the reviewer's constructive comments, which helped improve the clarity, ethical transparency, and programmatic interpretation of the manuscript. We believe that the revisions have strengthened the paper and hope that the revised version satisfactorily addresses all remaining concerns.

Sincerely,

The Authors

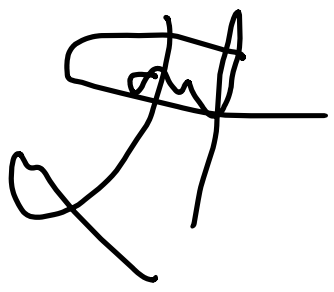

Supplement: Supplementary file 3 [file pgph.0005461.s013.pdf]
