# Supplementary material for: RE-AIM evaluation of the national scale-up of community-based index case HIV testing in Guinea: Successes and challenges
Source: PLOS Glob Public Health. 2026 Nov 10;6(11):e0005461. doi: 10.1371/journal.pgph.0005461 (PMC13750574; doi:10.1371/journal.pgph.0005461)

Response to Reviewer Comments

Manuscript: Evaluation of community-based HIV index case testing using the RE-AIM framework in Guinea
Journal: *PLOS Global Public Health*

Dear Reviewer,

We sincerely thank the reviewer for the careful reading of our manuscript and for the constructive comments. We are grateful for the positive assessment of the manuscript and have revised the paper to address each point raised. The main revisions include updating Figure 2 with subgroup sample sizes, expanding the Discussion and Conclusion regarding the markedly lower linkage to care among men, clarifying the ethical treatment of minors represented in routine programme data, and harmonizing table formatting.

Comment 1: Fig 2 needs sample size for each population so that it can stand alone.

Response:
Thank you for this helpful suggestion. We agree that Figure 2 should include the sample size for each subgroup so that it can be interpreted independently of the main text.

While revising the figure, we re-examined the specification of the mixed-effects model and clarified that the outcome displayed in Figure 2 was linkage to care among HIV-positive individuals, rather than HIV test acceptance among referred contacts. The numerical estimates and fitted model remain unchanged; however, the outcome label and interpretation have been corrected throughout the manuscript.

The revised Figure 2 now reports the analytic denominators for each subgroup: women, n = 389; children aged <15 years, n = 238; and men, n = 109. The figure legend has also been revised to clarify that the values represent adjusted marginal predicted probabilities with 95% confidence intervals, derived from a mixed-effects binomial logistic regression model with a CBO-level random intercept.

Comment 2: The poor acceptance rate among men was not addressed adequately in the discussion or conclusions as it is a critical issue impacting on

the female partner's retention on treatment as well as issues of GBV. Use of peers is a recognised strategy which could be addressed as one of the possible interventions to improve acceptance, linkage and retention.

Response:

Thank you for highlighting this important issue. We agree that the markedly lower probability observed among men required fuller discussion and clearer programmatic interpretation.

As noted above, the outcome shown in Figure 2 has now been clarified as linkage to care among HIV-positive individuals. We have therefore revised the Discussion and Conclusion to describe this finding as a major male engagement gap in the post-diagnosis care pathway. The revised Discussion now considers plausible explanations, including stigma, confidentiality concerns, difficulties with intra-household disclosure, gender-related norms, limited engagement with health services, and service-level barriers.

We have also added a cautious discussion of the potential implications for partner support, voluntary disclosure, women’s retention in care, and gender-based or intimate-partner violence. We explicitly state that the study did not measure women’s retention according to male partner linkage status and did not assess gender-based violence outcomes; therefore, no causal relationship is inferred.

In response to the reviewer’s suggestion, we have added male-responsive and peer-supported strategies as potential programmatic adaptations. These include trained male peer navigators, trusted community champions, confidential counselling, flexible service-delivery modalities, supported referral, and safety-centred disclosure approaches. We present these as strategies that should be prospectively evaluated, rather than as effects demonstrated by the present study.

Comment 3: Under ethical considerations, it is mentioned that no minors were included, however children <15 years were included in the study. There needs to be clarification that consent was taken from the parents/legal guardians due to the higher age of consent in the country. Was no assent requested? Please clarify.

Response:

Thank you for identifying this important ambiguity. We have revised the Study Population, Data Sources and Collection, and Ethical Considerations sections to clearly distinguish between children represented in routine programme records and participants recruited specifically for research.

Children aged <15 years were included only through de-identified routine programme records used for secondary quantitative analysis. They were beneficiaries of standard HIV programme services and were not contacted, recruited, or interviewed by the research team. No research-specific procedures were conducted with minors.

All participants recruited for qualitative interviews were adults aged 18 years or older. Therefore, parental or legal guardian permission and child assent were not applicable to the interview component.

The study protocol, including the secondary analysis of de-identified routine programme data, was approved by the National Ethics Committee for Health Research of Guinea (CNERS, Ref. No. 060/CNERS/23). No additional research-specific parental permission or child assent was sought for the secondary analysis of these de-identified routine records, as this analysis was covered by the CNERS-approved protocol. Consent procedures related to routine HIV testing and care were governed separately by national programme procedures and were distinct from consent for participation in this research.

Comment 4: Minor formatting issues in the tables, all text in the cell needs left justification, as it is not consistent.

Response:

Thank you for noting this formatting inconsistency. We have revised all tables to ensure consistent left alignment of cell contents and harmonized the formatting of headings, numeric entries, footnotes, and spacing throughout the manuscript.

Closing statement:

We appreciate the reviewer's constructive comments, which helped improve the clarity, ethical transparency, and programmatic interpretation of the manuscript. We believe that the revisions have strengthened the paper and hope that the revised version satisfactorily addresses all remaining concerns.

Sincerely,

The Authors

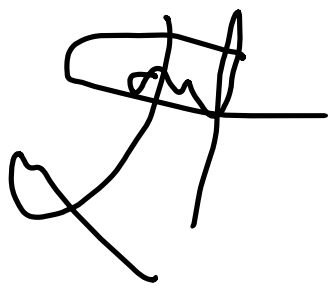

Supplement: Supplementary file 4 [file pgph.0005461.s015.pdf]
