# Supplementary material for: RE-AIM evaluation of the national scale-up of community-based index case HIV testing in Guinea: Successes and challenges
Source: PLOS Glob Public Health. 2026 Nov 10;6(11):e0005461. doi: 10.1371/journal.pgph.0005461 (PMC13750574; doi:10.1371/journal.pgph.0005461)

Response to Reviewer Comments

Manuscript ID: PGPH-D-25-03261R3

"RE-AIM evaluation of the national scale-up of community-based index case HIV testing in Guinea: Successes and Challenges"

Journal: *PLOS Global Public Health*

Dear Editor and Reviewer,

We sincerely thank the reviewer for the careful reading of our revised manuscript and for the constructive comments. We have revised the manuscript to address each point raised, with particular attention to clarifying the RE-AIM operationalization and data analysis, strengthening the interpretation of implementation findings, clarifying the distinction between intervention phases, and making the overall conclusion more explicit. Reviewer comments are shown in bold, followed by our responses.

Responses to line-specific comments

Comment 1. Line 25-26: Switch the parenthesis to be around the acronym so it's "Reach, Effectiveness, Adoption, Implementation, and Maintenance (RE-AIM)".

Response: Thank you for this suggestion. We revised the Abstract so that the full name of the framework appears before the acronym: "Reach, Effectiveness, Adoption, Implementation, and Maintenance (RE-AIM)." We also removed the repeated definition later in the Abstract to improve clarity and avoid redundancy.

Comment 2. Line 87: Delete "widely applied in public health".

Response: Thank you for this suggestion. We deleted the phrase "widely applied in public health" to make the sentence more concise and direct.

Comment 3. Lines 78-92: Delete the sentence "Evaluating complex interventions requires frameworks…occurs." Then start the paragraph with the last two sentences in the paragraph.

Response: Thank you for this suggestion. We deleted the sentence beginning "Evaluating complex interventions requires frameworks…" and revised the paragraph to begin directly with

the definition and purpose of the RE-AIM framework. This change improves concision and avoids redundancy in the Background section.

Comment 4. Line 101: Instead of "based on" use "using".

Response: Thank you. We revised the wording as suggested and replaced "based on" with "using."

Comment 5. Line 106: Make "Quantitative were analyzed … May 19, 2025" its own sentence. Then make "Qualitative interviews and document reviews … January 5, 2024" its own sentence.

Response: Thank you. We revised this section so that the timing of the quantitative analysis and the timing of the qualitative interviews and document review are presented in two separate sentences.

Comment 6. Line 122: Delete the information in the parenthesis, RE-AIM was already defined earlier.

Response: Thank you. We removed the parenthetical definition of RE-AIM at this point to avoid repeating an acronym that had already been defined earlier in the manuscript.

Comment 7. Line 243: Start a new paragraph for "Adoption".

Response: Thank you. We inserted a new paragraph beginning with the Adoption dimension to improve readability and make the operationalization of the RE-AIM dimensions easier to follow.

Comment 8. Line 270: Please re-write as "For the subgroup analysis of HIV-positive individuals (Fig 2), a separate grouped binomial mixed-effects logistic regression model was fitted to estimate linkage to care."

Response: Thank you for this suggestion. We revised the sentence to clarify that the subgroup analysis was conducted among HIV-positive individuals and that a separate grouped binomial mixed-effects logistic regression model was fitted to estimate linkage to care. Because a schematic RE-AIM framework figure has now been added earlier in the manuscript, the subgroup linkage-to-care figure is now referenced as Fig 3 in the revised version.

Comment 9. Line 272: Remove "specified as".

Response: Thank you. We removed "specified as" and simplified the sentence to improve clarity.

Comment 10. Line 352-356: The first two sentences should be in the data analysis section.

Response: Thank you for this suggestion. We moved the two sentences from the Effectiveness results section to the Data Analysis section because they describe analytical indicators rather than results. The Effectiveness section now begins directly with the observed findings across implementation phases.

Comment 11. Line 370-372: Besides the split of this analysis per phase, it would be important to also look into data for districts separated, to understand if there was lower performance through time on sites already implementing the strategy or if the reduction in effectiveness is due to new sites beginning the implementation.

Response: Thank you for this important suggestion. We conducted an additional descriptive analysis stratified by district implementation maturity, comparing pilot districts with scale-up districts during 2023-2024. After harmonizing the testing-referral denominator with the main manuscript definition, the weighted testing-referral rates were low in both pilot and scale-up districts in 2023 (7.2% and 5.4%, respectively) and increased sharply in both groups in 2024 (99.6% and 96.7%, respectively). This pattern suggests that the 2023 decline was not solely explained by the inclusion of newly implementing districts. We also clarified that test uptake estimates for 2023 were not interpreted because denominator inconsistencies produced values exceeding 100%. The corresponding findings have been added to the Results section and detailed in S9 Table. The S9 Table note also clarifies that ART initiation was used as an operational proxy for post-diagnosis linkage to care in this supplementary analysis because the routine aggregated dataset did not contain a separate linkage-to-care variable.

Comment 12. Line 382-385: Needs to go in the data analysis section.

Response: Thank you. We moved this text to the Data Analysis section, where it now helps explain how Adoption and Maintenance were used to contextualize the Effectiveness findings.

Comment 13. Line 457-459 & 465-467: These are the same sentences, one should be removed.

Response: Thank you for identifying this duplication. We removed the repeated sentence and retained a single consolidated statement to avoid redundancy.

Comment 14. Line 507-508: The beginning sentence of this paragraph doesn't really match the rest of the paragraph.

Response: Thank you for this comment. We agree that the opening sentence of this paragraph was not well aligned with the main focus of the paragraph, which discusses the lower post-diagnosis linkage to care among men. We revised the beginning of the paragraph to introduce the sex-related gap in linkage to care more directly and removed the less specific opening statement.

Comment 15. Lines 502-503: Only linkage to treatment was presented disaggregated; can we present for the entire cascade above?

Response: Thank you for this helpful comment. We revised the opening paragraphs of the Discussion to better summarize the full cascade of findings, including reach, organizational adoption, testing referral, test uptake, linkage to care, and ART initiation. We also clarified that the age- and sex-related subgroup finding applies specifically to linkage to care among individuals diagnosed HIV-positive, rather than to the entire cascade. The revised text now frames the Discussion around three principal findings: uneven effectiveness across cascade steps and implementation phases, the three-phase implementation trajectory, and fragile institutional sustainability.

Comment 16. Line 534: New paragraph with "In our study…".

Response: Thank you. We started a new paragraph at "In our study…" to improve the structure and readability of the Discussion.

Comment 17. Line 547: State what the implementation pattern is, pattern in what? Refer to the result that shows this pattern.

Response: Thank you for this helpful comment. We revised the paragraph to specify that the implementation pattern refers to phase-level changes in cascade performance across the three implementation stages documented in the Results. We now explicitly refer to the results shown in Table 1 and S9 Table, including fluctuations in testing referral and test uptake during scale-

up. In light of the additional district-stratified analysis, we also nuanced the interpretation of the 2023 decline. The revised text no longer attributes this decline primarily to a dilution effect from newly added districts; instead, it presents it as more consistent with broader programme-level instability during scale-up, while acknowledging that routine programme data do not allow the relative contribution of expansion-related effects and other operational disruptions to be quantified.

Responses to thematic comments

Background

Reviewer comment: If possible, please include a figure or visual of the RE-AIM framework.

Response: Thank you for this suggestion. We agree that a visual representation of the RE-AIM framework improves clarity. We added a simple schematic figure illustrating the five RE-AIM dimensions and how they guided the present evaluation, including indicator selection and interpretation of programme performance, implementation processes, and sustainability.

Description of intervention

Reviewer comment: What was the further expansion from Phase 2 to Phase 3? What was the substantial increase from 17 districts to how many from Phase 2 to Phase 3? How is Phase 2 different from Phase 3?

Response: Thank you for this important clarification. We agree that the previous wording could be interpreted as suggesting an additional expansion beyond 17 districts during Phase 3. We revised the Description of the Intervention to clarify the distinction between the phases. Phase 1 was the pilot phase, implemented by five CBOs across three districts. Phase 2 was the operational adjustment and roll-out phase, during which the intervention was extended beyond the pilot districts and organized around the planned scale-up platform of 26 CBOs across 17 districts. Phase 3 was not a further geographic expansion beyond 17 districts; rather, it corresponded to routine implementation and consolidation at full scale during 2023-2024. We also corrected the wording to make clear that the substantial increase refers to the transition from the initial pilot to the full-scale implementation platform.

Data Analysis

Reviewer comment: The data analysis paragraph is a little confusing. It might be clearer to put the "Operationalization of the RE-AIM Framework" under the Data Analysis section, then you can put what methods were used for each component of the RE-AIM framework in the section. How were outcome variables measured? How many models were run in total? What were the explanatory variables of interest? How were they defined? Which components utilized the qualitative findings?

Response: Thank you for this helpful comment. We agree that the previous Data Analysis section was difficult to follow because the operationalization of the RE-AIM dimensions, outcome definitions, units of analysis, model specifications, and qualitative integration were presented across separate or dense paragraphs. We therefore reorganized the section under the revised heading "Operationalization of RE-AIM indicators and data analysis." The revised section now explicitly links each RE-AIM dimension to its indicators and analytical approach: Reach is analysed descriptively using sensitization, counselling, referral, and testing counts; Effectiveness is assessed through testing referral, test uptake, linkage to care, and ART initiation outcomes; Adoption is assessed through the number of districts and CBOs implementing the intervention; Implementation is assessed through implementation intensity, interannual stability, cascade correlations, and qualitative findings on operational delivery; and Maintenance is assessed through CBO continuity, reporting completeness, supply-chain disruption signals, and institutional and financial sustainability. We also clarified how the outcome variables were measured, including testing referral among counselled individuals, test uptake among referred individuals, and post-diagnosis linkage/ART initiation among individuals diagnosed HIV-positive. In addition, the revised section specifies that six model-based quantitative analyses were conducted, identifies the explanatory variables of interest (sex, age group, calendar year, implementation intensity, and age-sex category for the subgroup model), defines these variables, and clarifies that qualitative findings were used primarily for the Implementation and Maintenance dimensions while also contextualizing observed patterns in Reach, Effectiveness, and Adoption where relevant.

Discussion

Reviewer comment: The discussion section is a little confusing and may benefit from dividing the third paragraph into smaller more focused paragraphs. Discuss what implementation findings (Tables 2 & 3) mean. Is this done in lines 534-546? If so, make

it more explicit that is what is being discussed. The HIV-positive women were identified via PMTCT services. Is it possible that low male participation could be due to recruiting through this service in particular, as the program may be seen as healthcare primarily for women and children and men do not see themselves as part of it? Is it possible social norms around women and men's health and HIV in Guinea play a role in low male engagement?

Response: Thank you for these helpful comments. We revised the Discussion to improve clarity and divided the previously long paragraph into shorter, more focused paragraphs. We now separately discuss male engagement, the interpretation of implementation findings, and phase-level changes in cascade performance. We also made the interpretation of Tables 2 and 3 more explicit. The revised text explains that Table 2 shows substantial interannual variability in cascade indicators, while Table 3 shows strong operational coherence between upstream activities such as sensitization, counselling, and testing, but a more vulnerable transition from community testing to post-diagnosis care. We compare this interpretation with findings from Burkina Faso and other West African settings, where irregular commodity availability, stigma-related barriers, and weak referral coordination have limited the conversion of community-based testing into effective linkage to care. In response to the comment on male engagement, we expanded the Discussion to consider the PMTCT entry point and gender norms. We now note that, because HIV-positive women were identified through PMTCT services, male partners may have perceived the intervention pathway as primarily oriented toward women and children. We also discuss broader social norms around masculinity, health-seeking behaviour, and HIV-related stigma as plausible contributors to low male engagement. These explanations are presented cautiously because the evaluation did not collect male-specific qualitative data on reasons for non-linkage.

Conclusion

Reviewer comment: Do the findings show good, bad, or mixed performance?

Response: Thank you for this helpful comment. We revised the Conclusion to state more clearly that the findings show mixed programme performance. The revised conclusion now acknowledges the programme's strengths, including broad reach, wide organizational adoption, and generally high linkage to care and ART initiation among individuals diagnosed HIV-positive, particularly among women and children. It also highlights the main weaknesses, including fluctuating referral and test uptake during scale-up, markedly lower linkage among

men, and persistent sustainability constraints related to commodity supply, data systems, external financing, and institutional integration. We believe this revised wording provides a more balanced and explicit interpretation of the overall findings.

We thank the reviewer again for these helpful comments, which have strengthened the clarity, methodological transparency, and interpretation of the manuscript.

Sincerely,

The Authors

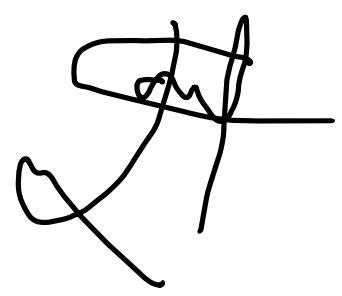

Supplement: Supplementary file 5 [file pgph.0005461.s016.pdf]
