# Supplementary material for: RE-AIM evaluation of the national scale-up of community-based index case HIV testing in Guinea: Successes and challenges
Source: PLOS Glob Public Health. 2026 Nov 10;6(11):e0005461. doi: 10.1371/journal.pgph.0005461 (PMC13750574; doi:10.1371/journal.pgph.0005461)

Response to the Editor and Reviewers

Manuscript ID: PGPH-D-25-03261R4

Title: *RE-AIM evaluation of the national scale-up of community-based index case HIV testing in Guinea: Successes and Challenges*

Journal: *PLOS Global Public Health*

Dear Editor and Reviewers,

We sincerely thank the Editor and the reviewers for their careful assessment of our manuscript and for their constructive comments. We appreciate the positive evaluation of the study's methodological rigor, statistical analyses, presentation, and contribution to the evidence on community-based index case HIV testing.

We have addressed the specific comments raised during this review round. In addition, while preparing the revised submission, we incorporated a limited number of methodological and consistency clarifications identified through an additional internal review. These author-initiated revisions improve the transparency, accuracy, and reproducibility of the manuscript without changing its principal results, overall interpretation, or conclusions.

All changes are shown in the marked-up manuscript. A clean version of the revised manuscript is also provided.

Response to the Editor

Comment:
"I request the authors to define '95-95-95' at its first mention, either in the main text or in a footnote, to ensure clarity for readers who may be unfamiliar with the term prior to publication."

Response:
We thank you for this important suggestion. We have now defined the UNAIDS 95-95-95 targets at their first occurrence in the Abstract and reiterated the definition in the Introduction to ensure that both sections can be understood independently.

The revised text reads:

"The UNAIDS 95-95-95 targets call for 95% of people living with HIV to know their HIV status, 95% of those diagnosed with HIV to receive sustained antiretroviral therapy, and 95% of those receiving antiretroviral therapy to achieve viral suppression."

Response to Reviewer 5

Comment:
"Line 64: Please provide the full definition of the 95-95-95 UNAIDS targets in a footnote. Not every reader may be familiar with them."

Response:
We thank the reviewer for highlighting the need to clarify this terminology. As recommended, the three components of the UNAIDS 95-95-95 targets have now been defined at their first mention. For greater visibility and clarity, we incorporated the definition directly into the text rather than placing it in a footnote. The definition has been included in both the Abstract and the Introduction.

Additional author-initiated revisions

During preparation of this revision, we conducted an additional internal methodological and consistency review. This review resulted in the following targeted clarifications and corrections:

1. **Clarification of the mixed-methods design**

We clarified the chronology and integration of the quantitative and qualitative components. Because the qualitative interviews preceded the final quantitative analyses, the study is no longer characterized as a formal explanatory sequential design. The manuscript now describes the study more accurately as a mixed-methods implementation evaluation in which the two components were integrated at the interpretation stage.

2. **Clarification of the RE-AIM operationalization**

We strengthened the operational definitions of Reach, Effectiveness, Adoption, Implementation, and Maintenance. We also explicitly acknowledged the measurement limitations of each dimension, including the inability to assess population representativeness, staff-level adoption, comprehensive implementation fidelity, and long-term sustainability beyond the observation period.

3. **Harmonization of cascade definitions and denominators**

We reviewed and harmonized the definitions and denominators used for testing referral, test uptake, HIV positivity, linkage to care, and antiretroviral therapy initiation. We also clarified the calculations based on the 2024 programme data to ensure consistency throughout the Methods, Results, tables, and figures.

4. **Correction of the description of programme expansion**

We replaced the previously reported percentage increases in programme coverage with more transparent and verifiable fold-change estimates. The revised manuscript now reports that the intervention expanded from 3 to 17 districts and from 5 to 26 implementing community-based organizations between 2021 and 2023, corresponding to 5.7-fold and 5.2-fold increases, respectively.

5. **More cautious interpretation of descriptive statistical analyses**

We revised the presentation of the coefficients of variation and Spearman rank correlations to emphasize their exploratory and descriptive nature. We removed universal interpretive thresholds and avoided causal or mechanistic interpretations, given the aggregated structure of the data, the limited number of region-year observations, repeated measurements within regions, and the absence of adjustment for multiple comparisons.

6. **Addition of data-quality and cleaning procedures**

We added a subsection describing the procedures used to assess data completeness, plausibility, and internal consistency. The manuscript now explains how indicator labels, reporting periods, and organizational identifiers were harmonized and how non-interpretable observations with impossible cascade relationships were identified and handled before analysis.

7. **Correction of bibliographic inconsistencies**

We corrected minor inconsistencies in the reference list, including a duplicate reference, an inaccurate publication year, and an unnecessary tracking parameter contained in a URL. These corrections do not affect the interpretation of the cited evidence.

Overall impact of the additional revisions

These additional revisions improve the manuscript's methodological clarity, internal consistency, transparency, and reproducibility. They do not alter the study objectives, principal findings, overall interpretation, or conclusions.

We hope that the revised manuscript satisfactorily addresses the Editor's and reviewer's comment. We sincerely appreciate the time and expertise devoted to evaluating our work.

Kind regards,

Dr Soriba Camara
Corresponding Author
On behalf of all authors

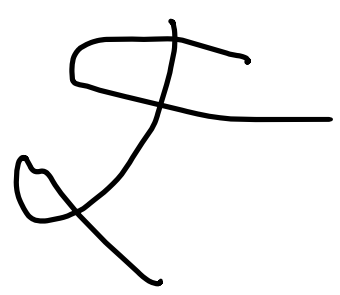

Supplement: Supplementary file 6 [file pgph.0005461.s017.pdf]
