# Supplementary material for: RE-AIM evaluation of the national scale-up of community-based index case HIV testing in Guinea: Successes and challenges
Source: PLOS Glob Public Health. 2026 Nov 10;6(11):e0005461. doi: 10.1371/journal.pgph.0005461 (PMC13750574; doi:10.1371/journal.pgph.0005461)
Supplement: Supplementary file 7 [file pgph.0005461.s003.docx]

S1 Table: Operationalization of RE-AIM indicators and corresponding analytical approaches. (DOCX)

| **RE-AIM dimension** | **Indicator** | **Definition / calculation** | **Data source** | **Analytical approach** |
| --- | --- | --- | --- | --- |
| Reach | Individuals sensitized | Total number of individuals exposed to community awareness or sensitization activities in covered areas | Routine programme monitoring data; consolidated reports | Descriptive totals and proportions by implementation phase |
| Reach | Individuals counselled | Number of individuals receiving post-identification counselling after risk identification | Routine programme monitoring data; KoboCollect | Descriptive totals and proportions by implementation phase |
| Reach | Individuals referred for HIV testing | Number of sensitized or counselled individuals referred for HIV testing, according to routine programme records | Routine programme monitoring data; KoboCollect | Descriptive cascade analysis; testing-referral rate |
| Reach | Individuals tested | Number of individuals who completed HIV testing through the community-based strategy | Routine programme monitoring data; KoboCollect | Descriptive cascade analysis; annual and phase-specific trends |
| Effectiveness | Testing-referral rate | Proportion of sensitized individuals referred for HIV testing | Routine programme monitoring data; KoboCollect | Descriptive estimates by implementation phase; district-maturity analysis in S9 Table |
| Effectiveness | Test uptake | Proportion of referred individuals who completed HIV testing | Routine programme monitoring data; KoboCollect | Descriptive estimates; mixed-effects binomial regression where applicable; non-interpretable estimates excluded when denominators produced values exceeding 100% |
| Effectiveness | Linkage to care | Proportion of individuals diagnosed HIV-positive who were linked to care | Routine programme monitoring data; PMTCT/ART records | Mixed-effects binomial regression; subgroup analysis by age-sex category; estimated marginal means and 95% confidence intervals |
| Effectiveness | ART initiation | Proportion of individuals diagnosed HIV-positive who initiated ART | Routine programme monitoring data; PMTCT/ART records | Descriptive estimates; mixed-effects binomial regression where applicable |
| Adoption | Districts covered | Number of districts implementing the intervention over time | Programme reports; PNLSH records | Descriptive temporal analysis of geographic adoption |
| Adoption | CBOs engaged | Number of CBOs implementing the intervention over time | Programme reports; CBO registry; GFATM reports | Descriptive temporal analysis of organizational adoption |
| Implementation | Implementation intensity | Median number of testing sessions conducted per CBO per reporting period | Routine programme monitoring data; KoboCollect | Descriptive analysis of central tendency and variability |
| Implementation | Interannual stability | Coefficient of variation of key cascade indicators across years | Routine programme monitoring data, 2021-2024 | Coefficient of variation; interannual stability analysis |
| Implementation | Cascade correlations | Associations between sensitization, counselling, testing, HIV positivity, and linkage to care | Aggregated region × year programme data | Spearman rank correlation coefficients and correlation matrix |
| Implementation | Operational delivery factors | Perceived facilitators and barriers affecting delivery, including supervision, counsellor turnover, commodity supply, stigma, and referral coordination | Qualitative interviews | Thematic analysis guided by RE-AIM; quant-qual triangulation |
| Maintenance | CBO continuity | Continuity of CBO engagement during routine implementation | Programme reports; CBO registry | Descriptive assessment of organizational continuity |
| Maintenance | Reporting completeness | Proportion of critical indicators reported in the consolidated routine database | Consolidated programme database; KoboCollect | Completeness assessment by indicator |
| Maintenance | Supply-chain and commodity disruption signals | Evidence of interruptions or irregularity in HIV testing commodities and referral pathways | Qualitative interviews; routine programme records where available | Quantitative–qualitative triangulation |
| Maintenance | Institutional and financial sustainability | Evidence of continued dependence on external financing and degree of integration into national programme systems | Programme documents; PNLSH/GFATM reports; qualitative interviews | Descriptive document review and thematic analysis |

Note: RE-AIM = Reach, Effectiveness, Adoption, Implementation, and Maintenance; CBO = community-based organization; PMTCT = prevention of mother-to-child transmission; ART = antiretroviral therapy. Age-sex disaggregation was conducted for linkage to care among individuals diagnosed HIV-positive and was not applied to all cascade steps.
