# Supplementary material for: RE-AIM evaluation of the national scale-up of community-based index case HIV testing in Guinea: Successes and challenges
Source: PLOS Glob Public Health. 2026 Nov 10;6(11):e0005461. doi: 10.1371/journal.pgph.0005461 (PMC13750574; doi:10.1371/journal.pgph.0005461)
Supplement: Supplementary file 8 [file pgph.0005461.s004.docx]

**S1 Text: Detailed Statistical Analysis. (DOCX)**

**Overview**

The statistical analysis was designed to assess the performance of the community-based HIV testing program and to identify factors associated with (i) HIV test acceptance, (ii) referral to care, and (iii) initiation of antiretroviral therapy (ART). The analytical framework combined descriptive, multilevel, and temporal approaches to capture both individual- and organizational-level dynamics.

**Study Variables**

- **Primary outcomes:**
  1. **Testing referral**: proportion of counselled individuals referred for HIV testing.
  2. **Test uptake**: proportion of referred individuals who completed HIV testing.
  3. **Linkage to care among HIV-positive individuals**: assessed in the subgroup analysis using grouped binomial mixed-effects logistic regression.
  4. **ART initiation**: proportion of individuals diagnosed HIV-positive who initiated ART.
- **Covariates:**
  Sex (male/female), age group (<16 years vs. ≥16 years), year of implementation, intervention intensity (number of community testing sessions per period), and the proportion of individuals sensitized by subgroup. These covariates were selected based on programmatic relevance and prior evidence from sub-Saharan HIV testing interventions.

**Modeling Approach**

Mixed-effects binomial logistic regression models were applied to account for the hierarchical structure of the data (individuals nested within community-based organizations, CBOs). Fixed effects represented individual- and programme-level covariates, while random intercepts captured inter-CBO heterogeneity. In the general model, the outcome probability was modelled as a function of individual- and programme-level covariates, with a random intercept for CBO to account for clustering of observations within implementing organizations.

$$logit(P(Y\mathrm{ij}=1))=\beta0+\beta X\mathrm{ij}+uj+\varepsilon\mathrm{ij}$$

$Yij$ is the probability of HIV test acceptance for individual $i$ within CBO $j$ , $Xi$the vector of covariates, and $uj$ the random effect associated with CBO.

**Diagnostics and Model Fit**

- **Overdispersion:** assessed by comparing residual deviance to model degrees of freedom.
- **Collinearity:** evaluated using **Variance Inflation Factors (VIF)**, with a threshold of <5 to exclude problematic multicollinearity.
- **Goodness of fit:** evaluated using **Akaike Information Criterion (AIC)** and **Bayesian Information Criterion (BIC)**. The retained model (AIC = 13,214.6; BIC = 13,294.7) showed satisfactory parsimony.
- **Random effects:** inter-CBO variance (σ² = 1.214; SD = 1.102) indicated measurable heterogeneity between implementing organizations.

**Complementary Analyses**

- **Indicator stability:** measured using **coefficients of variation (CV)** to identify the most stable (treatment initiation) and most volatile (testing session intensity) components of implementation.
- **Inter-dimensional relationships:** explored through **Spearman’s rank correlations**, suitable for nonparametric distributions, to assess associations between outreach, counseling, testing, positivity, and linkage indicators.
- **District-maturity supplementary analysis:** To examine whether changes in cascade performance over time reflected declining performance in districts already implementing the strategy or lower performance among newly implementing districts, an additional descriptive analysis was conducted for 2023-2024. Districts were classified as pilot districts if they participated in the 2021 pilot phase and as scale-up districts if they entered during subsequent programme expansion. Weighted aggregate rates were calculated by pooling numerators and denominators within each implementation-maturity group and year. Testing-referral rate was calculated as the total number of individuals referred for HIV testing divided by the total number of counselled individuals. Test uptake was calculated as the total number tested divided by the total number referred. In this supplementary analysis, ART initiation was used as an operational proxy for post-diagnosis linkage to care because the routine aggregated dataset did not contain a separate linkage-to-care variable.
- **Temporal trends:** assessed using mixed-effects longitudinal models with repeated annual observations at the CBO level, complemented by descriptive analyses across implementation phases and district-maturity groups.

**Sensitivity and Robustness Checks**

Interaction terms were explored for significant covariates and retained if they improved model fit (ΔAIC < 2). Diagnostic plots confirmed the adequacy of the logistic link and the normality of random effects. Analyses were reproduced with and without missing data imputation to confirm the robustness of results.

**Software and Packages**

All analyses were performed in **R version 4.3.1** (R Core Team, 2024). The following packages were used:

- **lme4** (mixed-effects models)
- **car** (collinearity diagnostics via VIF)
- **ggplot2** (data visualization)
- **emmeans** (marginal probability estimation)
- **psych** and **Hmisc** (Spearman correlations and descriptive statistics)

**Marginal predicted probabilities**

Marginal predicted probabilities were derived from the fitted mixed-effects logistic regression models using the *emmeans* package. Predicted probabilities were calculated for each subgroup and then averaged over the observed distribution of covariates, yielding marginal (population-averaged) estimates. These values therefore correspond to model-based adjusted probabilities rather than crude observed proportions. Ninety-five percent confidence intervals were obtained from model-based standard errors using the delta method.

**Longitudinal models**

Longitudinal trends in referral and linkage indicators were analysed using mixed-effects linear regression models with repeated measures at the CBO level. Time was parameterised as a continuous calendar-year variable (2021–2024), which allowed estimation of the average annual change in outcomes. Each CBO contributed repeated annual observations, and a random intercept for CBO was included to account for within-Organisation correlation over time. Fixed effects included calendar year and intervention intensity, with sensitivity analyses additionally adjusting for district setting (urban vs rural) when available. Robust standard errors were applied to account for potential heteroskedasticity. The estimated annual change of −2.54 percentage points per year reported in the main text corresponds to the coefficient of the time variable in these longitudinal models.

**Reproducibility and Data Management**

Quantitative analyses were conducted on complete-case observations (*n* = 2,255) for regression models, while the total sensitized population (*n* = 50,609) was used for descriptive statistics.
