# Supplementary material for: RE-AIM evaluation of the national scale-up of community-based index case HIV testing in Guinea: Successes and challenges
Source: PLOS Glob Public Health. 2026 Nov 10;6(11):e0005461. doi: 10.1371/journal.pgph.0005461 (PMC13750574; doi:10.1371/journal.pgph.0005461)
Supplement: Supplementary file 9 [file pgph.0005461.s005.docx]

**S2 Table: NVivo 14 Codebook - RE-AIM Framework. (DOCX**)

| **Dimension** | **Code** | **Definition** | **Representative Quote** |
| --- | --- | --- | --- |
| **Reach** | Target population | Characteristics and extent of individuals effectively reached by the intervention | “90% of our clients tested are women.” (CBO 6) |
|  | Mobilization channels | Means and networks used to inform and recruit (local leaders, community relays) | “We always go through the sector chief, he gives us authorization to conduct our activities.” (CBO 7) |
|  | Access barriers | Obstacles to participation (taboos, stigma, transportation costs) | “Many categorically refuse; they fear lack of confidentiality and community judgment.” (CBO 3) |
| **Effectiveness** | Impact on serological status | Measurable or perceived changes in testing and treatment initiation rates | “Since I came to this center, no child has been born HIV-positive from an HIV-positive mother.” (HC 4) |
|  | Reduction in transmission | Effect on the prevention of new infections | “Thanks to this approach, we have reduced the number of infections in the community.” (CBO 6) |
|  | Perceived acceptability | Degree of satisfaction and trust among beneficiaries | “I find this method very simple and reassuring; it guarantees confidentiality.” (CAS 4) |
| **Adoption** | Institutional engagement | Level of commitment and partnership of health centers and NGOs | “Our organization is supported by two health centers; there is good collaboration.” (CBO 8) |
|  | Leader involvement | Role of local authorities and elected officials in supporting the strategy | “Local officials are informed and agree with the idea of community testing.” (CBO 9) |
|  | Counselor motivation | Training, material, and financial support for community counselors | “Our allowances need to arrive on time; otherwise, our counselors are not motivated.” (CBO 7) |
| **Implementation** | Protocol adaptability | Degree of adjustment of the testing process to local contexts | “We do mass testing next to families, then test two neighboring houses.” (CBO 5) |
|  | Confidentiality safeguards | Measures taken to ensure discretion and trust | “We test in an isolated office so that no one can hear the result.” (CBO 4) |
|  | Commodity availability | Management of resources (kits, ARVs, motorcycles, facilities) | “We often face stockouts of tests is needed to continue.” (CBO 12) |
| **Maintenance** | Financial sustainability | Sources and stability of funding in the long term | “As long as commodities are available, we can continue.” (CBO 4) |
|  | Institutional integration | Degree to which the activity is incorporated into national budgets and protocols | “These activities must gradually be integrated into the public budget.” (Coordinator 1) |
|  | Activity continuity | Ability to sustain services and awareness over time | “If I have the will, resources, and commodities, I can continue this project to save lives.” (CBO 3) |

- **HC** = Health Center staff
- **CAS** = Beneficiary/client participant
- **CBO** = Community-Based Organization.
