# Supplementary material for: RE-AIM evaluation of the national scale-up of community-based index case HIV testing in Guinea: Successes and challenges
Source: PLOS Glob Public Health. 2026 Nov 10;6(11):e0005461. doi: 10.1371/journal.pgph.0005461 (PMC13750574; doi:10.1371/journal.pgph.0005461)
Supplement: Supplementary file 10 [file pgph.0005461.s006.docx]

**S3 Table: Implementation phases of community-based HIV testing. (DOCX)**

| **Phase** | **Period** | **Intensity (median sessions/quarter)** | **Operational objective** | **Key mechanisms (examples)** | **Expected signals in data** |
| --- | --- | --- | --- | --- | --- |
| **Phase 1: Initial intensity** | 2021 | 15 | Rapidly launch community-based testing and achieve a critical volume | Mobilization of CBOs, rapid micro-planning, proximity sessions, prioritized commodity supply | Concurrent rise in outreach → counseling → tested; high variability at program start |
| **Phase 2: Adjustment** | 2022 | 1 | Expand geographically, stabilize routines, optimize procedures and targeting | Standardization of scripts, lighter scheduling, quality feedback, risk-based targeting | Lower but more “efficient” volumes; sustained number of positives despite reduced activity |
| **Phase 3: Consolidation** | 2023 | 3 | Consolidate achievements are full scale | Routine implementation/areas, logistical reinforcement, strengthened referral partnerships | Recovery of upstream volumes; sustained/increased linkage-to-care proportional to positives |
