# Supplementary material for: RE-AIM evaluation of the national scale-up of community-based index case HIV testing in Guinea: Successes and challenges
Source: PLOS Glob Public Health. 2026 Nov 10;6(11):e0005461. doi: 10.1371/journal.pgph.0005461 (PMC13750574; doi:10.1371/journal.pgph.0005461)
Supplement: Supplementary file 11 [file pgph.0005461.s009.docx]

**S4 Table: Cascade performance by district implementation maturity, Guinea, 2023-2024. (DOCX)**

| **District implementation maturity** | **Year** | **Number of districts** | **Total sensitized** | **Total counselled** | **Total referred** | **Total tested** | **Total HIV-positive** | **Total linked to care / ART initiated proxy** | **Testing-referral rate (%)** | **Test uptake rate (%)** | **Linkage-to-care proxy rate (%)** |
| --- | --- | --- | --- | --- | --- | --- | --- | --- | --- | --- | --- |
| Pilot districts | 2023 | 3 | 7,407 | 4,063 | 294 | 2,233 | 106 | 105 | 7.2 | Not interpreted | 99.1 |
| Pilot districts | 2024 | 3 | 6,859 | 4,108 | 4,092 | 2,181 | 156 | 145 | 99.6 | 53.3 | 92.9 |
| Scale-up districts | 2023 | 14 | 8,400 | 4,620 | 249 | 2,499 | 160 | 132 | 5.4 | Not interpreted | 82.5 |
| Scale-up districts | 2024 | 14 | 7,773 | 4,818 | 4,660 | 2,418 | 207 | 181 | 96.7 | 51.9 | 87.4 |

Note: Districts were classified as pilot districts if they participated in the 2021 pilot phase and as scale-up districts if they entered during subsequent programme expansion. Rates shown are weighted aggregate rates, calculated by pooling numerators and denominators within each implementation-maturity group and year, rather than by averaging district-level percentages. Testing-referral rate was calculated as the total number of individuals referred for HIV testing divided by the total number of counselled individuals. Test uptake was calculated as the total number tested divided by the total number referred. In this supplementary district-maturity analysis, ART initiation was used as an operational proxy for post-diagnosis linkage to care because the routine aggregated dataset did not contain a separate linkage-to-care variable. Test uptake estimates for 2023 were not interpreted because denominator inconsistencies produced values exceeding 100%.
