# Supplementary material for: RE-AIM evaluation of the national scale-up of community-based index case HIV testing in Guinea: Successes and challenges
Source: PLOS Glob Public Health. 2026 Nov 10;6(11):e0005461. doi: 10.1371/journal.pgph.0005461 (PMC13750574; doi:10.1371/journal.pgph.0005461)
Supplement: Supplementary file 12 [file pgph.0005461.s014.docx]

Very interesting paper, health system integration of HIV programs is vital and identifying strengths and weaknesses is important. The RE-AIM framework was helpful in guiding the evaluation. With further clarification of methods and discussion the findings from this paper are very useful for HIV program development and scale-up. There are a lot of moving parts so a clear concise summary of findings in the conclusion would make the paper stronger.

Line 25-26: Switch the parenthesis to be around the acronym so it’s “Reach, Effectiveness, Adoption, Implementation, and Maintenance (RE-AIM)

Line 87: Delete “widely applied in public health”

Lines 78-92: Delete the following sentence “Evaluating complex interventions requires frameworks…occurs.” Then start the paragraph with the last two sentences in the paragraph. “The RE-AIM framework (Reach, Effectiveness, Adoption, Implementation, Maintenance) offers a structured approach to assess population coverage, behavioral……..and sustainability. This multidimensional lens…..and institutional anchoring.”

Line 101: Instead of “based on” use “using”

Line 106: Make “Quantitative were analyzed ….May 19, 2025.” its own sentence. Then make “Qualitative interviews and document reviews…..January 5, 2024.” its own sentence.

Line 122: Delete the information in the parenthesis, RE-AIM was already defined earlier

Line 243: Start a new paragraph for “Adoption”

Line 254: Start a new paragraph for “Maintenance”

Line 270: Please re-write as “For the subgroup analysis of HIV-positive individuals (Fig 2), a separate grouped binomial mixed-effects logistic regression model was fitted to estimate linkage to care.”

Line 272: Remove “specified as”

Line 352-356: The first two sentences should be in the data analysis section.

Line 382-385: Needs to go in the data analysis section

Line 457-459 & 465-467: These are the same sentences, one should be removed.

Line 507-508: The beginning sentence of this paragraph doesn’t really match the rest of the paragraph.

Line 534: New paragraph with “In our study…”

Line 547: State what the implementation pattern is, pattern in what? Refer to the result that shows this pattern.

**Background**

If possible, please include a figure or visual of the RE-AIM framework

**Description of intervention**

What was the further expansion from Phase 2 to Phase 3? What was the substantial increase from 17 districts to how many from Phase 2 to Phase 3? How is Phase 2 different from Phase 3?

**Data Analysis**

The data analysis paragraph is a little confusing. It might be clearer to put the “Operationalization of the RE-AIM Framework” under the Data Analysis section, then you can put what methods were used for each component of the RE-AIM framework in the section. How were outcome variables measured? How many models were run in total? What were the explanatory variables of interest? How were they defined? Which components utilized the qualitative findings?

**Discussion**

The discussion section is a little confusing and may benefit from dividing the third paragraph into smaller more focused paragraphs.

Discuss what implementation findings (tables 2 & 3) mean. Is this done in lines 534-546? If so, make it more explicit that is what is being discussed.

The HIV-positive women were identified via PMTCT services. Is it possible that low male participation could be due to recruiting through this service in particular, as the program may be seen as healthcare primarily for women and children and men do no see themselves as part of it?

Is it possible social norms around women and men’s health and HIV in Guinea play a role in low male engagement?

**Conclusion**

Do the findings show good, bad, or mixed performance?
