# Supplementary material for: RE-AIM evaluation of the national scale-up of community-based index case HIV testing in Guinea: Successes and challenges
Source: PLOS Glob Public Health. 2026 Nov 10;6(11):e0005461. doi: 10.1371/journal.pgph.0005461 (PMC13750574; doi:10.1371/journal.pgph.0005461)
Supplement: Peer review history

# Peer review: Decision Letter 0

**DOI:** 10.1371/journal.pgph.0005461.r001

**Authors:** Helen Howard

12 Dec 2025

PGPH-D-25-03261

RE-AIM evaluation of the national scale-up of community-based index case HIV testing in Guinea: Successes and Challenges

PLOS Global Public Health

Dear Dr. CAMARA,

Thank you for submitting your manuscript to PLOS Global Public Health. After careful consideration, we feel that it has merit but does not fully meet PLOS Global Public Health’s publication criteria as it currently stands. Therefore, we invite you to submit a revised version of the manuscript that addresses the points raised during the review process.

Please note that we have only been able to secure a single reviewer to assess your manuscript. We are issuing a decision on your manuscript at this point to prevent further delays in the evaluation of your manuscript. Please be aware that the editor who handles your revised manuscript might find it necessary to invite additional reviewers to assess this work once the revised manuscript is submitted. However, we will aim to proceed on the basis of this single review if possible.

Could you please carefully revise the manuscript to address all comments raised?

Please submit your revised manuscript by Jan 25 2026 11:59PM. If you will need more time than this to complete your revisions, please reply to this message or contact the journal office at globalpubhealth@plos.org. When you're ready to submit your revision, log on to https://www.editorialmanager.com/pgph/ and select the 'Submissions Needing Revision' folder to locate your manuscript file.

Please include the following items when submitting your revised manuscript:

- A rebuttal letter that responds to each point raised by the editor and reviewer(s). You should upload this letter as a separate file labeled 'Response to Reviewers'.
- A marked-up copy of your manuscript that highlights changes made to the original version. You should upload this as a separate file labeled 'Revised Manuscript with Track Changes'.
- An unmarked version of your revised paper without tracked changes. You should upload this as a separate file labeled 'Manuscript'.

Guidelines for resubmitting your figure files are available below the reviewer comments at the end of this letter.

We look forward to receiving your revised manuscript.

Kind regards,

Helen Howard

Staff Editor

PLOS Global Public Health

Journal Requirements:

1.  Please amend your detailed Financial Disclosure statement. This is published with the article. It must therefore be completed in full sentences and contain the exact wording you wish to be published.

1. Please clarify all sources of funding (financial or material support) for your study. List the grants (with grant number) or organizations (with url) that supported your study, including funding received from your institution.

2. State the initials, alongside each funding source, of each author to receive each grant.

3. State what role the funders took in the study. If the funders had no role in your study, please state: “The funders had no role in study design, data collection and analysis, decision to publish, or preparation of the manuscript.”

4. If any authors received a salary from any of your funders, please state which authors and which funders.

If you did not receive any funding for this study, please simply state: “The authors received no specific funding for this work.”

2. Please send a completed 'Competing Interests' statement, including any COIs declared by your co-authors. If you have no competing interests to declare, please state "The authors have declared that no competing interests exist".

3. In the online submission form, you indicated that The study's findings are corroborated by data obtained from national HIV program records and community-based implementing partners in Guinea. De-identified aggregated datasets used in the analyses can be requested from the corresponding author.

All PLOS journals now require all data underlying the findings described in their manuscript to be freely available to other researchers, either

1. In a public repository,

2. Within the manuscript itself, or

3. Uploaded as supplementary information.

This policy applies to all data except where public deposition would breach compliance with the protocol approved by your research ethics board. If your data cannot be made publicly available for ethical or legal reasons (e.g., public availability would compromise patient privacy), please explain your reasons by return email and your exemption request will be escalated to the editor for approval. Your exemption request will be handled independently and will not hold up the peer review process, but will need to be resolved should your manuscript be accepted for publication. One of the Editorial team will then be in touch if there are any issues.

4. We notice that your supplementary [figures/tables] are included in the manuscript file. Please remove them and upload them with the file type 'Supporting Information'. Please ensure that each Supporting Information file has a legend listed in the manuscript after the references list.

5. Some material included in your submission may be copyrighted. According to PLOS’s copyright policy, authors who use figures or other material (e.g., graphics, clipart, maps) from another author or copyright holder must demonstrate or obtain permission to publish this material under the Creative Commons Attribution 4.0 International (CC BY 4.0) License used by PLOS journals. Please closely review the details of PLOS’s copyright requirements here: PLOS Licenses and Copyright. If you need to request permissions from a copyright holder, you may use PLOS's Copyright Content Permission form.

Please respond directly to this email or email the journal office and provide any known details concerning your material's license terms and permissions required for reuse, even if you have not yet obtained copyright permissions or are unsure of your material's copyright compatibility.

Potential Copyright Issues:

1. S1 Fi: please (a) provide a direct link to the base layer of the map (i.e., the country or region border shape) and ensure this is also included in the figure legend; and (b) provide a link to the terms of use / license information for the base layer image or shapefile. We cannot publish proprietary or copyrighted maps (e.g. Google Maps, Mapquest) and the terms of use for your map base layer must be compatible with our CC-BY 4.0 license.

Note: if you created the map in a software program like R or ArcGIS, please locate and indicate the source of the basemap shapefile onto which data has been plotted.

If your map was obtained from a copyrighted source please amend the figure so that the base map used is from an openly available source. Alternatively, please provide explicit written permission from the copyright holder granting you the right to publish the material under our CC-BY 4.0 license.

Please note that the following CC BY licenses are compatible with PLOS license: CC BY 4.0, CC BY 2.0 and CC BY 3.0, meanwhile such licenses as CC BY-ND 3.0 and others are not compatible due to additional restrictions.

If you are unsure whether you can use a map or not, please do reach out and we will be able to help you. The following websites are good examples of where you can source open access or public domain maps:

* U.S. Geological Survey (USGS) - All maps are in the public domain. (http://www.usgs.gov)

* PlaniGlobe - All maps are published under a Creative Commons license so please cite “PlaniGlobe, http://www.planiglobe.com, CC BY 2.0” in the image credit after the caption. (http://www.planiglobe.com/?lang=enl)

* Natural Earth - All maps are public domain. (http://www.naturalearthdata.com/about/terms-of-use/)

6. If the reviewer comments include a recommendation to cite specific previously published works, please review and evaluate these publications to determine whether they are relevant and should be cited. There is no requirement to cite these works unless the editor has indicated otherwise.

[Note: HTML markup is below. Please do not edit.]

Reviewers' comments:

Reviewer's Responses to Questions

**Comments to the Author**

1. Does this manuscript meet PLOS Global Public Health’s publication criteria?

Reviewer #1: Yes

**********

2. Has the statistical analysis been performed appropriately and rigorously?

Reviewer #1: Yes

**********

3. Have the authors made all data underlying the findings in their manuscript fully available (please refer to the Data Availability Statement at the start of the manuscript PDF file)?

The PLOS Data policy

Reviewer #1: No

**********

4. Is the manuscript presented in an intelligible fashion and written in standard English?

Reviewer #1: Yes

**********

Reviewer #1: Thank you for the opportunity to review this interesting manuscript.. The paper addresses an important topic and is generally clearly written. However, several areas require clarification and additional detail to strengthen the rigor, transparency, and interpretability of the findings.

1. Clarification of Independent Data Collection (Line 105)

The statement that “Data collection and analysis were conducted independently by researchers who were not part of the implementation teams” is unclear. Does this imply that none of the current manuscript’s co-authors were involved in implementation activities? Please explicitly identify who constituted the implementation teams and clarify the relationship between implementers and data collectors and analysis team.

2. Selection of Participating Districts (Line 112)

Can the authors describe the rationale of choosing the 17 participating districts? Were they chosen based on program presence, burden of disease, convenience, or other criteria?

3. Data Sources and Reporting Procedures

The distinction between routine programmatic data and data collected via the KoboCollect mobile application requires further explanation. What specific information was captured in KoboCollect versus routine reporting systems? Who was responsible for completing each dataset (e.g., CBO staff, project investigators, community health workers)? Please clarify.

4. Roles of Study Participants (Line 130)

The manuscript notes involvement of 28 participants, but their specific roles and contributions remain vague. Please detail the nature of their involvement (e.g., CBO leadership, field implementers, counselors, district-level officials).

5. Geographic Scope (Line 141)

The text mentions “three districts” without naming them. Please specify which districts these analyses pertain to.

6. Description of Interventions and Support Provided by CBOs (Line 151)

The intervention description lacks sufficient detail. Please elaborate on:

- Types of psychosocial support offered

- Frequency and mechanism of appointment reminders

- Criteria triggering reminders

- Nature and frequency of follow-up visits, and whether visits were home-based, clinic-based, and targeted to specific subgroups

This information is essential for understanding implementation fidelity and replicability.

7. Testing Acceptance in 2023 (Value of 1,2224.4%)

The testing acceptance value reported for 2023 is anomalous and not interpretable. This outlier substantially distorts summary calculations. I strongly recommend removing or correcting this value and describing the underlying issue in the manuscript.

8. Low Referral Rate in 2023

The manuscript should address the substantial decline in the referral rate in 2023. What operational or contextual factors contributed to this drop?

9. Figure 2 – Definition of Marginal Predicted Probabilities

The term “marginal predicted probabilities” needs clarification. Are these estimates derived from statistical models, or do they reflect empirically observed proportions? Please specify the modeling approach and how predictions were obtained.

10. Description of Longitudinal Models (Lines 261–264)

The statistical model used for the longitudinal analyses is not adequately described. Please detail the model specifications, including:

- Type of model (e.g., mixed-effects regression, GEE)

- Time parameterization

- Covariates included

-Handling of repeated measures

11. Indicators of Adoption and Figure 3C

The indicators used to assess adoption across CBOs need clearer definition. Figure 3C, in its current form, provides little added value and may be more suitable as descriptive text or a table. In addition, consider reporting the proportion of eligible sites that adopted the intervention. Were any eligible CBOs or districts invited but chose not to participate?

Minor Comments

- Line 102: “Doctoral research” is unnecessary. Simply refer to “the protocol” or “the study.”

- Line 119: Please list or describe the local languages used in data collection.

**********

what does this mean?). If published, this will include your full peer review and any attached files.

**Do you want your identity to be public for this peer review?**  If you choose “no”, your identity will remain anonymous but your review may still be made public.

For information about this choice, including consent withdrawal, please see our Privacy Policy

Reviewer #1: No

**********

[NOTE: If reviewer comments were submitted as an attachment file, they will be attached to this email and accessible via the submission site. Please log into your account, locate the manuscript record, and check for the action link "View Attachments". If this link does not appear, there are no attachment files.]

# Author comment: Author response to Decision Letter 1

**DOI:** 10.1371/journal.pgph.0005461.r002

20 Jan 2026

Attachment Submitted filename: Response_to_Reviewers.pdf

# Peer review: Decision Letter 1

**DOI:** 10.1371/journal.pgph.0005461.r003

**Authors:** Andrew Kazibwe

3 Feb 2026

PGPH-D-25-03261R1

RE-AIM evaluation of the national scale-up of community-based index case HIV testing in Guinea: Successes and Challenges

PLOS Global Public Health

Dear Dr. CAMARA,

Thank you for submitting your manuscript to PLOS Global Public Health. After careful consideration, we feel that it has merit but does not fully meet PLOS Global Public Health’s publication criteria as it currently stands. Therefore, we invite you to submit a revised version of the manuscript that addresses the points raised during the review process.

==============================

Thank you for addressing the comments in the first round of review. Kindly address all the comments in this round for a more technically sound article.

==============================

Please submit your revised manuscript by Mar 20 2026 11:59PM. If you will need more time than this to complete your revisions, please reply to this message or contact the journal office at globalpubhealth@plos.org. When you're ready to submit your revision, log on to https://www.editorialmanager.com/pgph/ and select the 'Submissions Needing Revision' folder to locate your manuscript file.

Please include the following items when submitting your revised manuscript:

- A letter that responds to each point raised by the editor and reviewer(s). You should upload this letter as a separate file labeled 'Response to Reviewers'.
- A marked-up copy of your manuscript that highlights changes made to the original version. You should upload this as a separate file labeled 'Revised Manuscript with Track Changes'.
- An unmarked version of your revised paper without tracked changes. You should upload this as a separate file labeled 'Manuscript'.

Guidelines for resubmitting your figure files are available below the reviewer comments at the end of this letter.

We look forward to receiving your revised manuscript.

Kind regards,

Andrew Kazibwe, MBChB, MMED

Academic Editor

PLOS Global Public Health

Journal Requirements:

If the reviewer comments include a recommendation to cite specific previously published works, please review and evaluate these publications to determine whether they are relevant and should be cited. There is no requirement to cite these works unless the editor has indicated otherwise.

Please review your reference list to ensure that it is complete and correct. If you have cited papers that have been retracted, please include the rationale for doing so in the manuscript text, or remove these references and replace them with relevant current references. Any changes to the reference list should be mentioned in the rebuttal letter that accompanies your revised manuscript. If you need to cite a retracted article, indicate the article’s retracted status in the References list and also include a citation and full reference for the retraction notice.

Additional Editor Comments (if provided):

Lines 42 -43: There is no result to support the conclusion that institutionalizing CBO engagement will be required for ensuring long-term sustainability

Lines 44-46: The statement, “These findings provide critical evidence to guide differentiated testing strategies in francophone West Africa and similar low-resource settings” should be replaced by an actual recommendation for practice, policy or research, drawn from the evaluation findings. For example, “Programs aiming to expand community HIV testing coverage should……….”

Line 63: Rather than stating the individuals “targeted” in index case testing, I suggest that the authors describe what index case testing involves.

Line 66: Punctuate appropriately

Lines 68-71: Why is evidence on large-scale implementation and long-term integration of ICT in national systems, necessary? Have there been any studies that have documented challenges in ICT implementation?

Lines 72-73: What do these percentages translate to, in absolute numbers of individuals? How far is the 36% from global targets?

Lines 79-80: What is the rationale for describing ICT as a complex intervention?

Lines 85-87: Provide a reference.

Lines 91-93: How similar is the HIV burden in Guinea to the rest of francophone West Africa?

Lines 97 – 98: A cross-sectional design is inappropriate for quantitative end-term evaluation. Please revise as appropriate. To me, it appears the authors used serial cross-sectional studies to measure annual increase in number of active CBOs; a time-series design to compare inter-annual stability and funding trends, a before-and-after design to compare the number of CBOs remaining active at the end of the study period

Lines 152-153: Please revise the sentence, “Phase 3….,” as appropriate.

Line 170: Wouldn’t geographic coverage be a measure for “reach”? Did the program monitor additional numbers of health facilities implementing the ICT approach?

Lines 187-190: What informed the choice of Spearman’s correlation as the appropriate statistical tool to assess for relationships between activity volumes and cascade outcomes? What type of data were activity volumes? What type of data were the cascade outcomes? What was the unit of analysis?

Lines 191-195: Since the themes of the evaluation were predetermined from an existing theoretical framework, it may not be appropriate to describe the qualitative analysis as “inductive” in nature. Please provide additional information. Otherwise, it appears a deductive approach was used.

Line 206 – 208: To whom did informed consent apply? Most likely, only to respondents of the qualitative interviews. Did the 50,609 individuals reached through community awareness and mobilization activities also provide informed consent? This should be clarified.

Line 230: How did the authors determine that the performance in the launch phase was “strong”? This language is more appropriate where there was comparison with program targets.

The wording of effectiveness measures described in the results section lines 230 - 246, differs from those the authors intended to measure as described in lines 164 – 168. In the results section, the authors compare referral and testing acceptance

Table 3 – In the methods section, please provide more detail on how the findings presented in this table were generated.

Lines 309 – 315: Why was qualitative triangulation only done for the maintenance pillar of the RE-AIM evaluation?

Line 323: Is it true that the impact was coherent? In the adoption results, the authors reported a decline in referral and linkage to care at -2.54% per year (lines 261-263)

How were the themes in table 4 developed?

Lines 382 – 388: Which result supports the assertion that “the framework revealed that rapid adoption and geographic expansion by community-based organisations substantially increased activity volumes….,”? What was the annual change in total numbers of individuals reached? What was the counterfactual? Since the study did not have a comparison group of districts, this assertion may be erroneous.

Lines 397-398: Is it true that the use of the RE-AIM framework is uncommonly used in West African implementation research?

Lines 399 – 401: Which result supports the recommendation for institutionalization of community-based index testing? At what level of the healthcare system is institutionalization missing/sub-optimal?

Lines 415-417: To what extent did this model expand diagnostic coverage and reinforce continuity of care? What were the baseline values before implementation of the model?

S3 table: The authors intended to measure median number of testing sessions per period per CBO. Was this measured?

The authors should ensure that all the supplementary materials are appropriately referenced in the body of the manuscript.

[Note: HTML markup is below. Please do not edit.]

Reviewers' comments:

Reviewer's Responses to Questions

**Comments to the Author**

Reviewer #1: All comments have been addressed

**********

publication criteria?

Reviewer #1: Yes

**********

3. Has the statistical analysis been performed appropriately and rigorously?

Reviewer #1: Yes

**********

4. Have the authors made all data underlying the findings in their manuscript fully available (please refer to the Data Availability Statement at the start of the manuscript PDF file)?

The PLOS Data policy

Reviewer #1: No

**********

5. Is the manuscript presented in an intelligible fashion and written in standard English?

Reviewer #1: Yes

**********

Reviewer #1: Thank you for the opportunity to review the revised manuscript. All my comments have been well addressed.

**********

what does this mean?). If published, this will include your full peer review and any attached files.

**Do you want your identity to be public for this peer review?**  If you choose “no”, your identity will remain anonymous but your review may still be made public.

For information about this choice, including consent withdrawal, please see our Privacy Policy

Reviewer #1: No

**********

[NOTE: If reviewer comments were submitted as an attachment file, they will be attached to this email and accessible via the submission site. Please log into your account, locate the manuscript record, and check for the action link "View Attachments". If this link does not appear, there are no attachment files.]

# Author comment: Author response to Decision Letter 2

**DOI:** 10.1371/journal.pgph.0005461.r004

16 Mar 2026

Attachment Submitted filename: Response to Reviewers2.pdf

# Peer review: Decision Letter 2

**DOI:** 10.1371/journal.pgph.0005461.r005

**Authors:** Katherine Kokkinias

10 Jun 2026

PGPH-D-25-03261R2

RE-AIM evaluation of the national scale-up of community-based index case HIV testing in Guinea: Successes and Challenges

PLOS Global Public Health

Dear Dr. CAMARA,

Thank you for submitting your manuscript to PLOS Global Public Health. After careful consideration, we feel that it has merit but does not fully meet PLOS Global Public Health’s publication criteria as it currently stands. Therefore, we invite you to submit a revised version of the manuscript that addresses the points raised during the review process.

==============================

The manuscript has been reassessed by an additional reviewer, and their comments are available below.

The reviewer raised additional concerns that need attention. They request additional discussion regarding the poor acceptance rate among men. The reviewer has noted that you may have participants who are minors. In your Methods section, please ensure you have also stated whether you obtained consent from parents or guardians of the minors included in the study or whether the research ethics committee or IRB specifically waived the need for their consent. The reviewer also provided suggestions to figure 2.

Could you please revise the manuscript to carefully address the concerns raised?

==============================

Please submit your revised manuscript by Aug 09 2026 11:59PM. If you will need more time than this to complete your revisions, please reply to this message or contact the journal office at globalpubhealth@plos.org. When you're ready to submit your revision, log on to https://www.editorialmanager.com/pgph/ and select the 'Submissions Needing Revision' folder to locate your manuscript file.

Please include the following items when submitting your revised manuscript:

- A letter that responds to each point raised by the editor and reviewer(s). You should upload this letter as a separate file labeled 'Response to Reviewers'.
- A marked-up copy of your manuscript that highlights changes made to the original version. You should upload this as a separate file labeled 'Revised Manuscript with Track Changes'.
- An unmarked version of your revised paper without tracked changes. You should upload this as a separate file labeled 'Manuscript'.

Guidelines for resubmitting your figure files are available below the reviewer comments at the end of this letter.

As the corresponding author, your ORCID iD is verified in the submission system and will appear in the published article. PLOS supports the use of ORCID, and we encourage all coauthors to register for an ORCID iD and use it as well. Please encourage your coauthors to verify their ORCID iD within the submission system before final acceptance, as unverified ORCID iDs will not appear in the published article. *Only*  the individual author can complete the verification step; PLOS staff *cannot*  verify ORCID iDs on behalf of authors.

We look forward to receiving your revised manuscript.

Kind regards,

Katherine Demi Kokkinias, Ph.D.

Staff Editor

PLOS Global Public Health

**Journal Requirements:**

If the reviewer comments include a recommendation to cite specific previously published works, please review and evaluate these publications to determine whether they are relevant and should be cited. There is no requirement to cite these works unless the editor has indicated otherwise.

**Additional Editor Comments (if provided):**

[Note: HTML markup is below. Please do not edit.]

Reviewers' comments:

Reviewer's Responses to Questions

**Comments to the Author**

Reviewer #2: All comments have been addressed

**********

publication criteria?

Reviewer #2: Yes

**********

3. Has the statistical analysis been performed appropriately and rigorously?

Reviewer #2: Yes

**********

4. Have the authors made all data underlying the findings in their manuscript fully available (please refer to the Data Availability Statement at the start of the manuscript PDF file)?

The PLOS Data policy

Reviewer #2: Yes

**********

5. Is the manuscript presented in an intelligible fashion and written in standard English?

Reviewer #2: Yes

**********

**Reviewer #2:**  Thank you for allowing me to review your manuscript. It is well written and timely to get us to the 95-95-95 targets.

Please consider the following:

1. Fig 2 needs sample size for each population so that it can stand alone.

2. The poor acceptance rate among men was not addressed adequately in the discussion or conclusions as it is a critical issue impacting on the female partner's retention on treatment as well as issues of GBV. Use of peers is a recognised strategy which could be addressed as one of the possible interventions to improve acceptance, linkage and retention.

3. Under ethical considerations, it is mentioned that no minors were included, however children <15years were included in the study. There needs to be clarification that consent was taken from the parents/legal guardians due to the higher age of consent in the country. Was no assent requested? Please clarify.

4. Minor formatting issues in the tables- all text in the cell need left justification- as it not consistent.

**********

what does this mean?). If published, this will include your full peer review and any attached files.

**Do you want your identity to be public for this peer review?**  If you choose “no”, your identity will remain anonymous but your review may still be made public.

For information about this choice, including consent withdrawal, please see our Privacy Policy

Reviewer #2: No

**********

[NOTE: If reviewer comments were submitted as an attachment file, they will be attached to this email and accessible via the submission site. Please log into your account, locate the manuscript record, and check for the action link "View Attachments". If this link does not appear, there are no attachment files.]

**Figure Resubmissions:**

# Author comment: Author response to Decision Letter 3

**DOI:** 10.1371/journal.pgph.0005461.r006

23 Jun 2026

Attachment Submitted filename: Response to Reviewers3.pdf

# Peer review: Decision Letter 3

**DOI:** 10.1371/journal.pgph.0005461.r007

**Authors:** Dumile Gumede

30 Jul 2026

PGPH-D-25-03261R3

RE-AIM evaluation of the national scale-up of community-based index case HIV testing in Guinea: Successes and Challenges

PLOS Global Public Health

Dear Dr. CAMARA,

Thank you for submitting your manuscript to PLOS Global Public Health. After careful consideration, we feel that it has merit but does not fully meet PLOS Global Public Health’s publication criteria as it currently stands. Therefore, we invite you to submit a revised version of the manuscript that addresses the points raised during the review process.

Please submit your revised manuscript by Aug 29 2026 11:59PM. If you will need more time than this to complete your revisions, please reply to this message or contact the journal office at globalpubhealth@plos.org. When you're ready to submit your revision, log on to https://www.editorialmanager.com/pgph/ and select the 'Submissions Needing Revision' folder to locate your manuscript file.

Please include the following items when submitting your revised manuscript:

- A letter that responds to each point raised by the editor and reviewer(s). You should upload this letter as a separate file labeled 'Response to Reviewers'.
- A marked-up copy of your manuscript that highlights changes made to the original version. You should upload this as a separate file labeled 'Revised Manuscript with Track Changes'.
- An unmarked version of your revised paper without tracked changes. You should upload this as a separate file labeled 'Manuscript'.

Guidelines for resubmitting your figure files are available below the reviewer comments at the end of this letter.

As the corresponding author, your ORCID iD is verified in the submission system and will appear in the published article. PLOS supports the use of ORCID, and we encourage all coauthors to register for an ORCID iD and use it as well. Please encourage your coauthors to verify their ORCID iD within the submission system before final acceptance, as unverified ORCID iDs will not appear in the published article. *Only*  the individual author can complete the verification step; PLOS staff *cannot*  verify ORCID iDs on behalf of authors.

We look forward to receiving your revised manuscript.

Kind regards,

Dumile Gumede, PhD (Health Promotion)

Academic Editor

PLOS Global Public Health

Journal Requirements:

1. If the reviewer comments include a recommendation to cite specific previously published works, please review and evaluate these publications to determine whether they are relevant and should be cited. There is no requirement to cite these works unless the editor has indicated otherwise.

2. Please review your reference list to ensure that it is complete and correct. If you have cited papers that have been retracted, please include the rationale for doing so in the manuscript text, or remove these references and replace them with relevant current references. Any changes to the reference list should be mentioned in the rebuttal letter that accompanies your revised manuscript. If you need to cite a retracted article, indicate the article’s retracted status in the References list and also include a citation and full reference for the retraction notice.

Additional Editor Comments (if provided):

[Note: HTML markup is below. Please do not edit.]

Reviewers' comments:

Reviewer's Responses to Questions

**Comments to the Author**

Reviewer #3: All comments have been addressed

Reviewer #4: (No Response)

**********

publication criteria?

Reviewer #3: Yes

Reviewer #4: Yes

**********

3. Has the statistical analysis been performed appropriately and rigorously?

Reviewer #3: Yes

Reviewer #4: Yes

**********

4. Have the authors made all data underlying the findings in their manuscript fully available (please refer to the Data Availability Statement at the start of the manuscript PDF file)?

The PLOS Data policy

Reviewer #3: Yes

Reviewer #4: Yes

**********

5. Is the manuscript presented in an intelligible fashion and written in standard English?

Reviewer #3: Yes

Reviewer #4: Yes

**********

Reviewer #3: (No Response)

Reviewer #4: Very interesting paper, health system integration of HIV programs is vital and identifying strengths and weaknesses is important. The RE-AIM framework was helpful in guiding the evaluation. With further clarification of methods and discussion the findings from this paper are very useful for HIV program development and scale-up. There are a lot of moving parts so a clear concise summary of findings in the conclusion would make the paper stronger.

Line 25-26: Switch the parenthesis to be around the acronym so it’s “Reach, Effectiveness, Adoption, Implementation, and Maintenance (RE-AIM)

Line 87: Delete “widely applied in public health”

Lines 78-92: Delete the following sentence “Evaluating complex interventions requires frameworks…occurs.” Then start the paragraph with the last two sentences in the paragraph. “The RE-AIM framework (Reach, Effectiveness, Adoption, Implementation, Maintenance) offers a structured approach to assess population coverage, behavioral……..and sustainability. This multidimensional lens…..and institutional anchoring.”

Line 101: Instead of “based on” use “using”

Line 106: Make “Quantitative were analyzed ….May 19, 2025.” its own sentence. Then make “Qualitative interviews and document reviews…..January 5, 2024.” its own sentence.

Line 122: Delete the information in the parenthesis, RE-AIM was already defined earlier

Line 243: Start a new paragraph for “Adoption”

Line 254: Start a new paragraph for “Maintenance”

Line 270: Please re-write as “For the subgroup analysis of HIV-positive individuals (Fig 2), a separate grouped binomial mixed-effects logistic regression model was fitted to estimate linkage to care.”

Line 272: Remove “specified as”

Line 352-356: The first two sentences should be in the data analysis section.

Line 382-385: Needs to go in the data analysis section

Line 457-459 & 465-467: These are the same sentences, one should be removed.

Line 507-508: The beginning sentence of this paragraph doesn’t really match the rest of the paragraph.

Line 534: New paragraph with “In our study…”

Line 547: State what the implementation pattern is, pattern in what? Refer to the result that shows this pattern.

Background

If possible, please include a figure or visual of the RE-AIM framework

Description of intervention

What was the further expansion from Phase 2 to Phase 3? What was the substantial increase from 17 districts to how many from Phase 2 to Phase 3? How is Phase 2 different from Phase 3?

Data Analysis

The data analysis paragraph is a little confusing. It might be clearer to put the “Operationalization of the RE-AIM Framework” under the Data Analysis section, then you can put what methods were used for each component of the RE-AIM framework in the section. How were outcome variables measured? How many models were run in total? What were the explanatory variables of interest? How were they defined? Which components utilized the qualitative findings?

Discussion

The discussion section is a little confusing and may benefit from dividing the third paragraph into smaller more focused paragraphs.

Discuss what implementation findings (tables 2 & 3) mean. Is this done in lines 534-546? If so, make it more explicit that is what is being discussed.

The HIV-positive women were identified via PMTCT services. Is it possible that low male participation could be due to recruiting through this service in particular, as the program may be seen as healthcare primarily for women and children and men do no see themselves as part of it?

Is it possible social norms around women and men’s health and HIV in Guinea play a role in low male engagement?

Conclusion

Do the findings show good, bad, or mixed performance?

**********

what does this mean?). If published, this will include your full peer review and any attached files.

**Do you want your identity to be public for this peer review?**  If you choose “no”, your identity will remain anonymous but your review may still be made public.

For information about this choice, including consent withdrawal, please see our Privacy Policy

Reviewer #3: **Yes:**  Eduarda Pimentel de Gusmao

Reviewer #4: No

**********

[NOTE: If reviewer comments were submitted as an attachment file, they will be attached to this email and accessible via the submission site. Please log into your account, locate the manuscript record, and check for the action link "View Attachments". If this link does not appear, there are no attachment files.]

Attachment Submitted filename: PGPH-D-25-03261_R3_EPG.pdf

Attachment Submitted filename: Review-PGPH-D-25-03261_R3.docx

# Author comment: Author response to Decision Letter 4

**DOI:** 10.1371/journal.pgph.0005461.r008

10 Aug 2026

Attachment Submitted filename: ResponseToReviewers4.pdf

# Peer review: Decision Letter 4

**DOI:** 10.1371/journal.pgph.0005461.r009

**Authors:** Dumile Gumede

22 Sep 2026

PGPH-D-25-03261R4

RE-AIM evaluation of the national scale-up of community-based index case HIV testing in Guinea: Successes and Challenges

PLOS Global Public Health

Dear Dr. CAMARA,

Thank you for submitting your manuscript to PLOS Global Public Health. After careful consideration, we feel that it has merit but does not fully meet PLOS Global Public Health’s publication criteria as it currently stands. Therefore, we invite you to submit a revised version of the manuscript that addresses the points raised during the review process.

I request the authors to define "95-95-95" at its first mention, either in the main text or in a footnote, to ensure clarity for readers who may be unfamiliar with the term prior to publication.

Please submit your revised manuscript by Oct 22 2026 11:59PM. If you will need more time than this to complete your revisions, please reply to this message or contact the journal office at globalpubhealth@plos.org. When you're ready to submit your revision, log on to https://www.editorialmanager.com/pgph/ and select the 'Submissions Needing Revision' folder to locate your manuscript file.

Please include the following items when submitting your revised manuscript:

- A letter that responds to each point raised by the editor and reviewer(s). You should upload this letter as a separate file labeled 'Response to Reviewers'.
- A marked-up copy of your manuscript that highlights changes made to the original version. You should upload this as a separate file labeled 'Revised Manuscript with Track Changes'.
- An unmarked version of your revised paper without tracked changes. You should upload this as a separate file labeled 'Manuscript'.

Guidelines for resubmitting your figure files are available below the reviewer comments at the end of this letter.

As the corresponding author, your ORCID iD is verified in the submission system and will appear in the published article. PLOS supports the use of ORCID, and we encourage all coauthors to register for an ORCID iD and use it as well. Please encourage your coauthors to verify their ORCID iD within the submission system before final acceptance, as unverified ORCID iDs will not appear in the published article. *Only*  the individual author can complete the verification step; PLOS staff *cannot*  verify ORCID iDs on behalf of authors.

We look forward to receiving your revised manuscript.

Kind regards,

Dumile Gumede, PhD (Health Promotion)

Academic Editor

PLOS Global Public Health

Journal Requirements:

If the reviewer comments include a recommendation to cite specific previously published works, please review and evaluate these publications to determine whether they are relevant and should be cited. There is no requirement to cite these works unless the editor has indicated otherwise.

Please review your reference list to ensure that it is complete and correct. If you have cited papers that have been retracted, please include the rationale for doing so in the manuscript text, or remove these references and replace them with relevant current references. Any changes to the reference list should be mentioned in the rebuttal letter that accompanies your revised manuscript. If you need to cite a retracted article, indicate the article’s retracted status in the References list and also include a citation and full reference for the retraction notice.

Additional Editor Comments (if provided):

[Note: HTML markup is below. Please do not edit.]

Reviewers' comments:

Reviewer's Responses to Questions

**Comments to the Author**

Reviewer #4: All comments have been addressed

Reviewer #5: (No Response)

**********

publication criteria?

Reviewer #4: Yes

Reviewer #5: Yes

**********

3. Has the statistical analysis been performed appropriately and rigorously?

Reviewer #4: Yes

Reviewer #5: Yes

**********

4. Have the authors made all data underlying the findings in their manuscript fully available (please refer to the Data Availability Statement at the start of the manuscript PDF file)?

The PLOS Data policy

Reviewer #4: (No Response)

Reviewer #5: Yes

**********

5. Is the manuscript presented in an intelligible fashion and written in standard English?

Reviewer #4: (No Response)

Reviewer #5: Yes

**********

Reviewer #4: (No Response)

Reviewer #5: This is my first review of the manuscript, which has been subjected to editorial review by other PLOS reviewers. Their comments seem to have been appropriately accepted and incorporated within this latest version.

The manuscript describes a major, multi-year research study that examines the scale up of nation wide community based index case HIV testing in Guinea. The study involved over 50,000 subjects and extended over nearly 4 years. The rationale, design, implementation, and statistical analysis have been presented in sufficient detail and the discussion of the findings, their significance, weaknesses in the study, and the limitations have all been presented in detail. The manuscript is well written, though lengthy.

The overall implications and possible limitations of using PMTCT subjects are well described, especially the disappointing impact on male partners and men in general. Within the Discussion section, the methodological limitations of the study are presented and capture most of the areas where interpretation of the results is unclear.

There is also a thorough discussion of the common and unavoidable challenges when attempting to provide positive health nterventions at scale, particularly due to the lack of stable funding sources, inherent operational weaknesses, supply chain failures, and ongoing data system constraints. The authors also provide the findings from comparable studies in other African settings, who reached similar conclusions.

This paper is valuable because it provides an excellent, detailed description of how to conduct a study on index case finding and the potential positive impact on both individual patient treatment, but also to decrease risk of transmission of a potentially lethal pathogen (HIV).

The Conclusion section is appropriately cautious about both the implications of the findings and the inherent challenges seen in low resource settings which are not likely to improve in the near future. I would have liked to see a further discussion on possible, realistic interventions and how countries can assess whether such large scale programs are worth the time and investments. However, I strongly endorse the authors' leadership in this area. Even in times where there is uncertain and unsteady support for country wide health interventions, particularly due to changes in development assistance, research staff should not cease in pursuing these areas of research. Times will change, and cooperative alliances between local governments and development partners will continue to look for proven community interventions.

SPECIFIC COMMENTS. (Using the line numbered manuscript)

Line 64: Please provide the full definition of the 95-95-95 UNAIDS targets in a footnote. Not every reader may be familiar with them.

**********

what does this mean?). If published, this will include your full peer review and any attached files.

**Do you want your identity to be public for this peer review?**  If you choose “no”, your identity will remain anonymous but your review may still be made public.

For information about this choice, including consent withdrawal, please see our Privacy Policy

Reviewer #4: No

Reviewer #5: **Yes:**  Paul De Lay, MD, DTM&H (Lond)

**********

[NOTE: If reviewer comments were submitted as an attachment file, they will be attached to this email and accessible via the submission site. Please log into your account, locate the manuscript record, and check for the action link "View Attachments". If this link does not appear, there are no attachment files.]

# Author comment: Author response to Decision Letter 5

**DOI:** 10.1371/journal.pgph.0005461.r010

29 Sep 2026

Attachment Submitted filename: ResponseToReviewer5.pdf

# Peer review: Decision Letter 5

**DOI:** 10.1371/journal.pgph.0005461.r011

**Authors:** Dumile Gumede

28 Oct 2026

RE-AIM evaluation of the national scale-up of community-based index case HIV testing in Guinea: Successes and Challenges

PGPH-D-25-03261R5

Dear Dr CAMARA,

We are pleased to inform you that your manuscript 'RE-AIM evaluation of the national scale-up of community-based index case HIV testing in Guinea: Successes and Challenges ' has been provisionally accepted for publication in PLOS Global Public Health.

Before your manuscript can be formally accepted you will need to complete some formatting changes, which you will receive in a follow up email. A member of our team will be in touch with a set of requests.

Please note that your manuscript will not be scheduled for publication until you have made the required changes, so a swift response is appreciated.

IMPORTANT: The editorial review process is now complete. PLOS will only permit corrections to spelling, formatting or significant scientific errors from this point onwards. Requests for major changes, or any which affect the scientific understanding of your work, will cause delays to the publication date of your manuscript.

If your institution or institutions have a press office, please notify them about your upcoming paper to help maximize its impact. If they'll be preparing press materials, please inform our press team as soon as possible -- no later than 48 hours after receiving the formal acceptance. Your manuscript will remain under strict press embargo until 2 pm Eastern Time on the date of publication. For more information, please contact globalpubhealth@plos.org.

Thank you again for supporting Open Access publishing; we are looking forward to publishing your work in PLOS Global Public Health.

Best regards,

Dumile Gumede, PhD (Health Promotion)

Academic Editor

PLOS Global Public Health

***********************************************************

Reviewer Comments (if any, and for reference):

Reviewer's Responses to Questions

**Comments to the Author**

Reviewer #5: All comments have been addressed

**********

publication criteria?

Reviewer #5: Yes

**********

3. Has the statistical analysis been performed appropriately and rigorously?

Reviewer #5: Yes

**********

4. Have the authors made all data underlying the findings in their manuscript fully available (please refer to the Data Availability Statement at the start of the manuscript PDF file)?

The PLOS Data policy

Reviewer #5: Yes

**********

5. Is the manuscript presented in an intelligible fashion and written in standard English?

Reviewer #5: Yes

**********

Reviewer #5: This is my second review of this manuscript. All of my previous comments have been addressed. Additionally, the authors have rewritten and revised other specific sections. These include "Data Quality and Cleaning," "Operationalization of the RE-AIM indicators", "Effectiveness", "Adoption", "Implementation' and in the Discussion section, there are revisions in the Discussion section on "mixed methods design." All of these revisions have further improved the clarity and readability of the manuscript. I now feel that the paper is ready for publication.

**********

what does this mean?). If published, this will include your full peer review and any attached files.

**Do you want your identity to be public for this peer review?**  If you choose “no”, your identity will remain anonymous but your review may still be made public.

For information about this choice, including consent withdrawal, please see our Privacy Policy

Reviewer #5: **Yes:**  Paul De Lay, MD, DTM&H (Lond)

**********
